# Metal-Free Oxidative Functionalization of Allylic Sulfones/Allylic Triflones with Water Participation Enabled by Hypervalent Iodine Reagents

**DOI:** 10.3390/molecules31162794

**Published:** 2026-08-11

**Authors:** Qian Tong, Li-Ting Xiao, Ming-Yang Gu, Xue-Qiang Chu, Danhua Ge

**Affiliations:** Technical Institute of Fluorochemistry, School of Chemistry and Molecular Engineering, Nanjing Tech University, Nanjing 211816, China; 202461105085@njtech.edu.cn (Q.T.);

**Keywords:** organosulfur compounds, organofluorides, water, sulfonyl ketones, triflyl allylic alcohols

## Abstract

A metal-free, efficient, iodine(III)-promoted oxidative transformation of allylic sulfones/allylic triflones with water participation for accessing sulfonyl ketones and triflyl allylic alcohols is reported. This strategy enables the selective construction of two SO_2_-containing compounds with chemical and biological significance, and its success mainly relies on the substitution effect of the starting materials. Water serves as an eco-friendly oxygen atom source. This transformation has excellent selectivity, broad substrate scope, mild reaction conditions, and ease of scale-up.

## 1. Introduction

Organosulfur compounds play a prominent role in clinical medicines, drug discovery, agrochemistry, organic synthesis, and materials science [1,2]. The sulfonyl group (-SO_2_-) is arguably among the most versatile sulfur-containing functionalities [3]. For example, sulfonyl ketones [4,5] and triflones [6,7] based on this privileged subunit have been found in diverse biologically and pharmaceutically relevant molecules. Meanwhile, as the “chemical chameleon”, sulfone derivatives are also important building blocks for facilitating new bond formations in organic synthesis [8]. As a result, considerable efforts have been directed towards the development of new methods to access these compounds with structural complexity and diversity [9,10,11,12].

**Figure 1:**
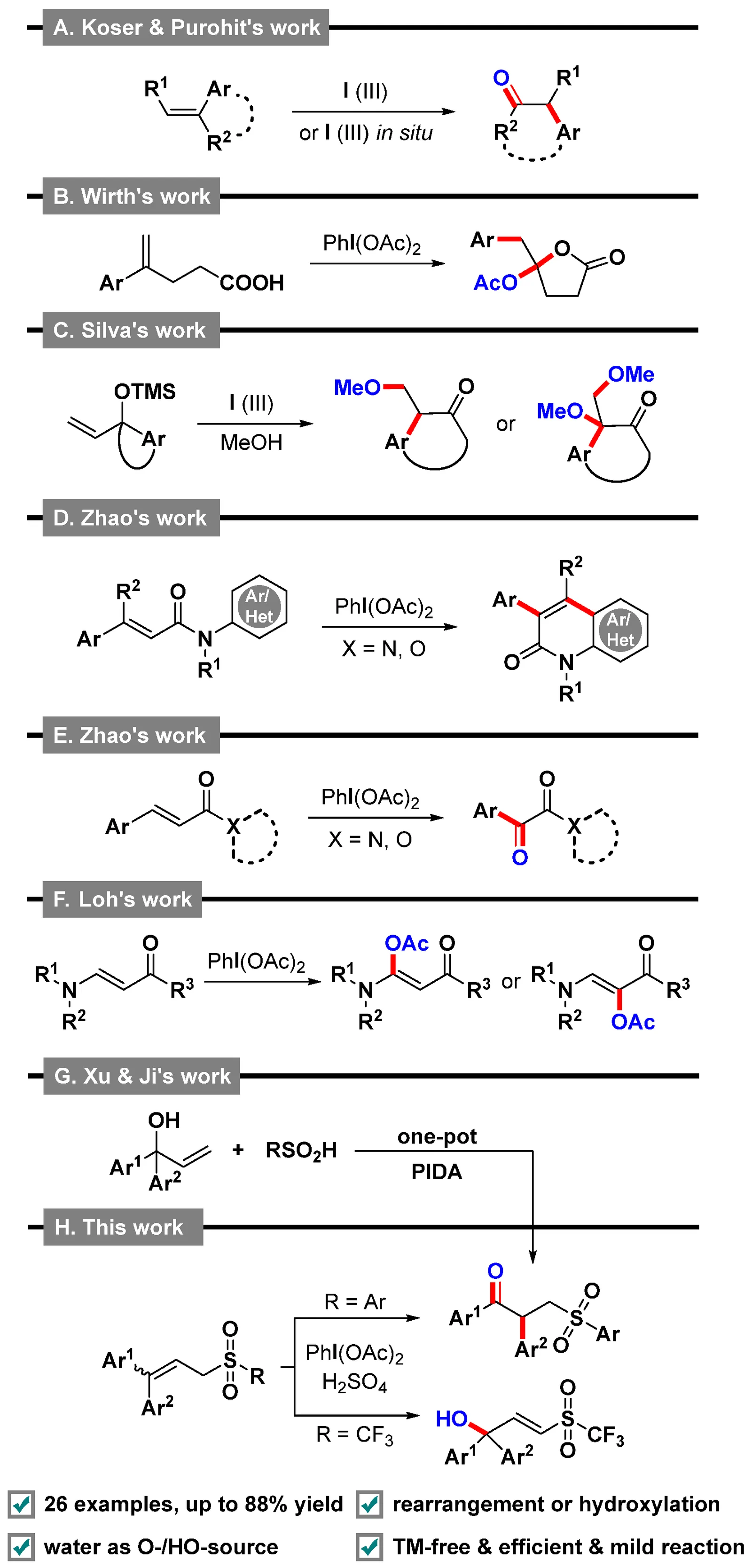
Iodine(III)-promoted oxidative functionalization of alkenes. (**A**) Koser and Purohit’s work [20]; (**B**) Wirth’s work [23]; (**C**) Silva’s work [24]; (**D**) Zhao’s work [26]; (**E**) Zhao’s work [27]; (**F**) Loh’s work [28]; (**G**) Xu and Ji’s work [9]; (**H**) this work.

Difunctionalization of unsaturated alkenes through the introduction of two versatile handles in a one-step manipulation represents one of the most attractive tools for the synthesis of valuable products [13,14]. The metal-free approach for direct oxidative functionalization of alkenes has presented an alternative option for the synthesis of useful molecules from simple raw materials [15,16]. In this context, hypervalent iodine(III) reagent, as a low-toxic, readily available, and environmentally friendly oxidant, has been extensively utilized as a unique electrophile to active the double bonds in alkenes [17,18,19]. For example, oxidative aryl migration [20,21], ring contraction [22,23], ring expansion [24,25], and additional transformations of alkenes [26,27,28,29], have been achieved by taking advantage of the distinctive chemical reactivity and structural features of hypervalent iodine(III) reagents during the past few decades. In 1984, Koser and co-workers disclosed the first hydroxytosyloxy iodobenzene (HTIB)-mediated oxidative rearrangement of 1,1-diphenylethylenes (Figure 1A) [20]. Wirth’s group developed an aryl-λ_3_-iodane-promoted lactonization followed by a rearrangement with phenonium ion participation (Figure 1B) [23]. In 2008, an iodine(III)-mediated ring expansion of 1-vinylcycloalkanol derivatives for the synthesis of seven-membered ring was developed by Silva’s group (Figure 1C) [24]. In addition, tandem metal-free oxidative aryl migration/C-C bond-formation reactions, promoted by hypervalent iodine reagents, were reported by Zhao et al. (Figure 1D,E) [26,27]. Very recently, Loh and Jiang discussed a site-selective acyloxylation of enamines by using PhI(OAc)_2_ under metal-free conditions to afford (*E*)-vinyl acetate derivatives (Figure 1F) [28]. Although these significant contributions were made in this area, the straightforward and efficient method pertaining to the functionalization of unsaturated sulfonyl compounds [30,31] for the divergent construction of highly valuable sulfone derivatives is relatively underdeveloped. Importantly, the momentous link between sulfonyl/trifluoromethyl sulfonyl groups and bioactivity has led us to incorporate as many such core scaffolds as possible into target molecules.

Previously, a metal-free tandem method by employing a sulfonylation of aryl allylic alcohols followed by a PIDA-promoted oxidative functionalization has been achieved by Xu/Ji’s group via a one-pot operation (Figure 1G) [9]. Encouraged by the recent research interests in oxidative transformations [32,33,34,35], herein we developed a metal-free strategy for accessing a variety of multi-substituted β-sulfonyl ketones and (trifluoromethyl)sulfonylated allylic alcohols with good functional group tolerance by employing (diacetoxyiodo)benzene (PIDA) as the oxidant (Figure 1H). Allylic sulfone compounds could be smoothly converted to β-sulfonyl ketones through C(sp^2^)-C(sp^2^) bond and C=O bond formations in one pot, along with a simultaneous aryl rearrangement. Also, hydroxylation of allylic triflones at the allylic position was realized under the mild reaction conditions. The success of this strategy could be attributed to the combination of hypervalent iodine reagents and a strong acid with water participation. In this paper, the substrate scope of allylic sulfones and the tolerance of the functional groups have been expanded. From the viewpoint of green and sustainable chemistry, this approach has the following features: (i) metal-catalyst/additive and multi-step operation are not necessary; (ii) water serves as an eco-friendly oxygen atom source [36,37,38,39,40,41]; (iii) the reactions proceed in a highly rapid (within 20 min) and efficient manner (up to 88% yield); (iv) divergent synthesis of two biologically and pharmaceutically active SO_2_-containing products.

## 2. Results and Discussion

**Scheme 1:**
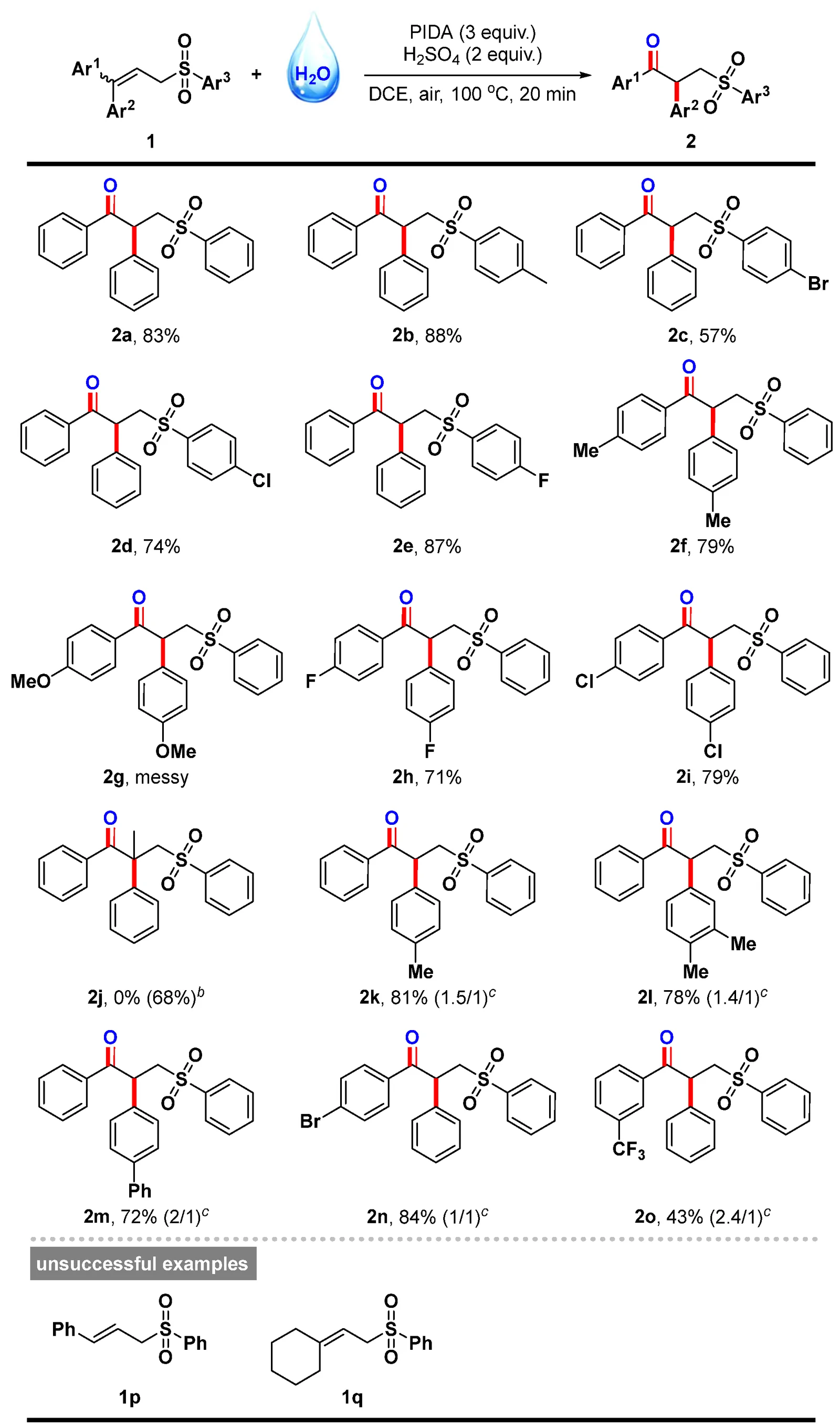
Substrate scope of various allylic sulfones **1***^a^*. *^a^* Reaction conditions: **1** (0.3 mmol), PIDA (0.9 mmol), and H_2_SO_4_ (wt% = 98%, 0.6 mmol) in DCE (4 mL) at 100 °C for 20 min under air; isolated yield. *^b^* Recovery of 1j. *^c^* Ratio of isomers was determined by ^1^H NMR analysis.

According to the previous report [9], we selected (3-(phenylsulfonyl)prop-1-ene-1,1-diyl)dibenzene (**1a**) as the model substrate to optimize the reaction conditions. The substrate was treated with (diacetoxyiodo)benzene (3.0 equiv.; PIDA) and H_2_SO_4_ (2.0 equiv.; added with caution) in DCE (4 mL) at 100 °C under air, and the results are summarized in Scheme 1. Interestingly, the reaction could proceed in a rapid and efficient manner (within 20 min), affording the corresponding product **2a** in 83% isolated yield.

Next, we continued our task of exploring the substrate scope of this metal-free protocol with respect to substituted allylic sulfones. As shown in Scheme 1, it was found that sulfones **1** containing different substituents on the arylsulfonyl moieties could efficiently undergo the oxidative migration to give the expected β-sulfonyl ketones **2a**–**2e** in 57–88% yields. Useful substituents and functionalities such as methyl group and halogen (e.g., Br, Cl, or F) were compatible with the reaction conditions (**2b**–**2e**). Meanwhile, allylic sulfones **1** possessing substituents of varying steric hindrance and electronic character on the phenyl group adjacent to the double bonds were successfully converted into the ketone derivatives in moderate to good yields (**2f**, **2h**–**2i**, and **2k**–**2o**). Additionally, for the asymmetrical substrates **1k**–**1o** with two different aryl groups, electron-rich aryl rings were more easily migrated than electron-poor aryl rings. These results suggested that the reaction course might go through a carbocation pathway. Notably, functional groups, such as Me, halogen (e.g., F, Cl, or Br), and CF_3_ group, could be tolerated. However, the sensitive 1,2-bis(4-methoxyphenyl)-3-(phenylsulfonyl)propan-1-one (**2j**) was not detected, and the reaction system was very messy. Additionally, steric hindrance had a dramatic detrimental effect on aryl rearrangement because product **2j** possessing an α-quaternary center was not formed. We also attempted to extend this protocol to mono-aryl-substituted and alkyl-substituted allylic sulfones. Disappointingly, substrates **1p** and **1q** were proven to be unsuitable candidates for the present oxidative migration/C-C bond cleavage process. It should be noted that the two aryl groups were essential for the rearrangement probably due to the stability of the related intermediates.

**Scheme 2:**
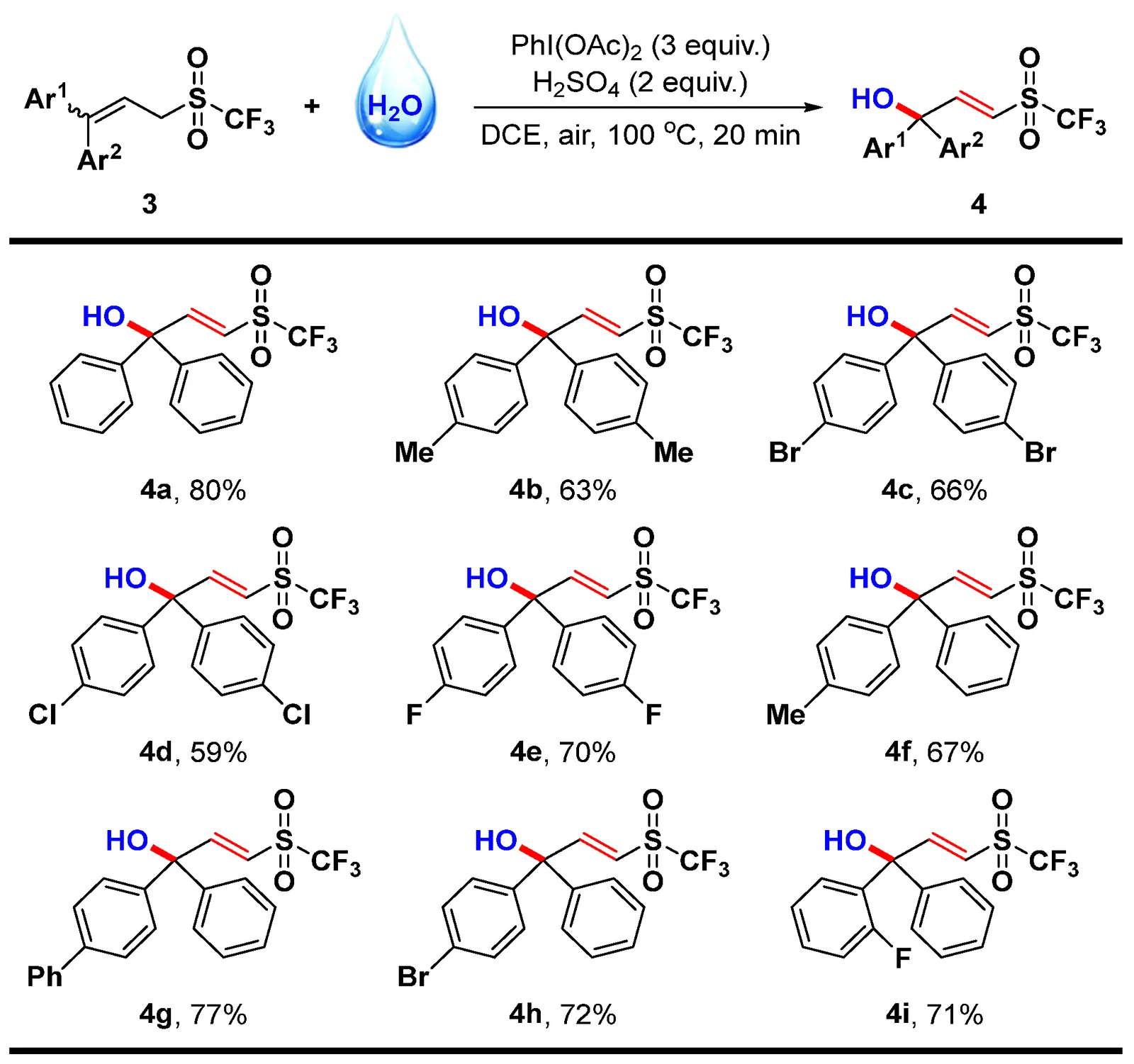
Substrate scope of various diaryl allylic triflones **3***^a^*. *^a^* Reaction conditions: **1** (0.3 mmol), PIDA (0.9 mmol), and H_2_SO_4_ (wt% = 98%, 0.6 mmol) in DCE (4 mL) at 100 °C for 20 min under air; isolated yield.

Encouraged by the above transformations, the synthetic potential of the strategy was extended to the oxidative functionalization of various allylic triflones **3**, and the results are summarized in Scheme 2. Unexpectedly, when the arylsulfonyl moiety of the substrate was replaced by (trifluoromethyl)sulfonyl group, the title reaction of **3a** predominantly afforded a hydroxylated triflyl allylic alcohol **4a** in 80% yield and only gave rise to the sulfonyl ketone in a relatively low yield. We continued to study the generality of the reaction by employing an array of substituted allylic triflones **3b**–**3i** as substrates. In these cases, the reactions proceeded smoothly to afford the products **4b**–**4i** in 59–80% yields. Besides (3-((trifluoromethyl)sulfonyl)prop-1-ene-1,1-diyl)diarenes **3a**–**3e** bearing two identical aryl rings, asymmetrical substrates **3f**–**3i** possessing Me, Ph, or Br substituents could also be participated in the reactions, affording the desired products **4f**–**4h** in 67–77% yields. Notably, when F-substituent was present at the *ortho* position of the phenyl ring, the steric hindrance did not have a dramatic detrimental effect on the reaction performance (**4i**, 71% yield). The present approach provided chemists a convenient platform for synthesizing diverse vinyl triflones that featured exclusive *E* selectivity.

**Scheme 3:**
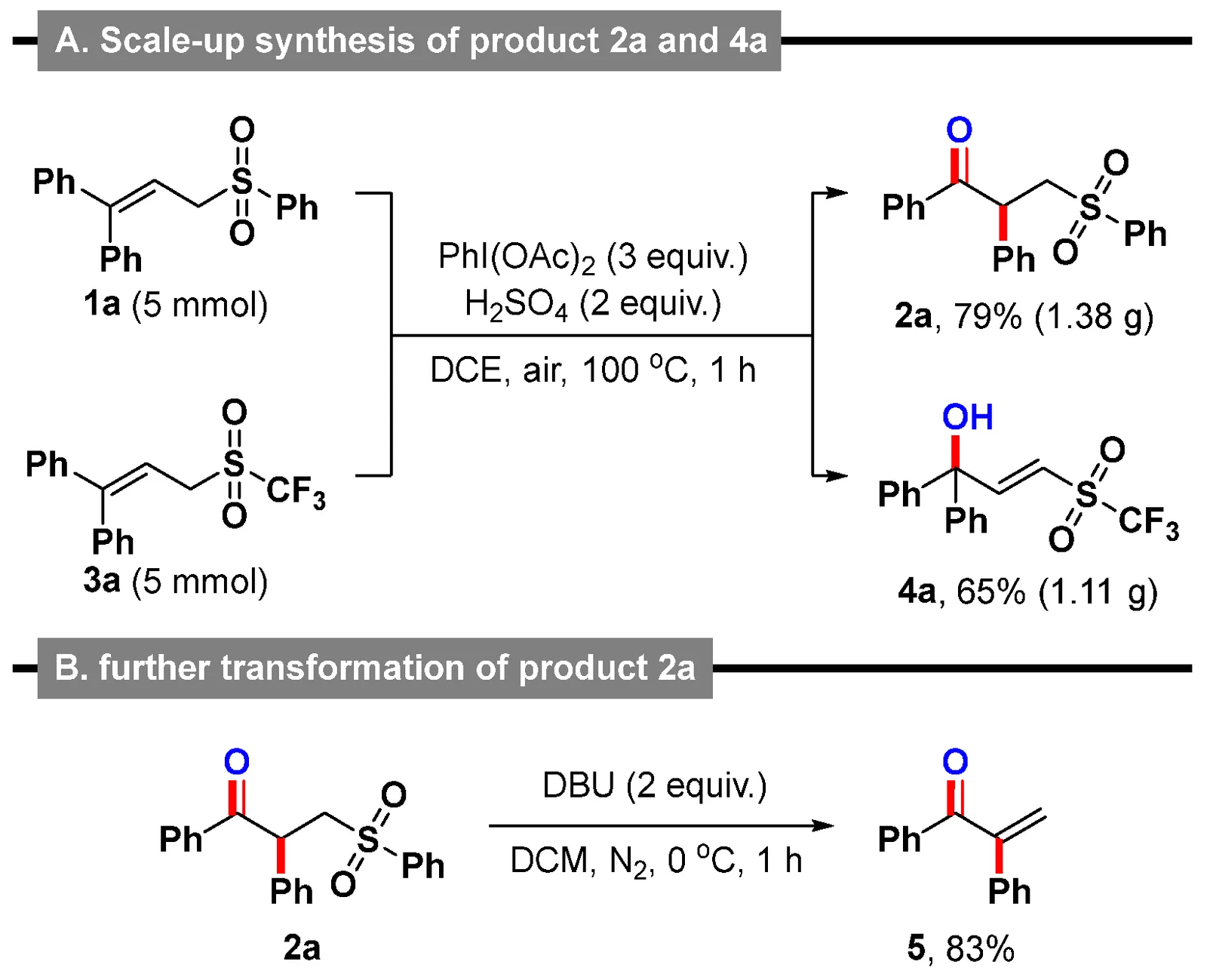
Scale-up synthesis and further transformation.

The feasibility of performing the reactions on a scale-up synthesis of 1,2-diphenyl-3-(phenylsulfonyl)propan-1-one (**2a**, 79%, 1.38 g) and (*E*)-1,1-diphenyl-3-((trifluoromethyl)sulfonyl)prop-2-en-1-ol (**4a**, 65% 1.11 g) potentially allows the further application of this iodine(III)-promoted method (Scheme 3A). Furthermore, the desulfonylation of the resulting **2a** occurred smoothly in the presence of DBU (2.0 equiv.) at 0 °C in DCM to give 1,2-diphenylprop-2-en-1-one (**5**) in 83% yield (Scheme 3B).

**Scheme 4:**
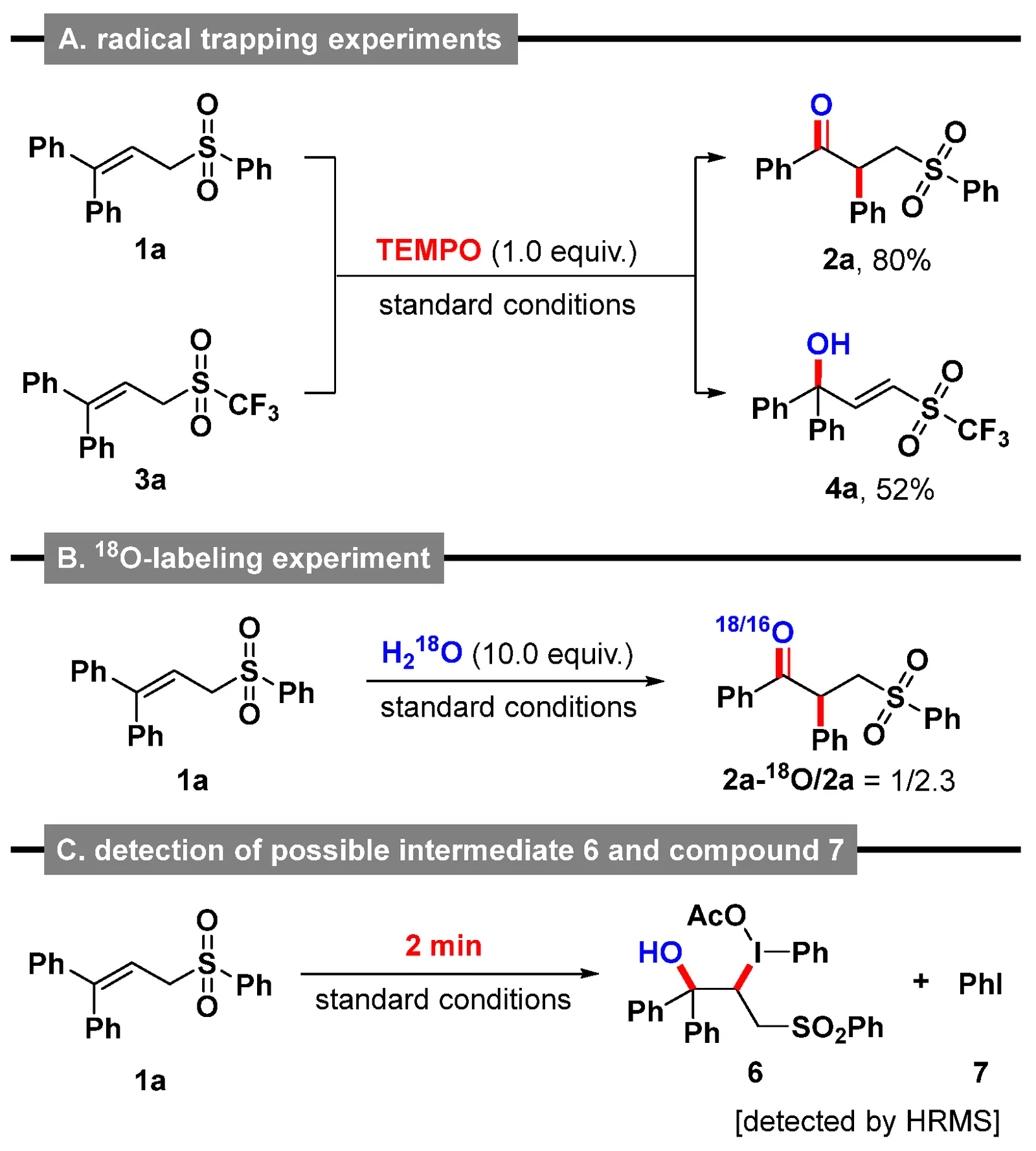
Mechanistic studies.

To gain more insight into the mechanism, a series of control experiments were conducted, and the results are summarized in Scheme 4. First, the reactions of (3-(phenylsulfonyl)prop-1-ene-1,1-diyl)dibenzene (**1a**) or (3-((trifluoromethyl)sulfonyl)prop-1-ene-1,1-diyl)dibenzene (**3a**) in the presence of the stoichiometric radical inhibitor 2,2,6,6-tetramethylpiperidin-1-oxyl (1 equiv.; TEMPO) were not inhibited, furnishing the corresponding products **2a** and **4a** in 80% and 52% yields, respectively (Scheme 4A). These results indicated that radical intermediates might not be involved in the reaction process. Second, water participation was confirmed by further ^18^O-labeling experimental evidence (Scheme 4B, see the Supporting Information for more details). Third, the generation of hypervalent iodine-adduct **6** and iodobenzene (**7**) were determined by HRMS analysis, when the reaction of **1a** was performed for 2 min under the standard conditions (Scheme 4C). This result suggested that PIDA was essential for the activation of the double bond of allylic sulfones.

**Scheme 5:**
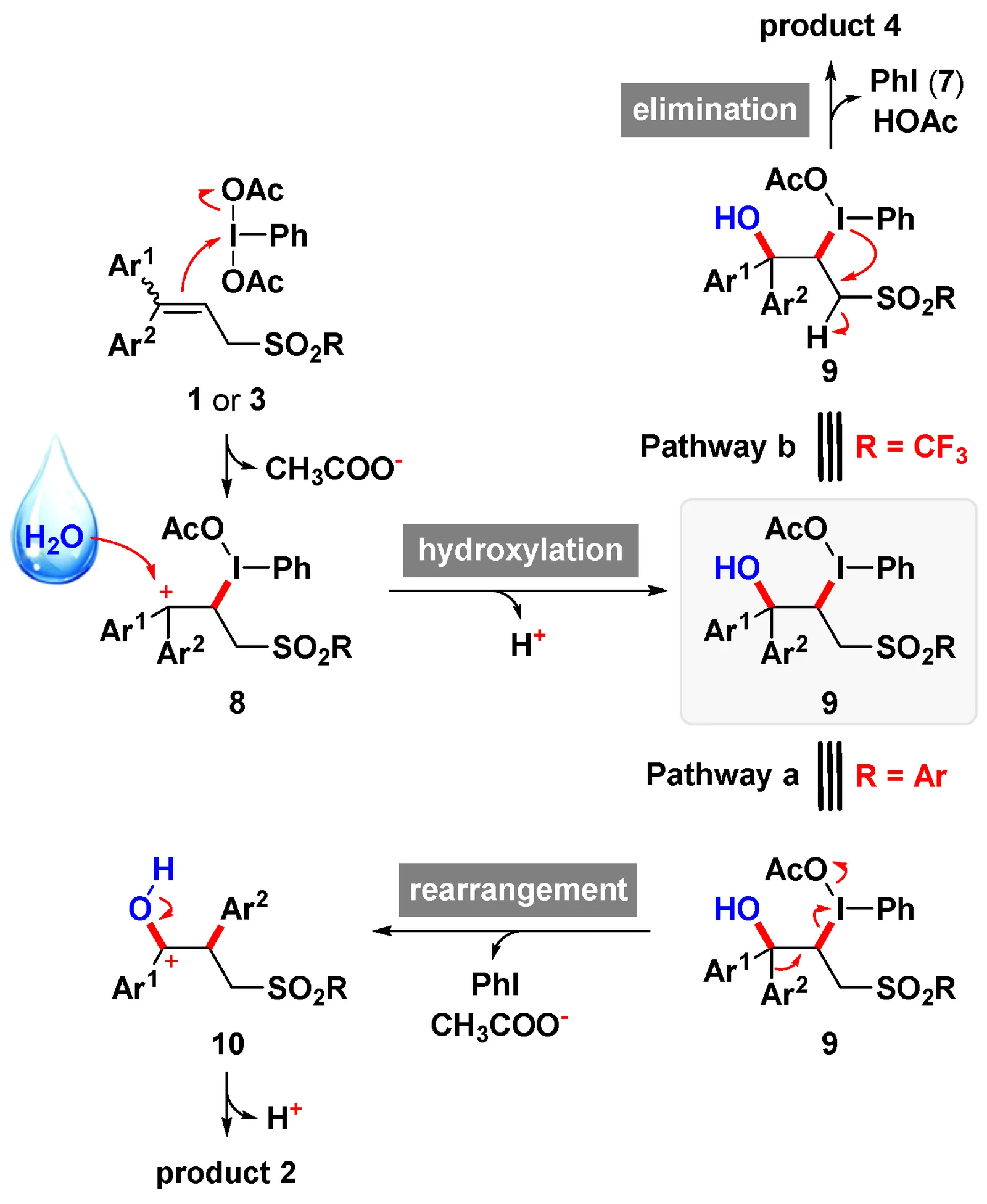
Proposed mechanism.

On the basis of the observations above and the literature survey [17,18,19,20,21,22,23,24,25,26,27,28,29], the following plausible mechanism is proposed for this oxidative transformation (Scheme 5). Initially, with the activation through protonation by H_2_SO_4_ [26,27,28,29], the iodine center of PIDA becomes very electrophilic and readily reacts with the unsaturated moiety of allylic sulfones/allylic triflones to produce carbocation **8**. It is entirely reasonable to believe that the reaction regioselectivity is largely determined by the stability of the resulting tertiary reactive intermediate **8**. Subsequently, the nucleophilic attack on the benzylic carbocation by water forms the key intermediate **9**. In principal, two possible pathways could be envisioned with diacritical R groups of hypervalent iodine-adduct **9**. Namely, when the R group is an aryl substituent (R = aryl), a 1,2-aryl rearrangement occurs via the elimination of PhI and acetate anion through intermediate **10**, which further releases a proton to afford the sulfonyl ketone **2**. Alternatively, intermediate **9** undergoes β-fission of C-I bond to release the corresponding product **4**, iodobenzene, and acetic acid when the substituent is a trifluoromethyl group (R = CF_3_). We believe that the strong electron-withdrawing effect of SO_2_CF_3_ (compared to SO_2_Ar) increases the corresponding acidity, which makes the elimination process much easier.

## 3. Materials and Methods

### 3.1. General Information

All reactions were carried out in flame-dried glassware under an atmosphere of dry N_2_ with the rigid exclusion of air and moisture using standard Schlenk techniques. All chemicals were purchased from commercial sources and used as received unless otherwise specified. ^1^H, ^19^F, and ^13^C NMR spectra were recorded in CDCl_3_ or DMSO-*d*_6_ on Bruker Avance (Bruker BioSpin GmbH, Rheinstetten, Germany) or Joel (JEOL Ltd., Tokyo, Japan) 400 MHz spectrometers. NMR splitting patterns are designated as singlet (s), doublet (d), triplet (t), quartet (q), multiplet (m), doublet of doublets (dd), doublet of triplets (dt), doublet of quartets (dq), triplet of doublets (td), triplet of triplets (tt), quartet of doublets (qd), and doublet of doublet of doublets (ddd), etc. The chemical shifts (*δ*) are reported in ppm and coupling constants (*J*) in Hz. High resolution mass spectrometry (HRMS) data were obtained on a Waters LC-TOF mass spectrometer (Xevo G2-XS QTof; Waters Corporation, Massachusetts, United States) using electrospray ionization (ESI) in positive or negative mode. Column chromatography was generally performed on silica gel (300–400 mesh) and reactions were monitored by thin layer chromatography (TLC; Wanqing, Nanjing, China) using UV light to visualize the course of the reactions.

### 3.2. General Procedure for the Synthesis of β-Sulfonyl Ketones ***2***

A solution of allylic sulfone **1** (0.3 mmol), PhI(OAc)_2_ (290 mg, 0.9 mmol, PIDA), and H_2_SO_4_ (60 mg, 0.6 mmol, wt% = 98%) in DCE (4 mL) was stirred at 100 °C (oil bath) under air for 20 min. Upon completion of the reaction (indicated by TLC), the reaction was then quenched by saturated NaHCO_3_ solution (20 mL) and extracted with EtOAc (20 mL × 3). The organic layer was washed with saturated brine, dried over MgSO_4_, filtered, and concentrated under reduced pressure. The crude product was purified by flash column chromatography (silica gel; 300–400 mesh) using petroleum ether (PE)/ethyl acetate (EA) (100/1~20/1) as eluent to afford the pure products **2**.

### 3.3. General Procedure for the Synthesis of Triflyl Allylic Alcohols ***4***

A solution of allylic triflone **3** (0.3 mmol), PhI(OAc)_2_ (290 mg, 0.9 mmol, PIDA), and H_2_SO_4_ (60 mg, 0.6 mmol, wt% = 98%) in DCE (4 mL) was stirred at 100 °C (oil bath) under air for 20 min. Upon completion of the reaction (indicated by TLC), the reaction was then quenched by saturated NaHCO_3_ solution (20 mL) and extracted with EtOAc (20 mL × 3). The organic layer was washed with saturated brine, dried over MgSO_4_, filtered, and concentrated under reduced pressure. The crude product was purified by flash column chromatography (silica gel; 300–400 mesh) using petroleum ether (PE)/ethyl acetate (EA) (100/1~6/1) as eluent to afford the pure products **4**.

### 3.4. General Procedure for the Scale-Up Synthesis of Product ***2a***

A solution of (3-(phenylsulfonyl)prop-1-ene-1,1-diyl)dibenzene (1672 mg, 5 mmol, **1a**), PhI(OAc)_2_ (4832 mg, 15 mmol, PIDA), and H_2_SO_4_ (1000 mg, 10 mmol, wt% = 98%) in DCE (20 mL) was stirred at 100 °C (oil bath) under air for 20 min. Upon completion of the reaction (indicated by TLC), the reaction was then quenched by saturated NaHCO_3_ solution (100 mL) and extracted with EtOAc (100 mL × 3). The organic layer was washed with saturated brine twice, dried over MgSO_4_, filtered, and concentrated under reduced pressure. The crude product was purified by flash column chromatography (silica gel; 300–400 mesh) using petroleum ether (PE)/ethyl acetate (EA) (100/1~20/1) as eluent to afford the pure product **2a** (1384 mg, 79% yield).

### 3.5. General Procedure for the Scale-Up Synthesis of Product ***4a***

A solution of (3-((trifluoromethyl)sulfonyl)prop-1-ene-1,1-diyl)dibenzene (1632 mg, 5 mmol, **3a**), PhI(OAc)_2_ (4832 mg, 15 mmol, PIDA), and H_2_SO_4_ (1000 mg, 10 mmol, wt% = 98%) in DCE (20 mL) was stirred at 100 °C (oil bath) under air for 20 min. Upon completion of the reaction (indicated by TLC), the reaction was then quenched by saturated NaHCO_3_ solution (100 mL) and extracted with EtOAc (100 mL × 3). The organic layer was washed with saturated brine twice, dried over MgSO_4_, filtered, and concentrated under reduced pressure. The crude product was purified by flash column chromatography (silica gel; 300–400 mesh) using petroleum ether (PE)/ethyl acetate (EA) (100/1~6/1) as eluent to afford the pure product **4a** (1113 mg, 65% yield).

### 3.6. Further Transformation of Product ***2a***

A solution of (3-(phenylsulfonyl)prop-1-ene-1,1-diyl)dibenzene (105 mg, 0.3 mmol, **1a**) and DBU (93 mg, 0.6 mmol) in DCM (2 mL) was stirred at 0 °C (ice bath) under N_2_ for 1 h. Upon completion of the reaction (indicated by TLC), the reaction was then quenched by saturated NaCl solution (20 mL) and extracted with EtOAc (20 mL × 3). The organic layer was washed with saturated brine twice, dried over MgSO_4_, filtered, and concentrated under reduced pressure. The crude product was purified by flash column chromatography (silica gel; 300–400 mesh) using petroleum ether (PE)/ethyl acetate (EA) (100/1~6/1) as eluent to afford the pure product **5** (52 mg, 83% yield).

**1,2-Diphenyl-3-(phenylsulfonyl)propan-1-one (2a):** Yield = 83% (87 mg). White solid. M.p. 107–108 °C. **^1^H NMR** (400 MHz, CDCl_3_): *δ* = 7.95–7.87 (m, 2H), 7.86–7.78 (m, 2H), 7.62–7.54 (m, 1H), 7.53–7.42 (m, 3H), 7.39 (dd, *J* = 8.4, 7.1 Hz, 2H), 7.24 (d, *J* = 3.5 Hz, 4H), 7.22–7.16 (m, 1H), 5.30 (dd, *J* = 8.7, 3.8 Hz, 1H), 4.43 (dd, *J* = 14.2, 8.7 Hz, 1H), 3.45 (dd, *J* = 14.2, 3.8 Hz, 1H) ppm. **^13^C NMR** (100 MHz, CDCl_3_) *δ* = 195.8, 139.4, 136.4, 135.4, 133.7, 133.4, 129.4, 129.2, 128.9, 128.6, 128.1, 128.0, 59.2, 47.5 ppm. **HRMS** m/z: calcd for C_21_H_18_NaO_3_S [M+Na]^+^ 373.0869, found: 373.0874.

**1,2-Diphenyl-3-tosylpropan-1-one (2b):** Yield = 88% (96 mg). Colorless oil. **^1^H NMR** (400 MHz, CDCl_3_): *δ* = 7.92 (dd, *J* = 7.7, 2.1 Hz, 2H), 7.72 (d, *J* = 8.3 Hz, 2H), 7.52 (t, *J* = 7.4 Hz, 1H), 7.41 (t, *J* = 7.7 Hz, 2H), 7.36–7.31 (m, 1H), 7.27–7.17 (m, 6H), 5.31 (dd, *J* = 8.8, 3.6 Hz, 1H), 4.44 (dd, *J* = 14.1, 8.8 Hz, 1H), 3.45 (dd, *J* = 14.2, 3.6 Hz, 1H), 2.41 (s, 3H) ppm. **^13^C NMR** (100 MHz, CDCl_3_) *δ* = 195.8, 144.7, 136.5, 136.3, 135.4, 133.4, 129.7, 129.3, 128.8, 128.5, 128.1, 128.0, 127.9, 59.3, 47.5, 21.5 ppm. **HRMS** m/z: calcd for C_22_H_20_NaO_3_S [M+Na]^+^ 387.1025, found: 387.1033.

**3-(4-Bromophenylsulfonyl)-1,2-diphenylpropan-1-one (2c):** Yield = 57% (73 mg). White solid. M.p. 103–104 °C. **^1^H NMR** (400 MHz, CDCl_3_): *δ* = δ 7.93–7.85 (m, 2H), 7.70–7.61 (m, 2H), 7.61–7.46 (m, 2H), 7.44–7.35 (m, 2H), 7.23 (dd, *J* = 5.2, 1.4 Hz, 5H), 5.27 (dd, *J* = 9.0, 4.3 Hz, 1H), 4.40 (dd, *J* = 14.5, 8.7 Hz, 1H), 3.48 (dd, *J* = 14.4, 4.2 Hz, 1H) ppm. **^13^C NMR** (100 MHz, CDCl_3_) *δ* = 195.8, 138.5, 136.2, 135.4, 133.7, 132.6, 129.7, 129.6, 129.2, 129.0, 128.8, 128.3, 128.2, 59.4, 47.8 ppm. **HRMS** m/z: calcd for C_21_H_17_^79^BrNaO_3_S [M+Na]^+^ 450.9974, found: 450.9977.

**3-(4-Chlorophenylsulfonyl)-1,2-diphenylpropan-1-one (2d):** Yield = 74% (85 mg). White solid. M.p. 92–93 °C. **^1^H NMR** (400 MHz, CDCl_3_): *δ* = 7.93–7.86 (m, 2H), 7.76–7.69 (m, 2H), 7.53–7.48 (m, 1H), 7.42–7.36 (m, 4H), 7.26–7.18 (m, 5H), 5.28 (dd, *J* = 8.7, 4.0 Hz, 1H), 4.40 (dd, *J* = 14.3, 8.7 Hz, 1H), 3.48 (dd, *J* = 14.3, 4.0 Hz, 1H) ppm. **^13^C NMR** (100 MHz, CDCl_3_) *δ* = 195.7, 140.4, 137.8, 136.1, 135.3, 133.5, 129.5, 129.4, 128.8, 128.6, 128.1, 128.1, 59.3, 47.6 ppm. **HRMS** m/z: calcd for C_21_H_17_ClNaO_3_S [M+Na]^+^ 407.0479, found: 407.0484.

**3-(4-Fluorophenylsulfonyl)-1,2-diphenylpropan-1-one (2e):** Yield = 87% (96 mg). Colorless oil. **^1^H NMR** (400 MHz, CDCl_3_): *δ* = 7.96–7.83 (m, 4H), 7.53 (t, *J* = 7.4 Hz, 1H), 7.44–7.26 (m, 7H), 7.12 (t, *J* = 8.5 Hz, 2H), 5.31 (dd, *J* = 8.6, 4.0 Hz, 1H), 4.43 (dd, *J* = 14.2, 8.7 Hz, 1H), 3.50 (dd, *J* = 14.2, 4.0 Hz, 1H) ppm. **^13^C NMR** (100 MHz, CDCl_3_) *δ* = 195.7, 135.8 (d, *J* = 77.2 Hz), 135.7 (d, *J* = 85.8 Hz), 133.5, 131.5 (d, *J* = 9.5 Hz), 130.9 (d, *J* = 9.6 Hz), 129.4, 129.1 (d, *J* = 13.9 Hz), 128.7 (d, *J* = 18.3 Hz), 128.1, 127.9 (d, *J* = 23.3 Hz), 59.3, 47.6 ppm. **HRMS** m/z: calcd for C_21_H_17_FNaO_3_S [M+Na]^+^ 391.0775, found: 391.0781.

**3-(Phenylsulfonyl)-1,2-dip-tolylpropan-1-one (2f):** Yield = 79% (90 mg). White solid. M.p. 108–109 °C. **^1^H NMR** (400 MHz, CDCl_3_): *δ* = 7.81 (td, *J* = 5.9, 2.7 Hz, 4H), 7.59–7.54 (m, 1H), 7.44 (t, *J* = 7.8 Hz, 2H), 7.17 (d, *J* = 8.0 Hz, 2H), 7.10 (d, *J* = 8.1 Hz, 2H), 7.03 (d, *J* = 8.0 Hz, 2H), 5.24 (dd, *J* = 8.5, 4.0 Hz, 1H), 4.38 (dd, *J* = 14.2, 8.5 Hz, 1H), 3.43 (dd, *J* = 14.2, 4.1 Hz, 1H), 2.35 (s, 3H), 2.24 (s, 3H) ppm. **^13^C NMR** (100 MHz, CDCl_3_): *δ* = 195.4, 144.3, 139.5, 137.7, 133.6, 133.5, 132.9, 130.0, 129.3, 129.1, 129.0, 128.0, 127.9, 59.2, 47.0, 21.6, 21.0 ppm. **HRMS** m/z: calcd for C_23_H_22_NaO_3_S [M+Na]^+^ 401.1182, found: 401.1187.

**1,2-Bis(4-fluorophenyl)-3-(phenylsulfonyl)propan-1-one (2h):** Yield = 71% (82 mg). Colorless oil. **^1^H NMR** (400 MHz, CDCl_3_): *δ* = 7.96–7.89 (m, 2H), 7.85–7.78 (m, 2H), 7.62–7.55 (m, 1H), 7.47 (t, *J* = 7.8 Hz, 2H), 7.24–7.17 (m, 2H), 7.10–7.03 (m, 2H), 6.99–6.89 (m, 2H), 5.26 (dd, *J* = 8.4, 4.3 Hz, 1H), 4.34 (dd, *J* = 14.1, 8.4 Hz, 1H), 3.44 (dd, *J* = 14.2, 4.3 Hz, 1H) ppm. **^13^C NMR** (100 MHz, CDCl_3_): *δ* = 194.3, 165.8 (d, *J* = 254.9 Hz), 162.2 (d, *J* = 246.6 Hz), 139.3, 133.8, 131.9 (d, *J* = 3.3 Hz), 131.5 (d, *J* = 9.3 Hz), 129.8 (d, *J* = 8.2 Hz), 129.2, 129.2, 127.9, 116.4 (d, *J* = 21.6 Hz), 115.9 (d, *J* = 21.8 Hz), 59.1, 46.5 ppm. **HRMS** m/z: calcd for C_21_H_16_F_2_NaO_3_S [M+Na]^+^ 409.0680, found: 409.0682.

**1,2-Bis(4-chlorophenyl)-3-(phenylsulfonyl)propan-1-on*e* (2i):** Yield = 79% (99 mg). White solid. M.p. 41–42 °C. **^1^H NMR** (400 MHz, CDCl_3_): *δ* = 7.80–7.70 (m, 3H), 7.57–7.51 (m, 1H), 7.43–7.38 (m, 2H), 7.32–7.29 (m, 2H), 7.19–7.14 (m, 3H), 7.08 (d, *J* = 4.3 Hz, 2H), 5.16 (dd, *J* = 8.3, 4.4 Hz, 1H), 4.29–4.13 (m, 1H), 3.37 (dd, *J* = 14.1, 4.4 Hz, 1H). ppm. **^13^C NMR** (100 MHz, CDCl_3_): *δ* = 194.5, 140.2, 139.2, 134.4, 134.3, 133.8, 133.4, 130.2, 129.6, 129.4, 129.2, 129.1, 127.9, 58.9, 46.7 ppm. **HRMS** m/z: calcd for C_21_H_16_Cl_2_NaO_3_S [M+Na]^+^ 441.0089, found: 441.0094.

**1-Phenyl-3-(phenylsulfonyl)-2-*p*-tolylpropan-1-one (2k)** and **2-Phenyl-3-(phenylsulfonyl)-1-*p*-tolylpropan-1-one (2k′):** Yield = 81% (89 mg, **2k/2k′ =** 1.5/1). White solid. **^1^H NMR** (400 MHz, CDCl_3_) **2k** and its isomer **2k′**: *δ* = 7.93–7.77 (m, 4H), 7.60–7.54 (m, 1H), 7.52–7.35 (m, 4H), 7.24–7.02 (m, 5H), 5.27 (ddd, *J* = 9.1, 5.6, 3.9 Hz, 1H), 4.41 (ddd, *J* = 14.0, 8.6, 5.1 Hz, 1H), 3.45 (dt, *J* = 14.2, 3.8 Hz, 1.2H), 2.35 (s, 1H), 2.24 (s, 1.8H) ppm. **^13^C NMR** (100 MHz, CDCl_3_) **2k** and its isomer **2k′**: *δ* = 195.8, 195.3, 144.4, 139.4, 137.8, 136.6, 135.4, 133.6, 133.6, 133.3, 133.3, 132.8, 130.0, 129.3, 129.1, 129.0, 128.8, 128.5, 128.1, 128.0, 128.0, 127.9, 59.2, 59.1, 47.3, 47.1, 21.6, 21.0 ppm. **HRMS** m/z: calcd for C_22_H_20_NaO_3_S [M+Na]^+^ 387.1025, found: 387.1029.

**2-(3,4-Dimethylphenyl)-1-phenyl-3-(phenylsulfonyl)propan-1-one (2l)** and **1-(3,4-dimethylphenyl)-2-phenyl-3-(phenylsulfonyl)propan-1-one (2l′):** Yield = 78% (89 mg, **2l/2l′ =** 1.4/1). Colorless oil. **^1^H NMR** (400 MHz, CDCl_3_) **2l** and its isomer **2l′**: *δ* = 7.92–7.80 (m, 3H), 7.70–7.63 (m, 1H), 7.55–7.37 (m, 4H), 7.24–7.08 (m, 3H), 7.02–6.87 (m, 2H), 5.32–5.19 (m, 1H), 4.43–4.15 (m, 1H), 3.45 (ddd, *J* = 14.2, 5.7, 3.9 Hz, 1H), 2.26 (s, 2H), 2.14 (s, 3H) ppm. **^13^C NMR** (100 MHz, CDCl_3_) **2l** and its isomer **2l′**: *δ* = 195.8, 195.6, 143.2, 139.5, 139.4, 137.7, 137.0, 136.7, 136.5, 135.5, 134.2, 133.6, 133.5, 133.3, 133.2, 130.5, 130.4, 130.3, 130.0, 129.8, 129.3, 129.1, 129.1, 129.0, 128.9, 128.8, 128.5, 128.4, 128.1, 128.0, 127.8, 127.6, 126.6, 126.5, 125.6, 59.2, 59.1, 47.2, 47.1, 20.0, 19.8, 19.7, 19.7, 19.4, 19.3 ppm. **HRMS** m/z: calcd for C_23_H_22_Na O_3_S [M+Na]^+^ 401.1182, found: 401.1191.

**2-(Biphenyl-4-yl)-1-phenyl-3-(phenylsulfonyl)propan-1-one (2m)** and **1-(biphenyl-4-yl)-2-phenyl-3-(phenylsulfonyl)propan-1-one (2m′):** Yield = 72% (92 mg, **2m/2m′ =** 2/1). White solid. **^1^H NMR** (400 MHz, CDCl_3_) **2m** and its isomer **2m′**: *δ* = 7.92 (dd, *J* = 17.3, 7.9 Hz, 2H), 7.83–7.75 (m, 2H), 7.59–7.49 (m, 4H), 7.38 (ddt, *J* = 15.3, 10.0, 7.4 Hz, 7H), 7.26–7.14 (m, 4H), 5.30 (dt, *J* = 8.8, 4.5 Hz, 1H), 4.43–4.20 (m, 1H), 3.46 (ddd, *J* = 23.7, 14.2, 4.0 Hz, 1H) ppm. **^13^C NMR** (100 MHz, CDCl_3_) **2m** and its isomer **2m′**: *δ* = 195.7, 195.3, 146.1, 140.9, 140.1, 139.6, 139.4, 136.4, 135.4, 135.1, 134.0, 133.7, 133.6, 133.5, 129.5, 129.4, 129.2, 129.1, 128.9, 128.9, 128.8, 128.6, 128.6, 128.3, 128.1, 128.0, 127.5, 127.2, 127.2, 126.9, 59.2, 59.1, 47.5, 47.2 ppm. **HRMS** m/z: calcd for C_27_H_22_NaO_3_S [M+Na]^+^ 449.1182, found: 449.1186.

**1-(4-Bromophenyl)-2-phenyl-3-(phenylsulfonyl)propan-1-one (2n)** and **2-(4-bromophenyl)-1-phenyl-3-(phenylsulfonyl)propan-1-one (2n′):** Yield = 84% (108 mg, **2n/2n′ =** 1/1). White solid. **^1^H NMR** (400 MHz, CDCl_3_) **2n** and its isomer **2n′**: *δ* = 7.91–7.74 (m, 4H), 7.59–7.35 (m, 7H), 7.24–7.09 (m, 3H), 5.26 (ddd, *J* = 17.8, 8.5, 4.2 Hz, 1H), 4.45–4.23 (m, 1H), 3.45 (ddd, *J* = 14.1, 12.6, 4.2 Hz, 1H) ppm. **^13^C NMR** (100 MHz, CDCl_3_) **2n** and its isomer **2n′**: *δ* = 195.5, 194.9, 139.3, 139.3, 136.0, 135.3, 135.1, 134.1, 133.7, 133.7, 133.7, 132.5, 132.0, 131.9, 130.8, 130.3, 129.8, 129.5, 129.4, 129.2, 129.2, 128.8, 128.7, 128.7, 128.3, 128.2, 128.0, 128.0, 127.9, 122.2, 59.0, 58.9, 47.5, 46.8 ppm. **HRMS** m/z: calcd for C_21_H_17_^79^BrNaO_3_S [M+Na]^+^ 450.9974, found: 450.9979.

**2-Phenyl-3-(phenylsulfonyl)-1-(3-(trifluoromethyl)phenyl)propan-1-one (2o)** and **1-phenyl-3-(phenylsulfonyl)-2-(3-(trifluoromethyl)phenyl)propan-1-one (2o′):** Yield = 43% (54 mg, **2o/2o′ =** 2.4/1). Colorless oil. **^1^H NMR** (400 MHz, CDCl_3_) **2o** and its isomer **2o′**: *δ* = 8.18–8.11 (m, 1H), 7.95–7.79 (m, 3H), 7.65–7.40 (m, 7H), 7.32–7.27 (m, 3H), 5.37 (ddd, *J* = 43.8, 8.6, 4.1 Hz, 1H), 4.55–4.30 (m, 1H), 3.53 (ddd, *J* = 19.9, 14.2, 4.1 Hz, 1H) ppm. **^13^C NMR** (100 MHz, CDCl_3_) **2o** and its isomer **2o′**: *δ* = 195.4, 194.6, 139.3, 139.2, 138.0, 137.3, 137.2, 136.0, 136.0, 135.6, 133.8, 131.9, 131.7 (m), 131.5 (m), 131.2, 131.1, 130.8, 129.8, 129.8, 129.8, 129.6, 129.3, 129.3, 129.2, 128.8, 128.8, 128.1, 128.0, 127.9, 125.7 (m), 124.9 (m), 59.1, 59.0, 47.7, 47.1 ppm. **HRMS** m/z: calcd for C_22_H_17_F_3_NaO_3_S [M+Na]^+^ 441.0743, found: 441.0750.

**(*E*)-1,1-Diphenyl-3-(trifluoromethylsulfonyl)prop-2-en-1-ol (4a):** Yield = 80% (82 mg). White solid. M.p. 91–92 °C. **^1^H NMR** (400 MHz, CDCl_3_): *δ* = 7.79 (d, *J* = 14.8 Hz, 1H), 7.42–7.35 (m, 6H), 7.31 (dd, *J* = 7.7, 1.9 Hz, 4H), 6.92 (d, *J* = 14.7 Hz, 1H), 2.59 (s, 1H) ppm. **^13^C NMR** (100 MHz, CDCl_3_): *δ* = 161.6, 142.1, 129.0, 128.8, 126.7, 119.8, 119.4 (q, *J* = 322.7 Hz), 79.6 ppm. **^19^F NMR** (376 MHz, CDCl_3_): *δ* = −78.58 (s, 3F) ppm. **HRMS** m/z: calcd for C_16_H_13_F_3_NaO_3_S [M+Na]^+^ 365.0430, found: 365.0420.

**(*E*)-1,1-Dip-tolyl-3-(trifluoromethylsulfonyl)prop-2-en-1-ol (4b):** Yield = 63% (70 mg). White solid. M.p. 74–75 °C. **^1^H NMR** (400 MHz, Chloroform-*d*): *δ* = 7.75 (d, *J* = 14.7 Hz, 1H), 7.18 (s, 8H), 6.89 (d, *J* = 15.7 Hz, 1H), 2.37 (s, 6H) ppm. **^13^C NMR** (100 MHz, CDCl_3_): *δ* = 162.0, 139.3, 138.6, 129.6, 126.6, 119.6 (q, *J* = 323.2 Hz), 119.3, 79.3, 21.1 ppm. **^19^F NMR** (376 MHz, CDCl_3_): *δ* = −78.66 (s, 3F) ppm. **HRMS** m/z: calcd for C_18_H_17_F_3_NaO_3_S [M+Na]^+^ 393.0743, found: 393.0738.

**(*E*)-1,1-Bis(4-bromophenyl)-3-(trifluoromethylsulfonyl)prop-2-en-1-ol (4c):** Yield = 66% (99 mg). White solid. M.p. 123–124 °C. **^1^H NMR** (400 MHz, CDCl_3_): *δ* = 7.67 (d, *J* = 14.7 Hz, 1H), 7.57–7.49 (m, 4H), 7.21–7.13 (m, 3H), 6.91 (dd, *J* = 14.7, 1.0 Hz, 1H), 2.54 (s, 1H) ppm. **^13^C NMR** (100 MHz, CDCl_3_): *δ* = 160.0, 140.7, 132.3, 128.4, 123.4, 120.8, 119.6 (q, *J* = 325.5 Hz), 78.9 ppm. **^19^F NMR** (376 MHz, CDCl_3_): *δ* = −78.37 (s, 3F) ppm. **HRMS** m/z: calcd for C_16_H_11_^79^Br_2_F_3_NaO_3_S [M+Na]^+^ 522.8619, found: 522.8619.

**(*E*)-1,1-Bis(4-chlorophenyl)-3-(trifluoromethylsulfonyl)prop-2-en-1-ol (4d):** Yield = 59% (73 mg). White solid. M.p. 123–124 °C. **^1^H NMR** (400 MHz, CDCl_3_): *δ* = 7.68 (d, *J* = 14.7 Hz, 1H), 7.40–7.35 (m, 4H), 7.25–7.19 (m, 4H), 6.91 (d, *J* = 14.8 Hz, 1H), 2.55 (s, 1H) ppm. **^13^C NMR** (100 MHz, CDCl_3_): *δ* = 160.2, 140.2, 135.2, 129.3, 128.1, 120.7, 119.5 (q, *J* = 321.8 Hz), 78.8 ppm. **^19^F NMR** (376 MHz, CDCl_3_): *δ* = −78.49 (s, 3F) ppm. **HRMS** m/z: calcd for C_16_H_11_Cl_2_F_3_NaO_3_S [M+Na]^+^ 432.9650, found: 432.9645.

**(*E*)-1,1-Bis(4-fluorophenyl)-3-(trifluoromethylsulfonyl)prop-2-en-1-ol (4e):** Yield = 70% (79 mg). White solid. M.p. 105–106 °C. **^1^H NMR** (400 MHz, CDCl_3_): *δ* = 7.87 (d, *J* = 14.7 Hz, 1H), 7.45 (dd, *J* = 8.7, 5.3 Hz, 4H), 7.26 (d, *J* = 17.1 Hz, 4H), 7.08 (d, *J* = 14.8 Hz, 1H), 2.72 (s, 1H) ppm. **^13^C NMR** (100 MHz, CDCl_3_): *δ* = 162.6 (d, *J* = 247.9 Hz), 160.8, 137.8 (d, *J* = 3.2 Hz), 128.7 (d, *J* = 8.3 Hz), 120.2, 119.3 (q, *J* = 324.7 Hz), 116.0 (d, *J* = 21.7 Hz), 78.7 ppm. **^19^F NMR** (376 MHz, CDCl_3_): *δ* = −78.44 (s, 3F), −112.07 (s, 2F) ppm. **HRMS** m/z: calcd for C_16_H_11_F_5_NaO_3_S [M+H]^+^ 401.0241, found: 401.0234.

**(*E*)-1-Phenyl-1-*p*-tolyl-3-(trifluoromethylsulfonyl)prop-2-en-1-ol (4f):** Yield = 67% (72 mg). White solid. M.p. 63–64 °C. **^1^H NMR** (400 MHz, CDCl_3_): *δ* = 7.78 (dd, *J* = 15.8, 14.8 Hz, 1H), 7.47–7.23 (m, 7H), 7.16 (dd, *J* = 39.2, 2.1 Hz, 2H), 6.93 (dd, *J* = 14.8, 4.2 Hz, 1H), 2.60 (s, 1H), 2.40 (d, *J* = 8.3 Hz, 3H) ppm. **^13^C NMR** (100 MHz, CDCl_3_): *δ* = 161.8, 161.0, 142.2, 141.8, 141.2, 139.2, 138.7, 136.8, 135.0, 131.3, 129.6, 129.1, 129.0, 128.9, 128.6, 127.3, 126.7, 126.7, 125.0, 120.1, 119.5, 119.4 (q, *J* = 324.3 Hz), 119.4 (q, *J* = 323.8 Hz), 79.4, 79.0, 21.0, 19.7 ppm. **^19^F NMR** (376 MHz, CDCl_3_): *δ* = −78.54 (s, 1.5F), −78.63 (s, 1.5F) ppm. **HRMS** m/z: calcd for C_17_H_15_F_3_NaO_3_S [M+Na]^+^ 379.0586, found: 379.0577.

**(*E*)-1-(Biphenyl-4-yl)-1-phenyl-3-(trifluoromethylsulfonyl)prop-2-en-1-ol (4g):** Yield = 77% (97 mg). White solid. M.p. 122–123 °C. **^1^H NMR** (400 MHz, CDCl_3_): *δ* = 7.83 (d, *J* = 14.7 Hz, 1H), 7.64–7.58 (m, 3H), 7.53–7.32 (m, 11H), 6.96 (d, *J* = 14.8 Hz, 1H), 2.59 (s, 1H) ppm. **^13^C NMR** (100 MHz, CDCl_3_): *δ* = 161.5, 142.1, 141.7, 141.0, 140.0, 129.0, 128.9, 128.8, 127.8, 127.6, 127.2, 127.1, 126.8, 119.9, 119.4 (q, *J* = 324.3 Hz), 79.5 ppm. **^19^F NMR** (376 MHz, CDCl_3_): *δ* = −78.59 (s, 3F) ppm. **HRMS** m/z: calcd for C_22_H_17_F_3_NaO_3_S [M+Na]^+^ 441.0743, found: 441.0752.

**(*E*)-1-(4-Bromophenyl)-1-phenyl-3-(trifluoromethylsulfonyl)prop-2-en-1-ol (4h):** Yield = 72% (91 mg). White solid. M.p. 110–111 °C. **^1^H NMR** (400 MHz, CDCl_3_): *δ* = 7.73 (d, *J* = 14.7 Hz, 1H), 7.56–7.49 (m, 2H), 7.42–7.37 (m, 3H), 7.29–7.26 (m, 2H), 7.23–7.18 (m, 2H), 6.92 (dd, *J* = 14.7, 1.0 Hz, 1H), 2.55 (s, 1H) ppm. **^13^C NMR** (100 MHz, CDCl_3_): *δ* = 160.8, 141.8, 141.0, 132.1, 129.2, 129.1, 128.4, 126.7, 123.0, 120.3, 119.4 (q, *J* = 323.3 Hz), 79.2 ppm. **^19^F NMR** (376 MHz, CDCl_3_): *δ* = −78.36 (s, 3F) ppm. **HRMS** m/z: calcd for C_16_H_12_^81^BrF_3_NaO_3_S [M+Na]^+^ 444.9535, found: 444.9512.

**(*E*)-1-(2-Fluorophenyl)-1-phenyl-3-(trifluoromethylsulfonyl)prop-2-en-1-ol (4i):** Yield = 71% (77 mg). White solid. M.p. 63–64 °C. **^1^H NMR** (400 MHz, CDCl_3_): *δ* = 7.87 (d, *J* = 14.8 Hz, 1H), 7.54 (td, *J* = 7.9, 1.8 Hz, 1H), 7.44–7.36 (m, 4H), 7.25 (ddd, *J* = 8.1, 5.5, 1.6 Hz, 3H), 7.09 (ddd, *J* = 11.6, 8.2, 1.2 Hz, 1H), 6.99–6.92 (m, 1H) ppm.

**^13^C NMR** (100 MHz, CDCl_3_): *δ* = 159.5 (d, *J* = 246.0 Hz), 158.5 (d, *J* = 3.0 Hz), 141.6, 130.9 (d, *J* = 8.6 Hz), 129.2, 129.0, 128.9 (d, *J* = 11.2 Hz), 127.9 (d, *J* = 3.0 Hz), 125.9, 124.8 (d, *J* = 3.4 Hz), 120.7, 119.5 (q, *J* = 324.6 Hz), 116.6 (d, *J* = 21.9 Hz), 77.7 (d, *J* = 1.3 Hz) ppm. **^19^F NMR** (376 MHz, CDCl_3_): *δ* = −78.6 (s, 3F), −110.1 (s, 1F) ppm. **HRMS** m/z: calcd for C_16_H_12_F_4_NaO_3_S [M+Na]^+^ 383.0335, found: 383.0337.

**1,2-Diphenylprop-2-en-1-one (5):** Yield = 83% (52 mg). Colorless oil. **^1^H NMR** (400 MHz, CDCl_3_): *δ* = 7.84–7.79 (m, 2H), 7.47–7.41 (m, 1H), 7.35–7.30 (m, 4H), 7.26–7.20 (m, 3H), 5.97 (s, 1H), 5.54 (s, 1H) ppm. **^13^C NMR** (100 MHz, CDCl_3_): *δ* = 197.6, 148.1, 137.0, 136.9, 133.1, 129.9, 128.6, 128.4, 128.4, 127.0, 121.0 ppm. **HRMS** m/z: calcd for C_15_H_13_O [M+H]^+^ 209.0961, found: 209.0966.

## 4. Conclusions

In summary, we have developed an efficient and metal-free oxidative functionalization of allylic sulfones/allylic triflones derivatives for the facile construction of β-sulfonyl ketones and triflyl allylic alcohols in moderate to good yields. The success of this approach could be attributed to the combination of hypervalent iodine reagent and strong acid (H_2_SO_4_). Moreover, water serves as an eco-friendly oxygen atom source. In addition, the reaction showed a wide substrate scope. The scale-up synthesis of potential pharmaceutically and biologically active sulfone compounds provides chemists an opportunity for further application.

## Data Availability

The original contributions presented in this study are included in the article/Supplementary Material. Further inquiries can be directed to the corresponding author(s).

## Supplementary Materials

The following supporting information can be downloaded at https://www.mdpi.com/article/10.3390/molecules31162794/s1. 1H and 13C NMR data of all synthesized compounds.
