# Supplementary material for: Metal-Free Oxidative Functionalization of Allylic Sulfones/Allylic Triflones with Water Participation Enabled by Hypervalent Iodine Reagents
Source: Molecules. 2026 Aug 11;31(16):2794. doi: 10.3390/molecules31162794 (PMC13515277; doi:10.3390/molecules31162794)
Supplement: Supplementary file 1 [file molecules-31-02794-s001.zip › molecules-4478102-supplementary.pdf]

Supporting Information

Metal-Free Oxidative Functionalization of Allylic Sulfones/Allylic Triflones with Water Participation Enabled by Hypervalent Iodine Reagents

Qian Tong, Li-Ting Xiao, Ming-Yang Gu, Xue-Qiang Chu * and Danhua Ge *

Technical Institute of Fluorochemistry, School of Chemistry and Molecular Engineering, Nanjing Tech University, Nanjing 211816, China
* Correspondence: xueqiangchu@njtech.edu.cn (X.-Q.C.); gedanhua@njtech.edu.cn (D.G.)

Table of Contents

1. General information page S2

2. General procedure for the synthesis of β-sulfonyl ketones **2** page S2

3. General procedure for the synthesis of triflyl allylic alcohols **4** page S2

4. General procedure for the scale-up synthesis of product **2a** page S2

5. General procedure for the scale-up synthesis of product **4a** page S3

6. Further transformation of product **2a** page S3

7. Mechanistic studies page S4

8. References page S6

9. ^{1}H, ^{19}F, ^{13}C NMR, and HRMS spectra of products page S7

1. General information

Unless otherwise stated, all reagents were purchased from commercial suppliers and used without further purification. All reactions were carried out under air atmosphere using undistilled solvent. Melting points were recorded on an Electrothermal digital melting point apparatus. IR spectra were recorded on a FT-IR spectrophotometer using KBr optics. ^{1}H, ^{19}F, and ^{13}C NMR spectra were recorded in $CDCl_3$ on Bruker Avance or Joel 400 MHz spectrometers. The chemical shifts (δ) are reported in ppm and coupling constants (J) in Hz. High resolution mass spectra (HRMS) were obtained using a commercial apparatus (ESI Source). Column chromatography was generally performed on silica gel (300-400 mesh) and reactions were monitored by thin layer chromatography (TLC) using UV light to visualize the course of the reactions.

2. General procedure for the synthesis of β-sulfonyl ketones 2

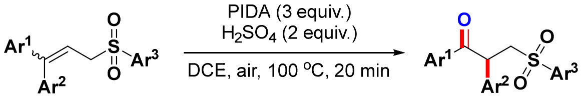

A solution of allylic sulfone **1** (0.3 mmol),[1] $PhI(OAc)_2$ (290 mg, 0.9 mmol, PIDA), and H_2SO_4 (60 mg, 0.6 mmol, wt% = 98%) in DCE (4 mL) was stirred at 100 °C under air for 20 min. Upon completion of the reaction (indicated by TLC), the reaction was then quenched by saturated $NaHCO_3$ solution (20 mL) and extracted with EtOAc (20 mL x 3). The organic layer was washed with saturated brine twice, dried over $MgSO_4$, filtered, and concentrated under reduced pressure. The crude product was purified by flash column chromatography (300-400 mesh) using petroleum ether/ethyl acetate (100/1 ~ 20/1) as eluent to afford the pure products **2**.

3. General procedure for the synthesis of triflyl allylic alcohols 4

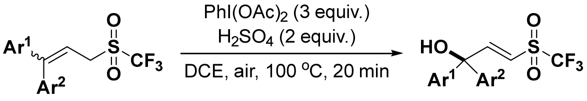

A solution of allylic triflone **3** (0.3 mmol), $PhI(OAc)_2$ (290 mg, 0.9 mmol, PIDA), and H_2SO_4 (60 mg, 0.6 mmol, wt% = 98%) in DCE (4 mL) was stirred at 100 °C under air for 20 min. Upon completion of the reaction (indicated by TLC), the reaction was then quenched by saturated $NaHCO_3$ solution (20 mL) and extracted with EtOAc (20 mL x 3). The organic layer was washed with saturated brine twice, dried over $MgSO_4$, filtered, and concentrated under reduced pressure. The crude product was purified by flash column chromatography (300-400 mesh) using petroleum ether/ethyl acetate (100/1 ~ 6/1) as eluent to afford the pure products **4**.

4. General procedure for the scale-up synthesis of product 2a

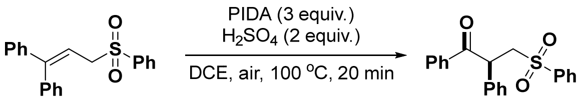

A solution of (3-(phenylsulfonyl)prop-1-ene-1,1-diyl)dibenzene (1672 mg, 5 mmol, **1a**), $PhI(OAc)_2$ (4832 mg, 15 mmol, PIDA), and H_2SO_4 (1000 mg, 10 mmol, wt% = 98%) in DCE (20

mL) was stirred at 100 °C under air for 20 min. Upon completion of the reaction (indicated by TLC), the reaction was then quenched by saturated $NaHCO_3$ solution (100 mL) and extracted with EtOAc (100 mL x 3). The organic layer was washed with saturated brine twice, dried over $MgSO_4$, filtered, and concentrated under reduced pressure. The crude product was purified by flash column chromatography (300-400 mesh) using petroleum ether/ethyl acetate (100/1 ~ 20/1) as eluent to afford the pure product **2a** (1384 mg, 79% yield).

5. General procedure for the scale-up synthesis of product 4a

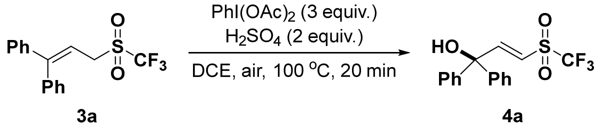

A solution of (3-((trifluoromethyl)sulfonyl)prop-1-ene-1,1-diyl)dibenzene (1632 mg, 5 mmol, **3a**), $PhI(OAc)_2$ (4832 mg, 15 mmol, PIDA), and H_2SO_4 (1000 mg, 10 mmol, wt% = 98%) in DCE (20 mL) was stirred at 100 °C under air for 20 min. Upon completion of the reaction (indicated by TLC), the reaction was then quenched by saturated $NaHCO_3$ solution (100 mL) and extracted with EtOAc (100 mL x 3). The organic layer was washed with saturated brine twice, dried over $MgSO_4$, filtered, and concentrated under reduced pressure. The crude product was purified by flash column chromatography (300-400 mesh) using petroleum ether/ethyl acetate (100/1 ~ 6/1) as eluent to afford the pure product **4a** (1113 mg, 65% yield).

6. Further transformation of product 2a

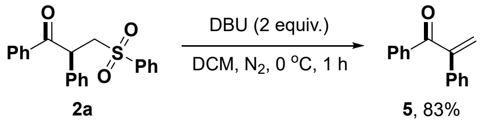

A solution of (3-(phenylsulfonyl)prop-1-ene-1,1-diyl)dibenzene (105 mg, 0.3 mmol, **1a**) and DBU (93 mg, 0.6 mmol) in DCM (2 mL) was stirred at 0 °C under N_2 for 1 hour. Upon completion of the reaction (indicated by TLC), the reaction was then quenched by saturated NaCl solution (20 mL) and extracted with EtOAc (20 mL x 3). The organic layer was washed with saturated brine twice, dried over $MgSO_4$, filtered, and concentrated under reduced pressure. The crude product was purified by flash column chromatography (300-400 mesh) using petroleum ether/ethyl acetate (100/1 ~ 6/1) as eluent to afford the pure product **5** (52 mg, 83% yield).

7. Mechanistic studies

1) Radical trapping experiments

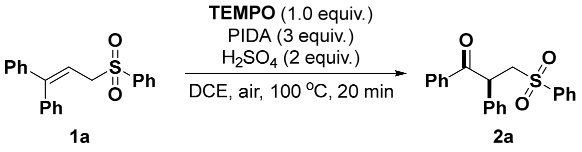

A solution of (3-(phenylsulfonyl)prop-1-ene-1,1-diyl)dibenzene (105 mg, 0.3 mmol, **1a**), $PhI(OAc)_2$ (290 mg, 0.9 mmol, PIDA), H_2SO_4 (60 mg, 0.6 mmol, wt% = 98%), and 2,2,6,6-tetramethylpiperidinooxy (47 mg, 0.3 mmol, TEMPO) in DCE (4 mL) was stirred at 100 °C under air for 20 min. Upon completion of the reaction (indicated by TLC), the reaction was then quenched by saturated $NaHCO_3$ solution (20 mL) and extracted with EtOAc (20 mL x 3). The organic layer was washed with saturated brine twice, dried over $MgSO_4$, filtered, and concentrated under reduced pressure. The crude product was purified by flash column chromatography (300-400 mesh) using petroleum ether/ethyl acetate (100/1 ~ 20/1) as eluent to afford the pure product **2a** (84 mg, 80% yield).

This result suggested that radical intermediate was not involved in the oxidative rearrangement reaction.

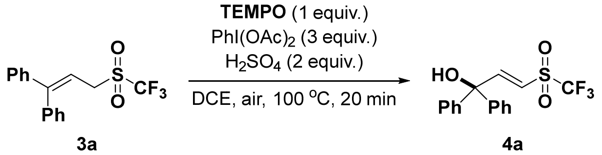

A solution of (3-((trifluoromethyl)sulfonyl)prop-1-ene-1,1-diyl)dibenzene (98 mg, 0.3 mmol, **3a**), $PhI(OAc)_2$ (290 mg, 0.9 mmol, PIDA), H_2SO_4 (60 mg, 0.6 mmol, wt% = 98%), and 2,2,6,6-tetramethylpiperidinooxy (47 mg, 0.3 mmol, TEMPO) in DCE (4 mL) was stirred at 100 °C under air for 20 min. Upon completion of the reaction (indicated by TLC), the reaction was then quenched by saturated $NaHCO_3$ solution (20 mL) and extracted with EtOAc (20 mL x 3). The organic layer was washed with saturated brine twice, dried over $MgSO_4$, filtered, and concentrated under reduced pressure. The crude product was purified by flash column chromatography (300-400 mesh) using petroleum ether/ethyl acetate (100/1 ~ 6/1) as eluent to afford the pure product **4a** (54 mg, 52% yield).

This result suggested that radical intermediate was not involved in the oxidative hydroxylation reaction.

2) ^{18}O-Labeling experiment

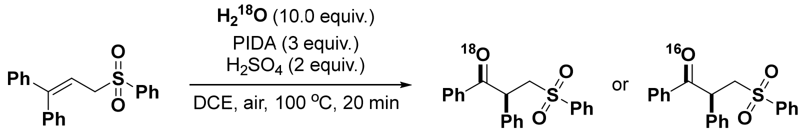

2a-^{18}O/2a = 1/2.3

A solution of (3-(phenylsulfonyl)prop-1-ene-1,1-diyl)dibenzene (105 mg, 0.3 mmol, **1a**), $PhI(OAc)_2$ (290 mg, 0.9 mmol, PIDA), H_2SO_4 (60 mg, 0.6 mmol, wt% = 98%), and $H_2{}^{18}O$ (60 mg, 3 mmol) in DCE (4 mL) was stirred at 100 °C under air for 20 min. Upon completion of the reaction, the reaction mixture was analysis by HPLC-MS.

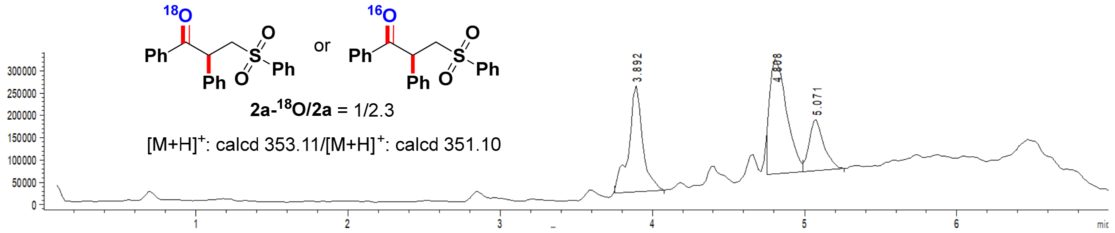

3) Detection of possible intermediate 6 and compound 7

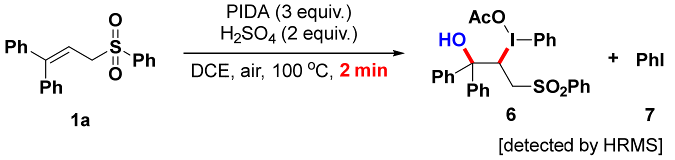

A solution of (3-(phenylsulfonyl)prop-1-ene-1,1-diyl)dibenzene (105 mg, 0.3 mmol, **1a**), PhI(OAc)$_2$ (290 mg, 0.9 mmol, PIDA), and H_2SO_4 (60 mg, 0.6 mmol, wt% = 98%) in DCE (4 mL) was stirred at 100 °C under air for 2 min. Upon completion of the reaction, the reaction mixture was analysis by HRMS.

<u>This result suggested that intermediates 6 and 7 might be involved in the reaction system.</u>

<u>Intermediate 6:</u>

Elemental Composition Report **Page 1**

Single Mass Analysis
Tolerance = 5.0 mDa / DBE: min = -1.5, max = 50.0
Element prediction: Off
Number of isotope peaks used for i-FIT = 3

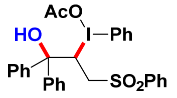

[M+H]$^+$ = $C_{29}H_{28}IO_5S^+$
calcd: 615.0697
found: 615.0702

Monoisotopic Mass, Even Electron Ions
10 formula(e) evaluated with 1 results within limits (up to 50 best isotopic matches for each mass)
Elements Used:
C: 28-30 H: 27-29 O: 4-5 S: 1-2 Si: 0-1 I: 0-1

wyw-x (0.027) Is (1.00,1.00) C29H27IO5S
1: TOF MS ES+

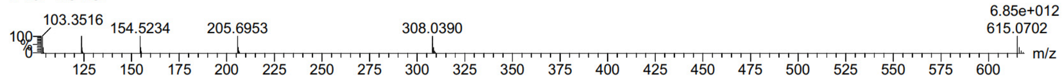

Minimum:				-1.5				
Maximum:		5.0	10.0	50.0				
Mass	Calc. Mass	mDa	PPM	DBE	i-FIT	Norm	Conf(%)	Formula
615.0702	615.0702	0.0	0.0	15.5	49.7	n/a	n/a	C29 H28 O5 S I

<u>Compound 7:</u>

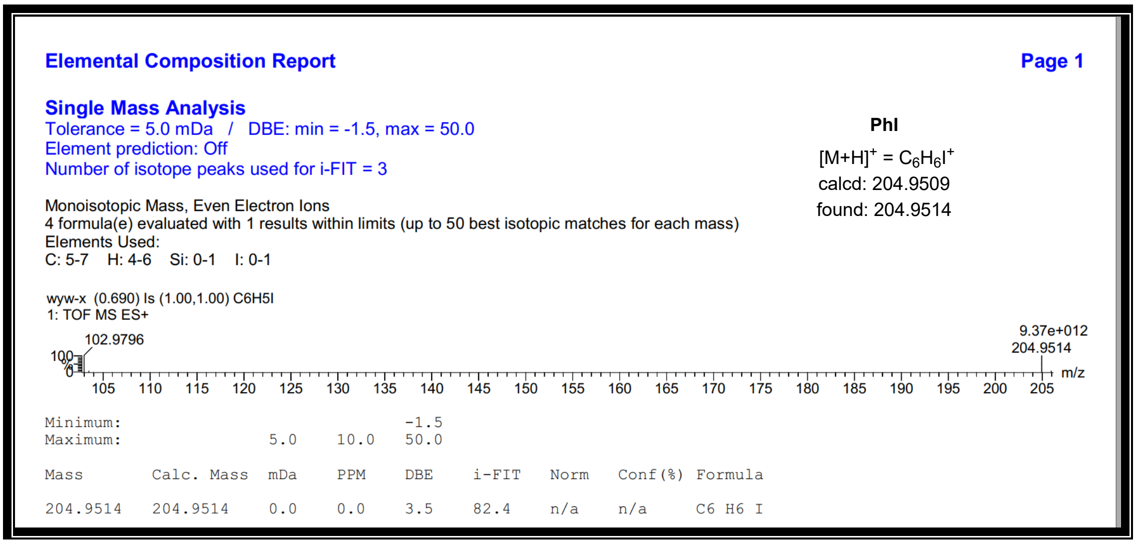

Elemental Composition Report Page 1

Single Mass Analysis
Tolerance = 5.0 mDa / DBE: min = -1.5, max = 50.0
Element prediction: Off
Number of isotope peaks used for i-FIT = 3

Monoisotopic Mass, Even Electron Ions
4 formula(e) evaluated with 1 results within limits (up to 50 best isotopic matches for each mass)
Elements Used:
C: 5-7 H: 4-6 Si: 0-1 I: 0-1

PhI
$[M+H]^+ = C_6H_6I^+$
calcd: 204.9509
found: 204.9514

wyw-x (0.690) Is (1.00,1.00) C6H5I
1: TOF MS ES+

Minimum:				-1.5				
Maximum:		5.0	10.0	50.0				
Mass	Calc. Mass	mDa	PPM	DBE	i-FIT	Norm	Conf(%)	Formula
204.9514	204.9514	0.0	0.0	3.5	82.4	n/a	n/a	C6 H6 I

8. References

[1] X.-Q. Chu, H. Meng, X.-P. Xu and S.-J. Ji, One-Pot Synthesis of Allylic Sulfones, Ketosulfones, and Triflyl Allylic Alcohols from Domino Reactions of Allylic Alcohols with Sulfinic Acid under Metal-Free Conditions. *Chem. Eur. J.* 2015, **21**, 11359-11368.

9. ^{1}H, ^{19}F, ^{13}C NMR, and HRMS spectra of products

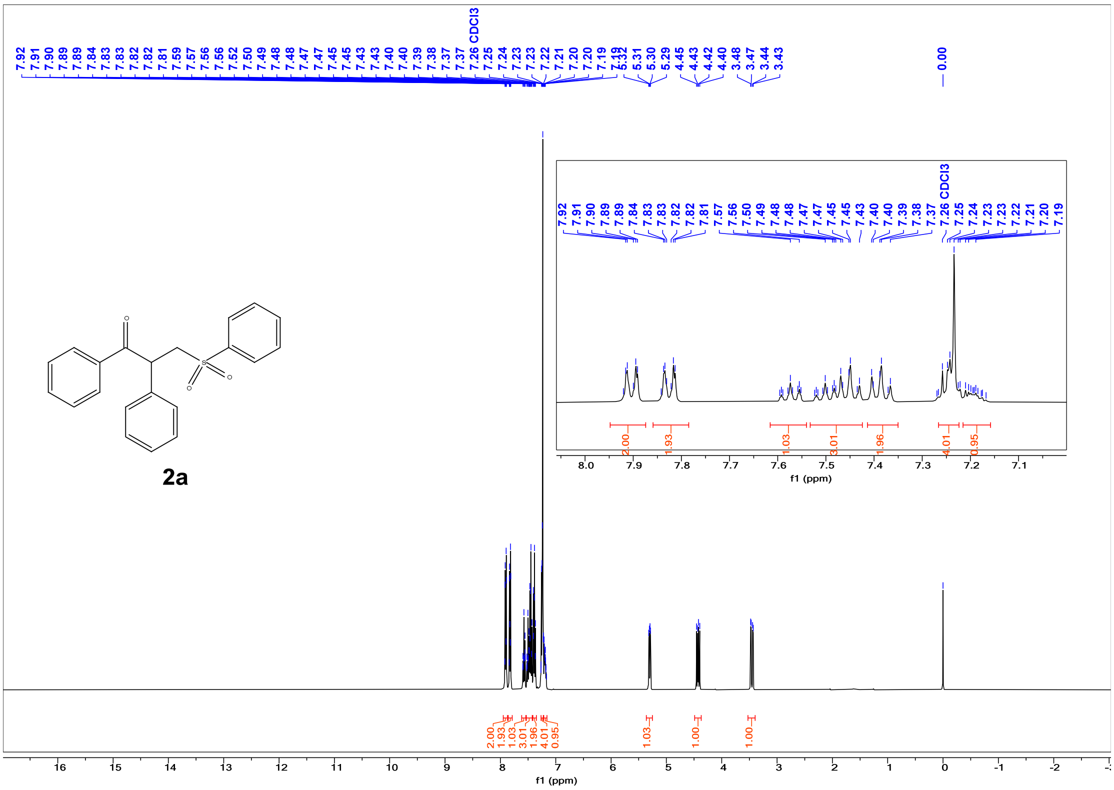

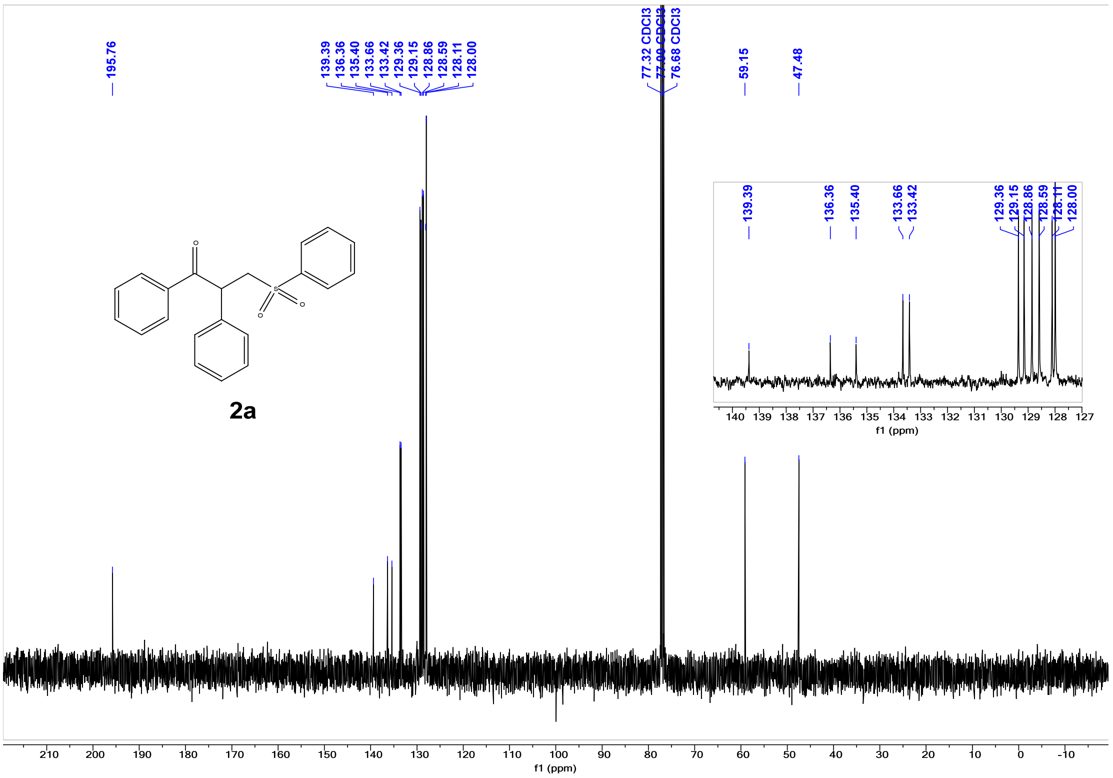

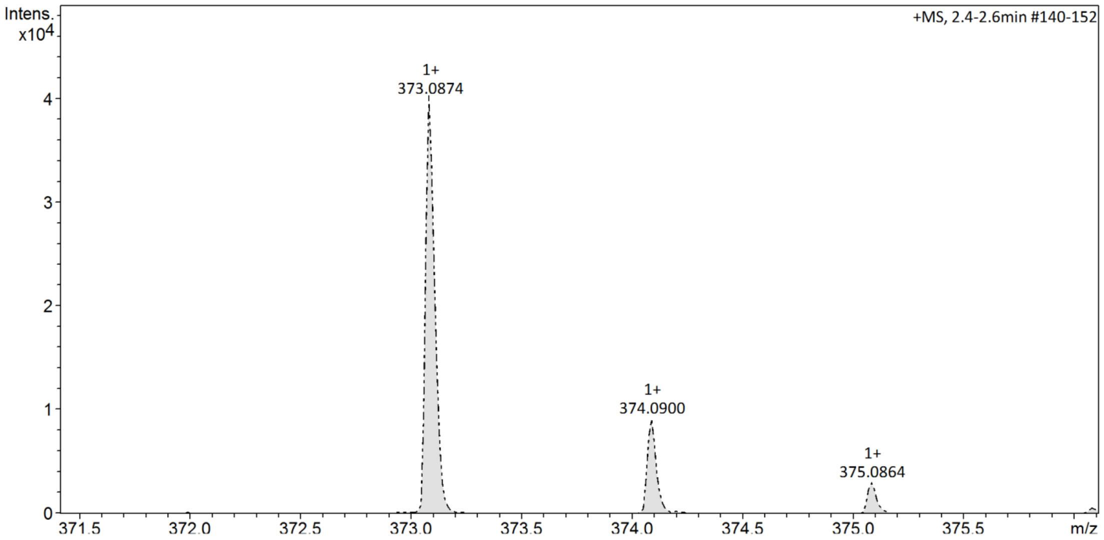
Intens.
x10^4
+MS, 2.4-2.6min #140-152
1+
373.0874
1+
374.0900
1+
375.0864
4
3
2
1
0
371.5
372.0
372.5
373.0
373.5
374.0
374.5
375.0
375.5
m/z

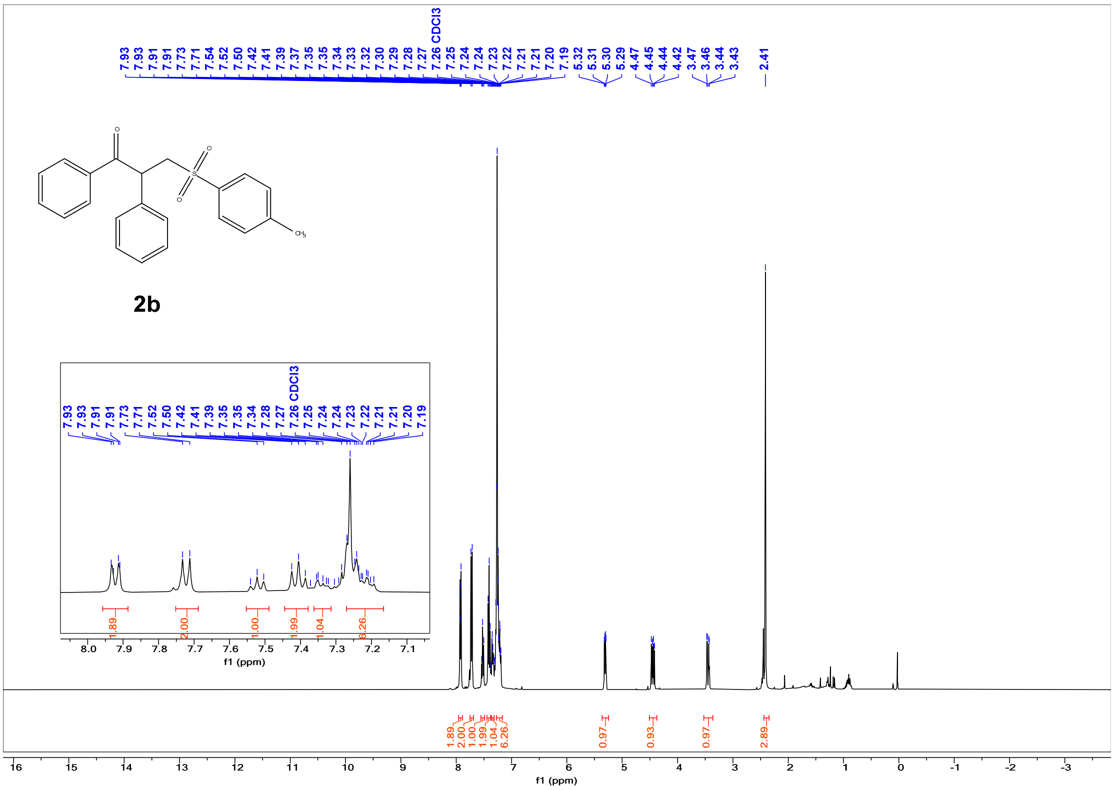
7.93 7.93 7.91 7.91 7.73 7.71 7.54 7.52 7.50 7.42 7.41 7.39 7.37 7.35 7.35 7.34 7.33 7.32 7.30 7.29 7.28 7.27 7.26 CDCl3 7.25 7.24 7.24 7.23 7.22 7.21 7.21 7.20 7.19 5.32 5.31 5.30 5.29 4.47 4.45 4.44 4.42 3.47 3.46 3.44 3.43 2.41
2b
1.89 2.00 1.00 1.99 1.04 6.26 0.97 0.93 0.97 2.89
f1 (ppm)

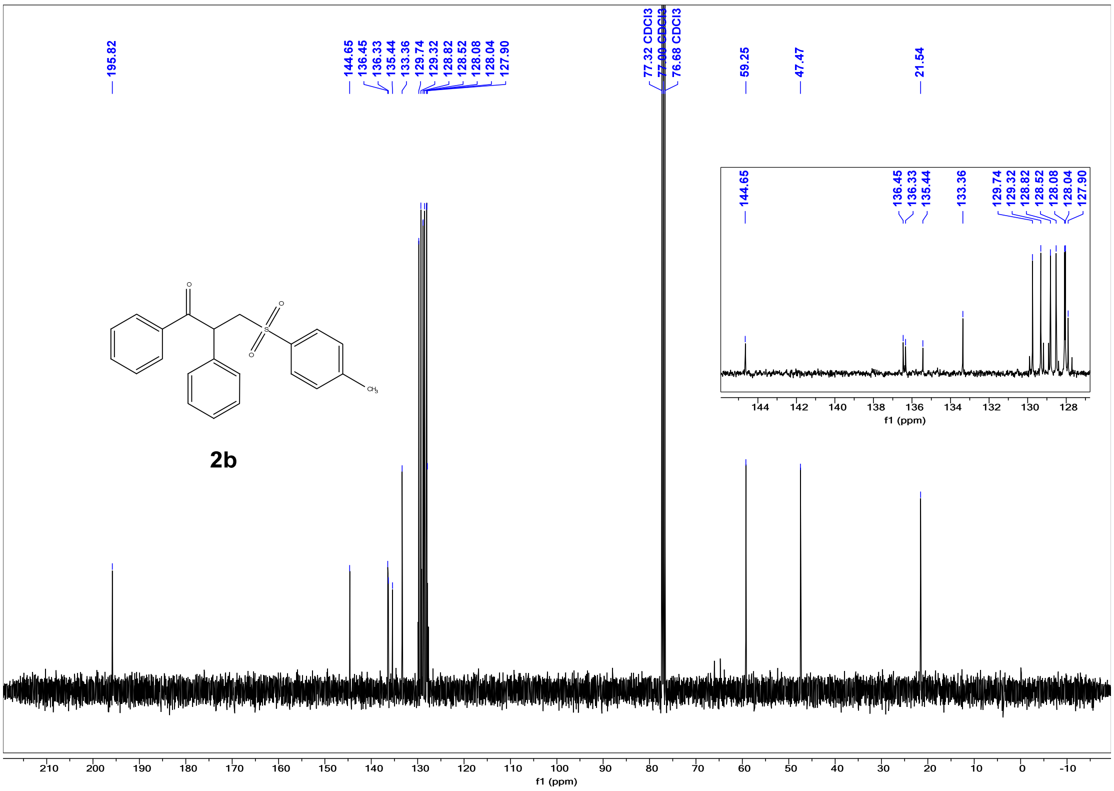
195.82
144.65 136.45 136.33 135.44 133.36 129.74 129.32 128.82 128.52 128.08 128.04 127.90
77.32 CDCl3 77.00 CDCl3 76.68 CDCl3
59.25
47.47
21.54
2b
f1 (ppm)

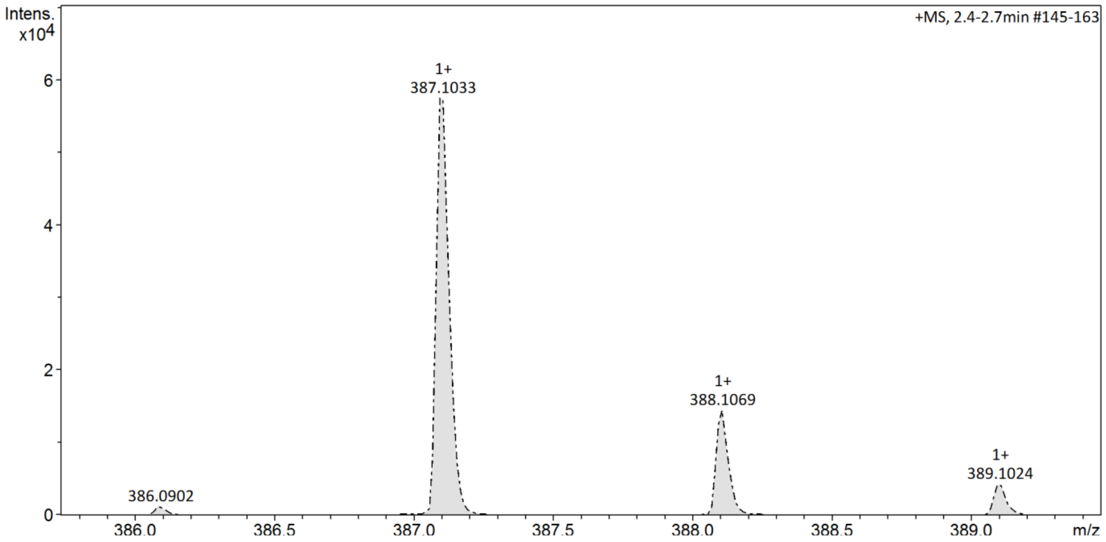
Intens.
x10^4
+MS, 2.4-2.7min #145-163
1+
387.1033
1+
388.1069
1+
389.1024
386.0902
6
4
2
0
386.0
386.5
387.0
387.5
388.0
388.5
389.0
m/z

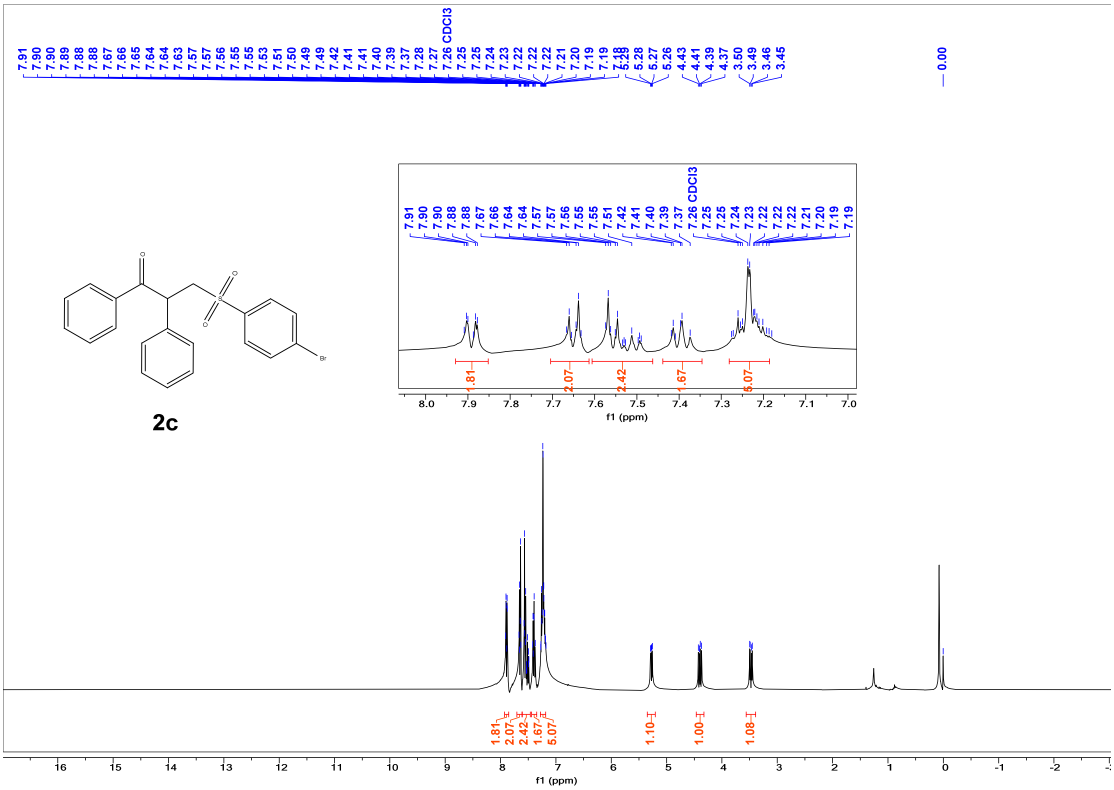
7.91 7.90 7.90 7.89 7.88 7.88 7.67 7.66 7.65 7.64 7.64 7.63 7.57 7.57 7.56 7.55 7.55 7.53 7.51 7.50 7.49 7.49 7.42 7.41 7.41 7.40 7.39 7.37 7.28 7.27 7.26 CDCl3 7.25 7.25 7.24 7.23 7.22 7.22 7.22 7.21 7.20 7.19 7.19
5.28 5.28 5.27 5.26
4.43 4.41 4.39 4.37
3.50 3.49 3.46 3.45
0.00
1.81 2.07 2.42 1.67 5.07 1.10 1.00 1.08
f1 (ppm)
Br
2c

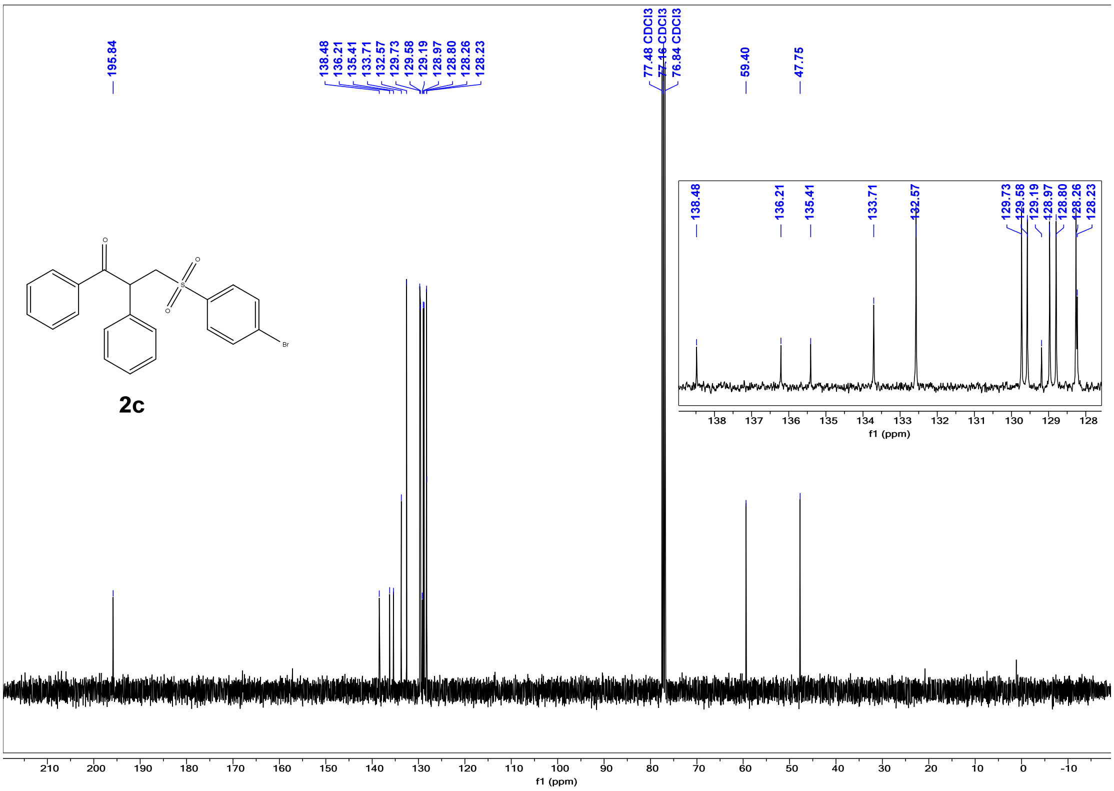
195.84
138.48 136.21 135.41 133.71 132.57 129.73 129.58 129.19 128.97 128.80 128.26 128.23
77.48 CDCl3 77.16 CDCl3 76.84 CDCl3
59.40
47.75
f1 (ppm)
Br
2c

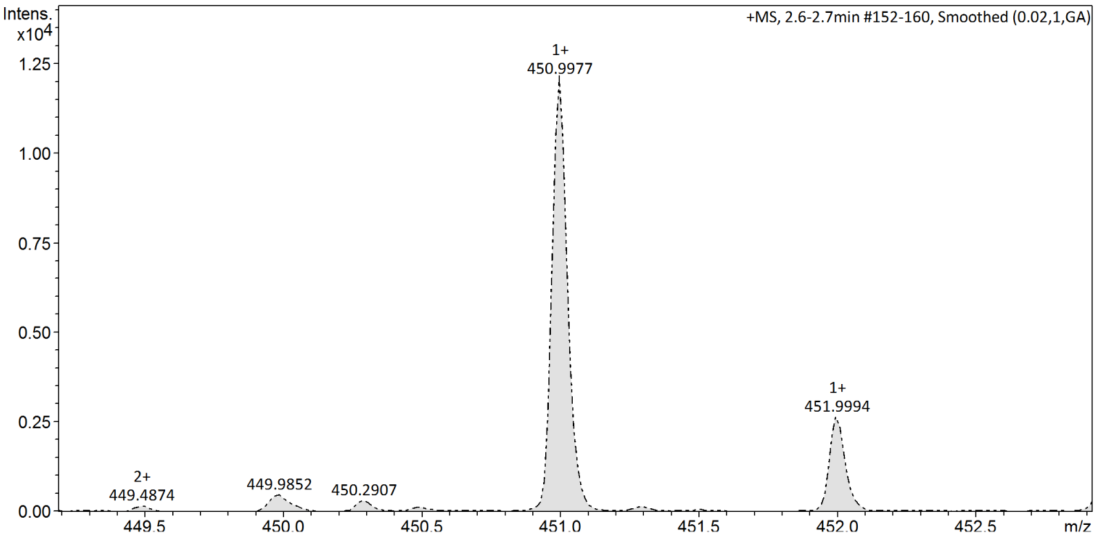

Intens.
x10^4
1.25
1.00
0.75
0.50
0.25
0.00
+MS, 2.6-2.7min #152-160, Smoothed (0.02,1,GA)
1+
450.9977
1+
451.9994
2+
449.4874
449.9852
450.2907
449.5
450.0
450.5
451.0
451.5
452.0
452.5
m/z

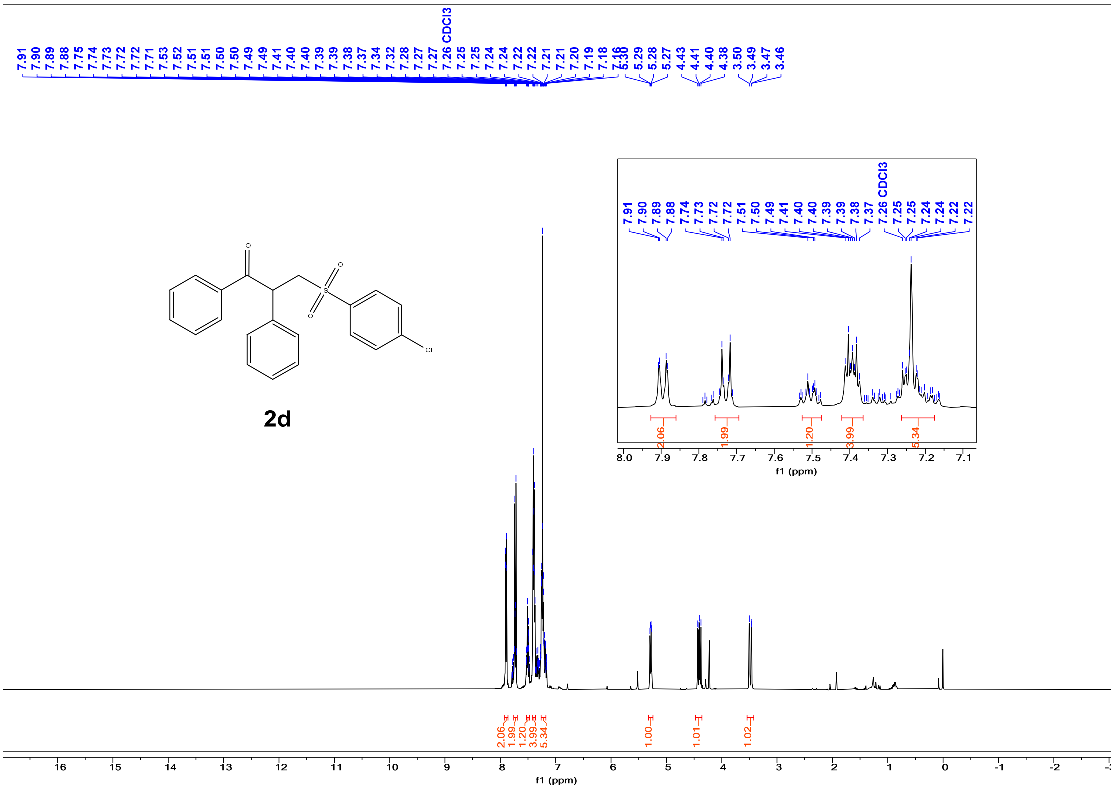

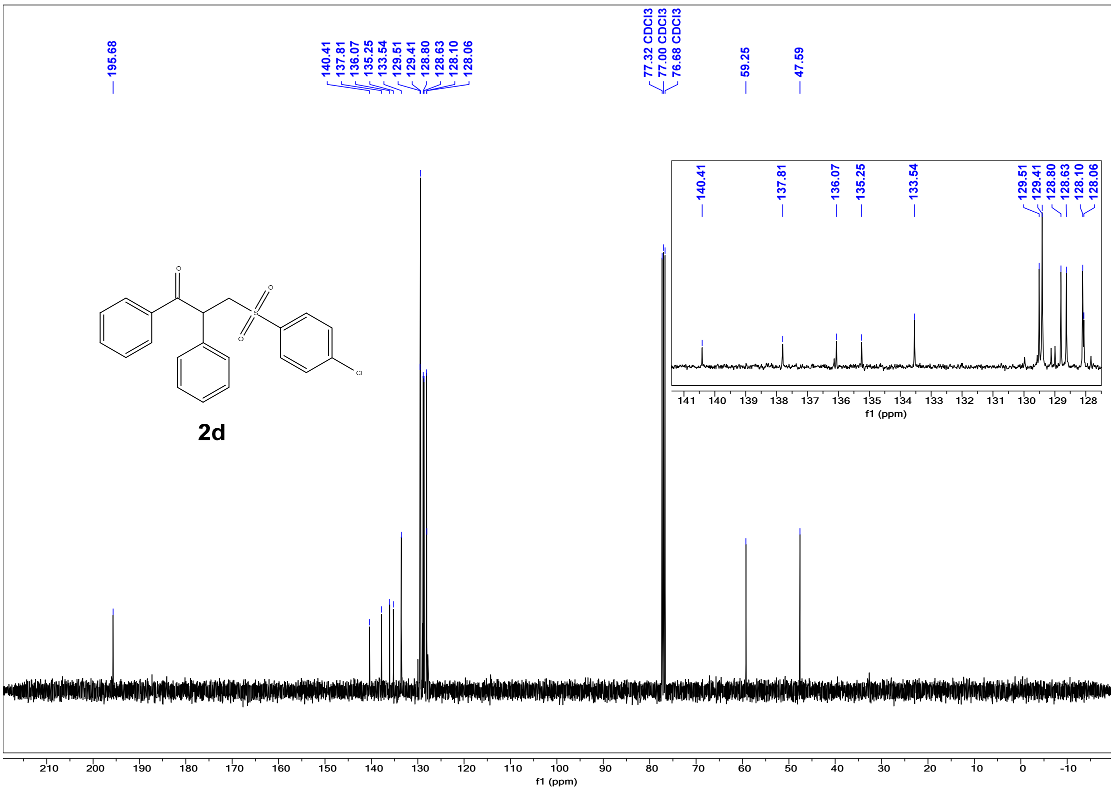

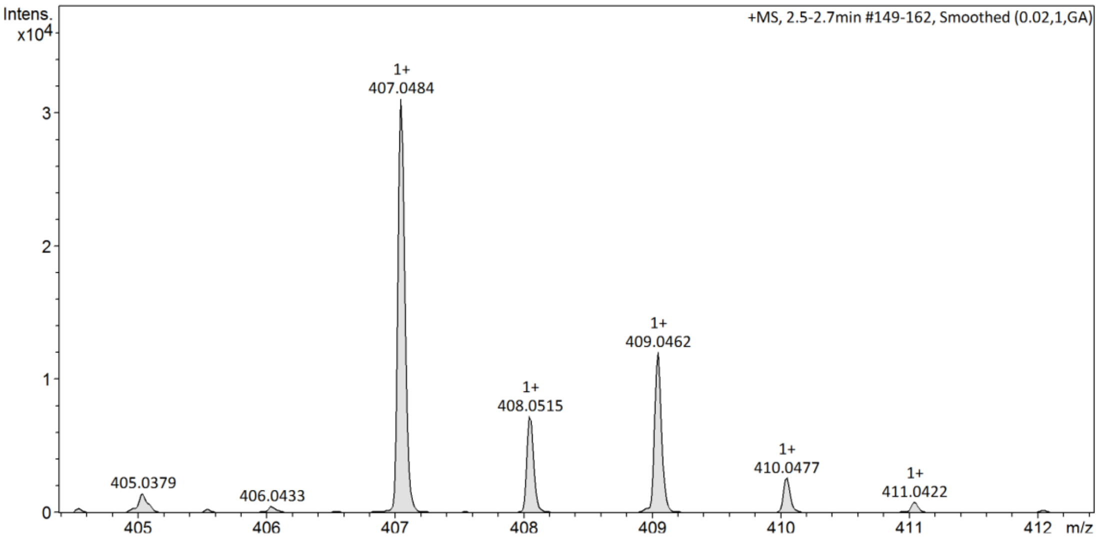
Intens.
x10^4
+MS, 2.5-2.7min #149-162, Smoothed (0.02,1,GA)
1+
407.0484
1+
409.0462
1+
408.0515
1+
410.0477
1+
411.0422
405.0379
406.0433
3
2
1
0
405
406
407
408
409
410
411
412
m/z

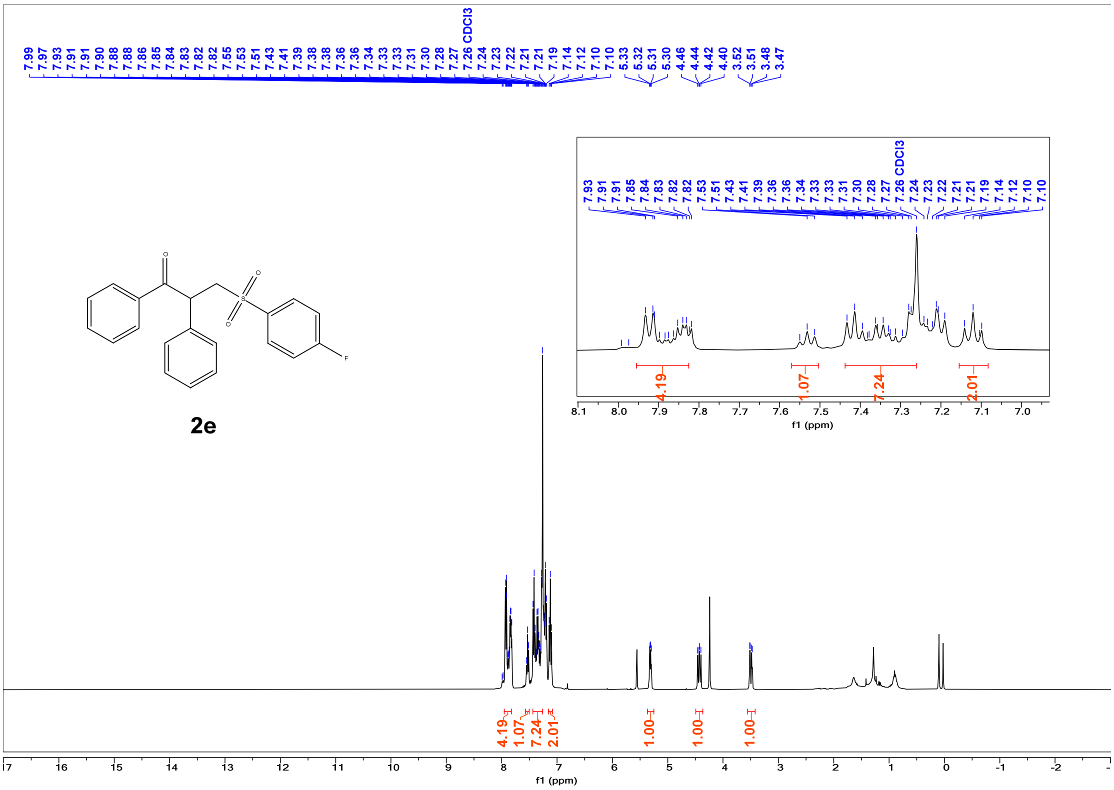
2e
f1 (ppm)
CDCl3
4.19
1.07
7.24
2.01
1.00
1.00
1.00

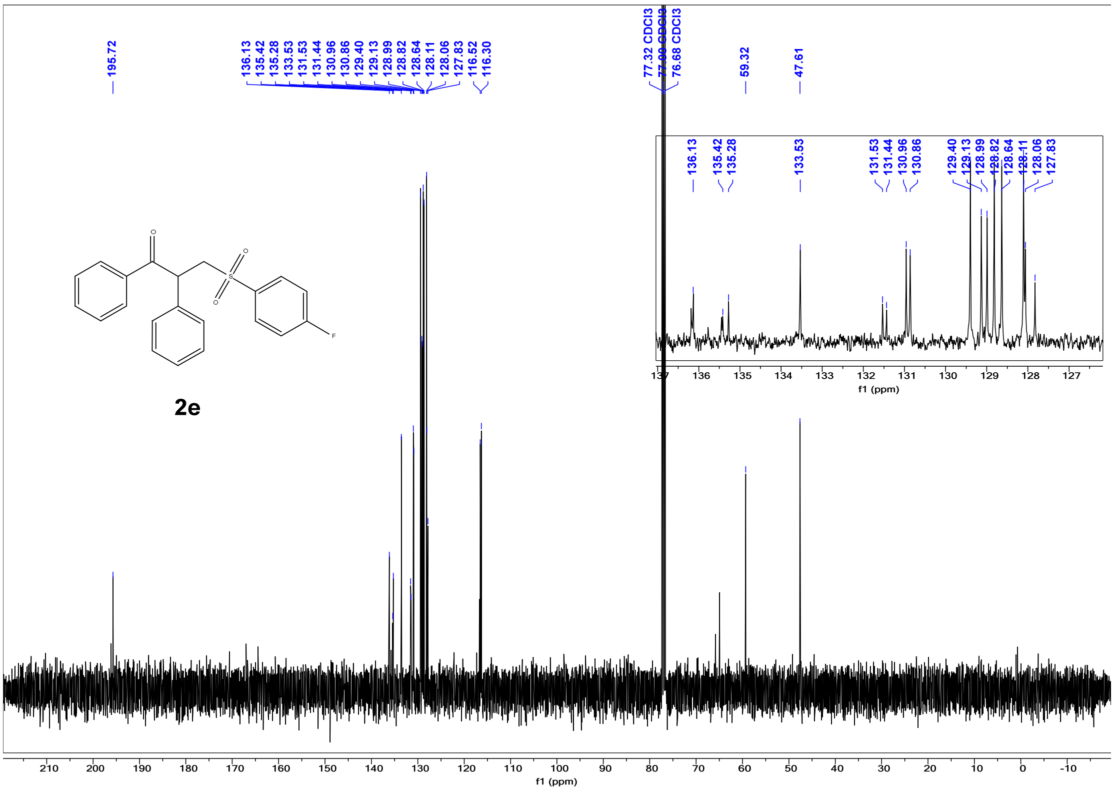
2e
f1 (ppm)
195.72
77.32 CDCl3
76.68 CDCl3
59.32
47.61

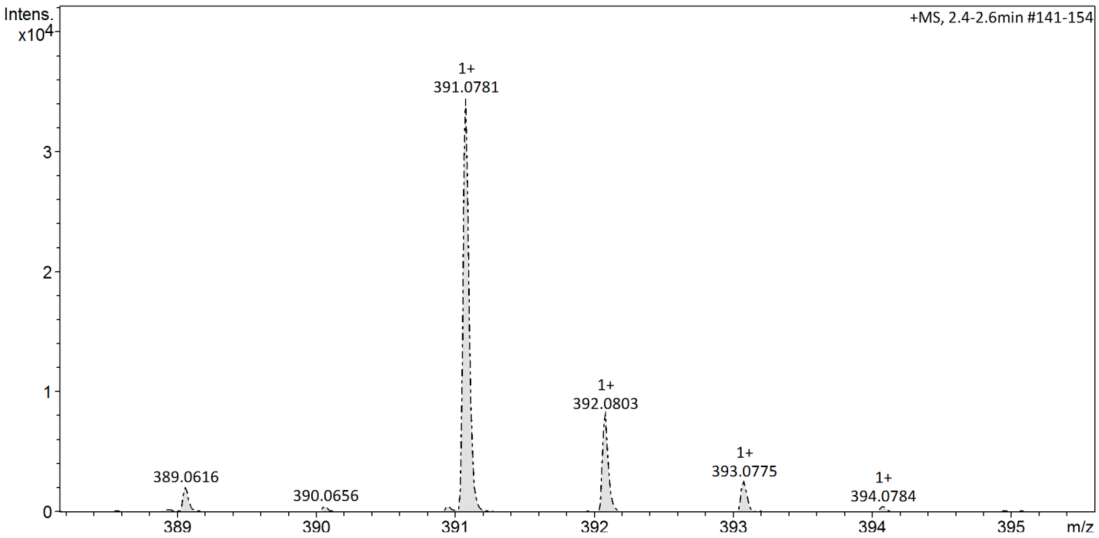
Intens.
x10^4
+MS, 2.4-2.6min #141-154
1+
391.0781
1+
392.0803
1+
393.0775
1+
394.0784
389.0616
390.0656
0
1
2
3
389
390
391
392
393
394
395
m/z

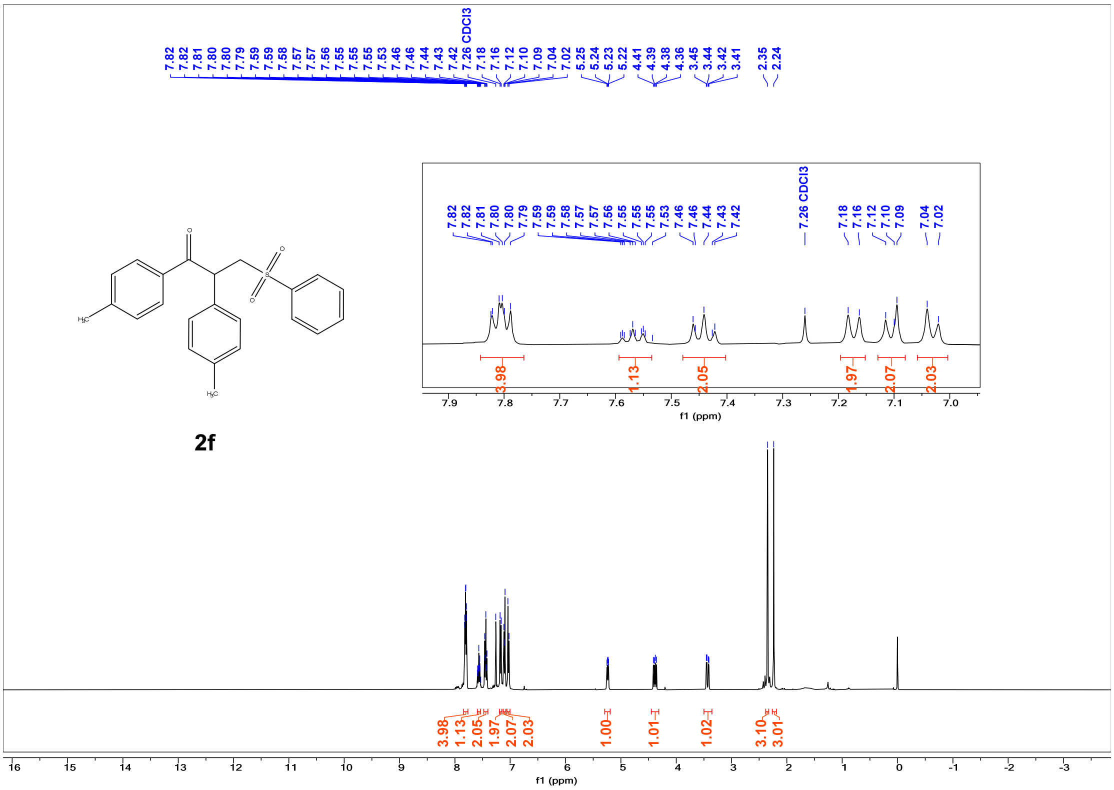

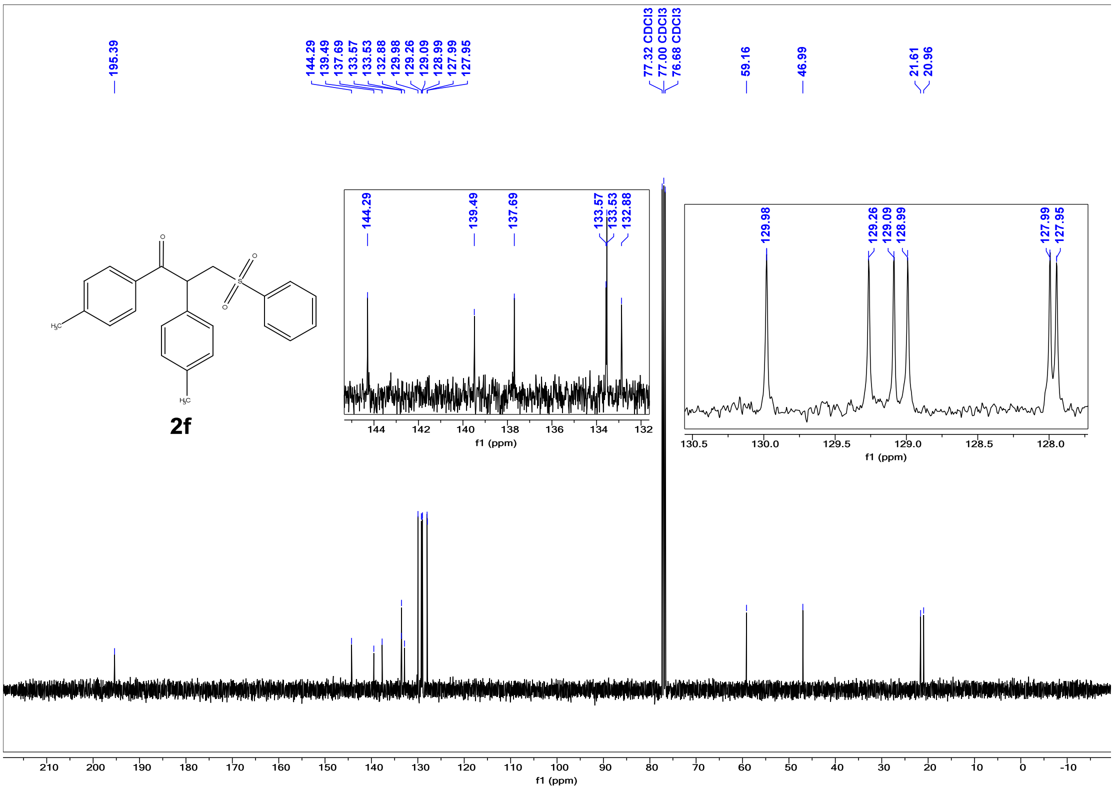

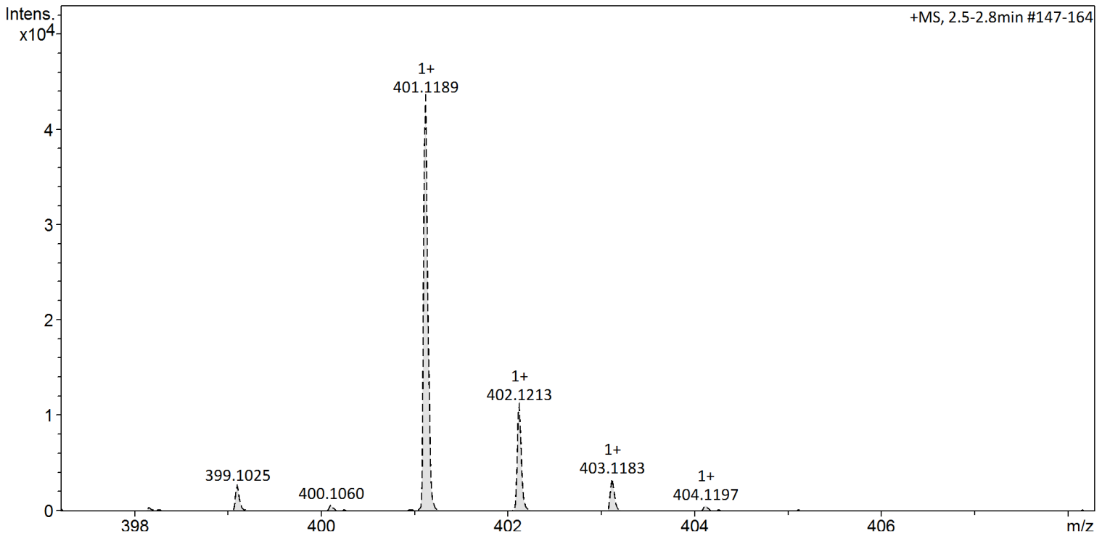
Intens.
x10^4
+MS, 2.5-2.8min #147-164
1+
401.1189
1+
402.1213
1+
403.1183
1+
404.1197
399.1025
400.1060
0
1
2
3
4
398
400
402
404
406
m/z

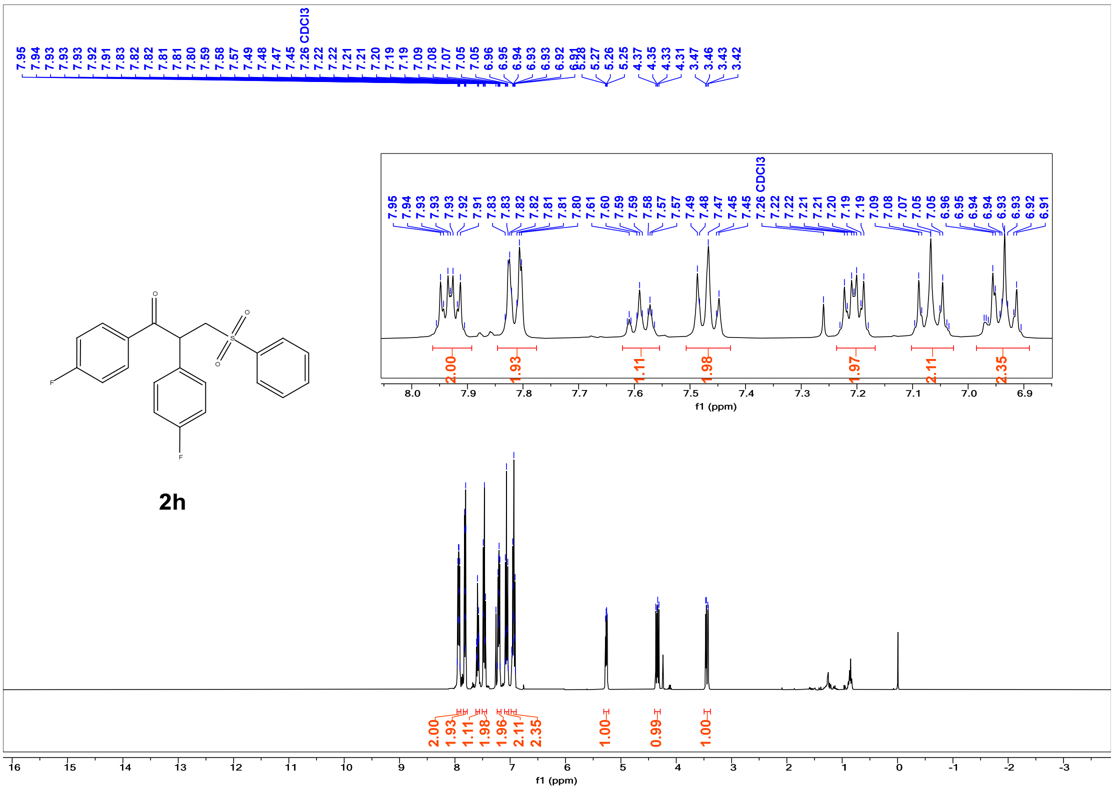

2h
f1 (ppm)

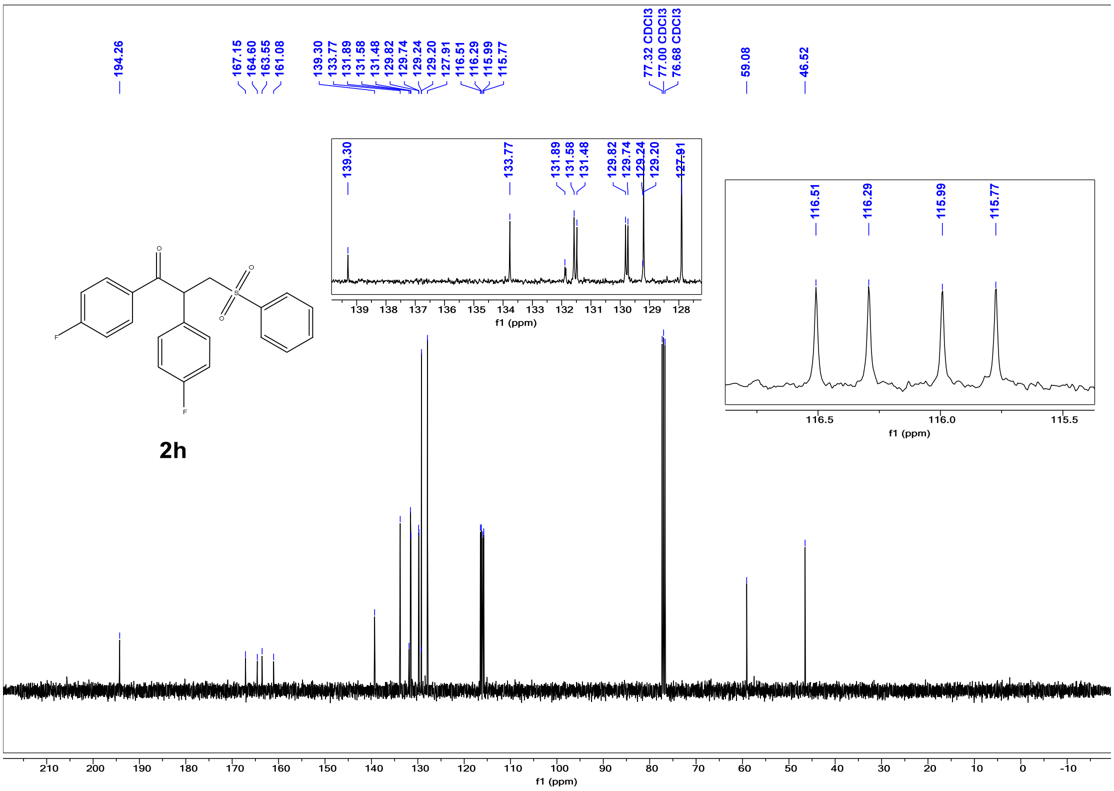

2h
194.26
59.08
46.52
f1 (ppm)

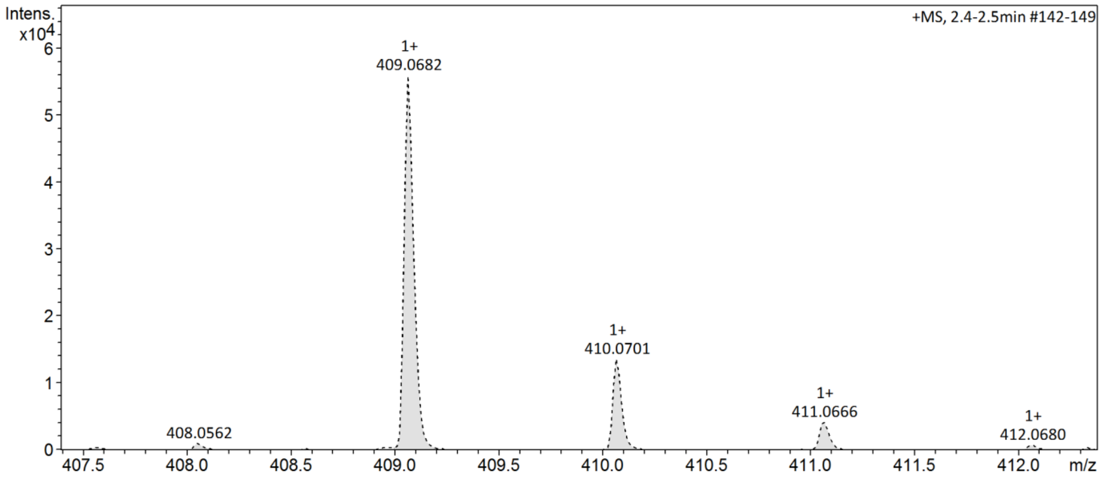

Intens.
x10^4
+MS, 2.4-2.5min #142-149
1+
409.0682
1+
410.0701
1+
411.0666
1+
412.0680
408.0562
m/z

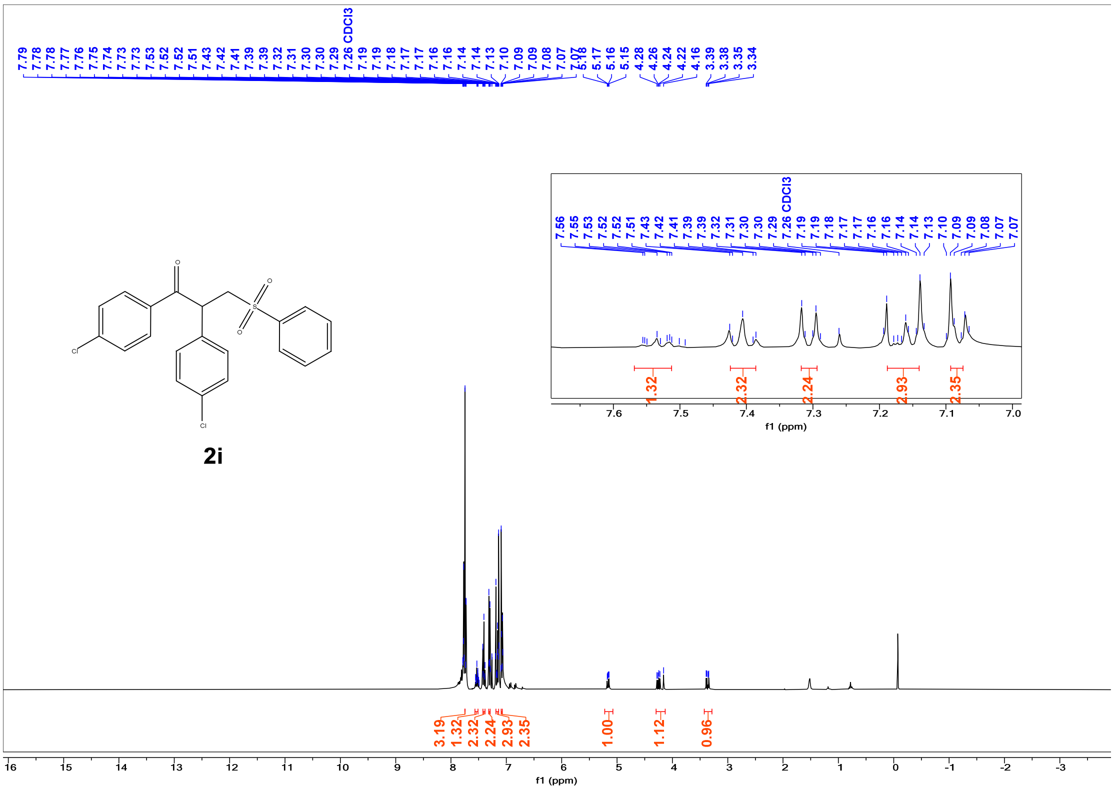
7.79 7.78 7.78 7.77 7.76 7.75 7.74 7.73 7.73 7.53 7.52 7.52 7.51 7.43 7.42 7.41 7.39 7.39 7.32 7.31 7.30 7.30 7.29 7.26 CDCl3 7.19 7.19 7.18 7.17 7.17 7.16 7.16 7.14 7.14 7.13 7.10 7.09 7.09 7.08 7.07 7.07 5.18 5.17 5.16 5.15 4.28 4.26 4.24 4.22 4.16 3.39 3.38 3.35 3.34
2i
3.19 1.32 2.32 2.24 2.93 2.35 1.00 1.12 0.96
f1 (ppm)

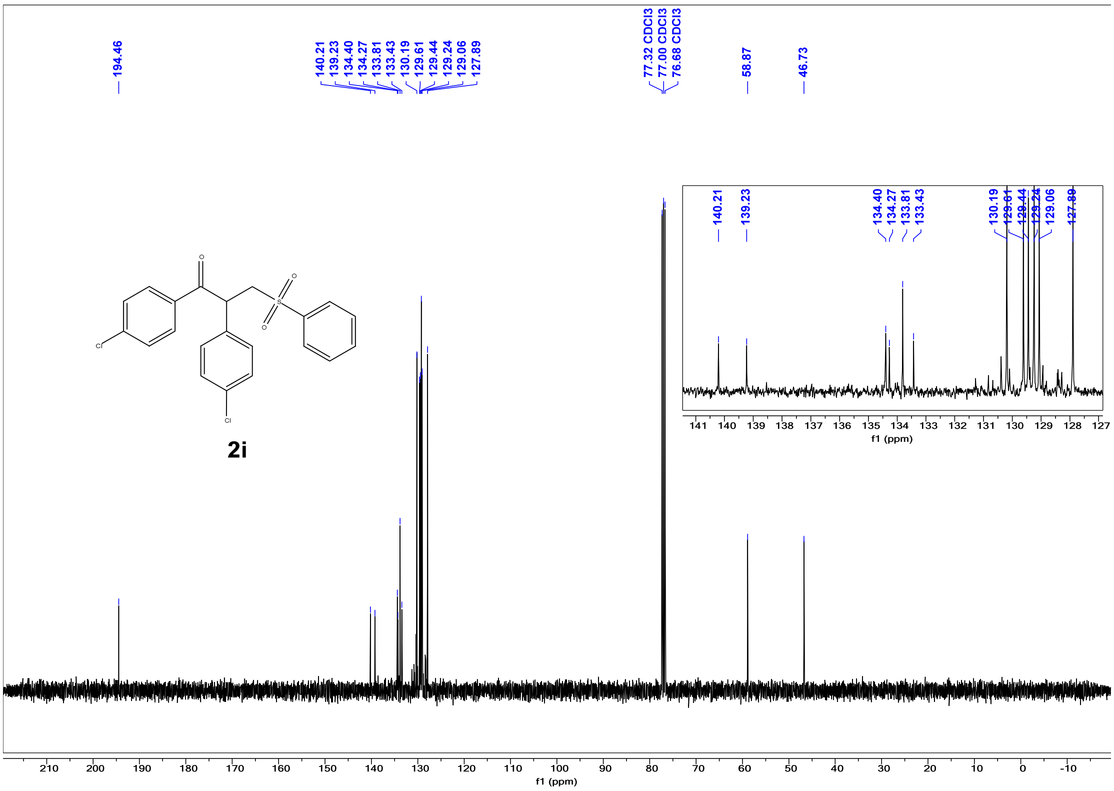
194.46
140.21 139.23 134.40 134.27 133.81 133.43 130.19 129.61 129.44 129.24 129.06 127.89
77.32 CDCl3 77.00 CDCl3 76.68 CDCl3
58.87
46.73
2i
f1 (ppm)

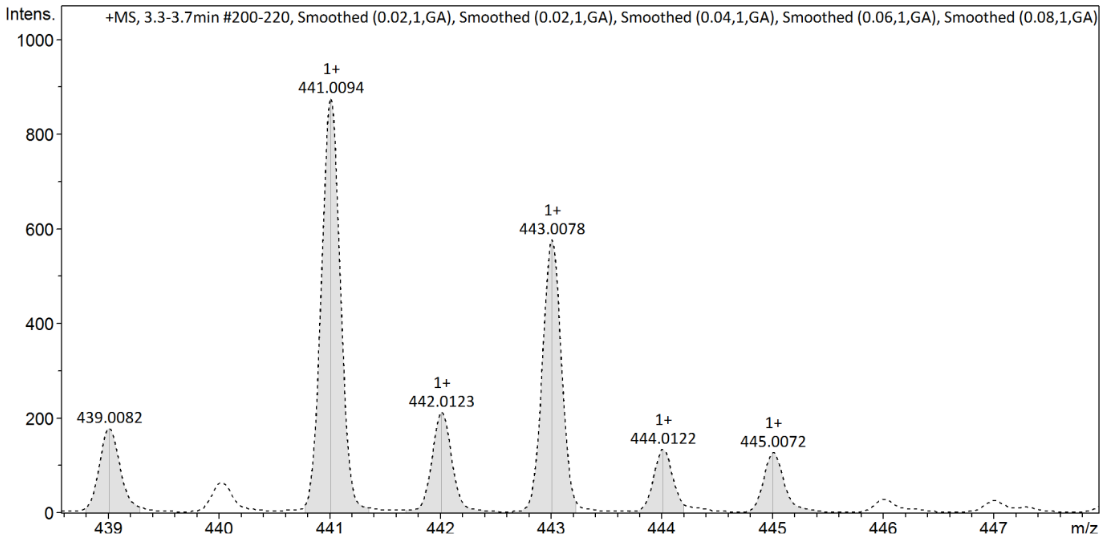
Intens.
+MS, 3.3-3.7min #200-220, Smoothed (0.02,1,GA), Smoothed (0.02,1,GA), Smoothed (0.04,1,GA), Smoothed (0.06,1,GA), Smoothed (0.08,1,GA)
1000
800
600
400
200
0
439.0082
1+
441.0094
1+
442.0123
1+
443.0078
1+
444.0122
1+
445.0072
439
440
441
442
443
444
445
446
447
m/z

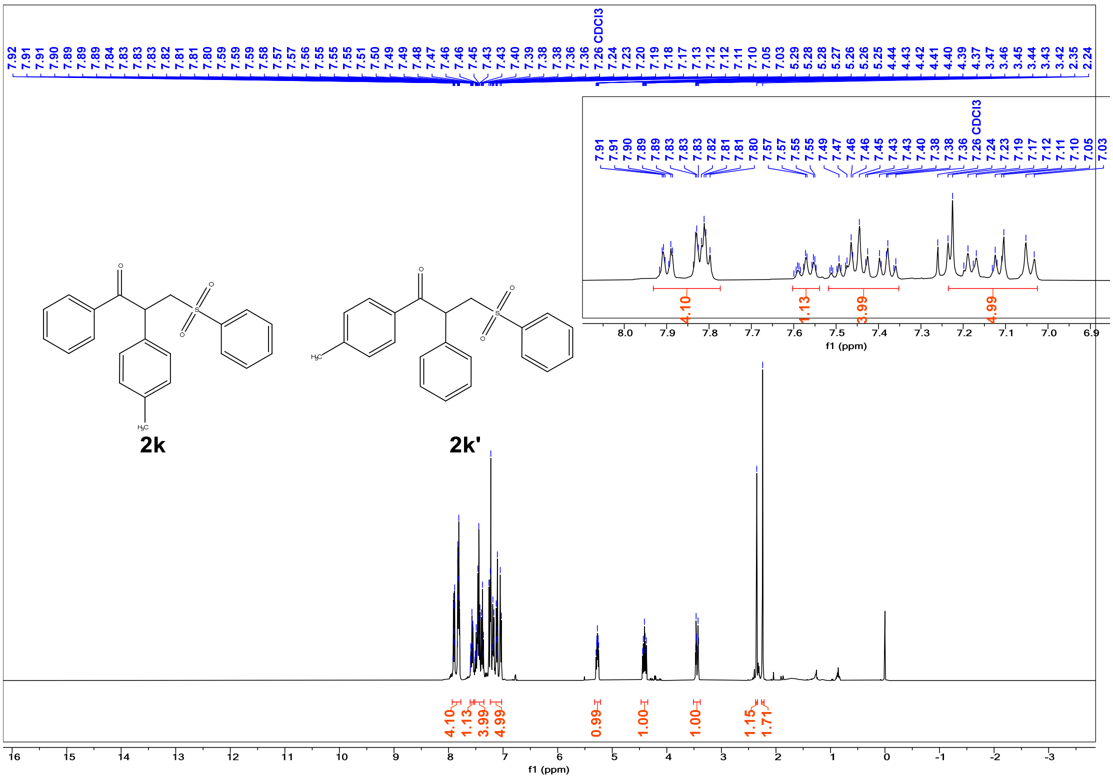

7.92 7.91 7.91 7.90 7.89 7.89 7.89 7.84 7.83 7.83 7.83 7.82 7.81 7.81 7.80 7.59 7.59 7.59 7.58 7.57 7.57 7.56 7.55 7.55 7.55 7.51 7.50 7.49 7.49 7.48 7.47 7.46 7.45 7.43 7.43 7.40 7.39 7.38 7.38 7.36 7.36 7.26 CDCl3 7.24 7.23 7.20 7.19 7.18 7.17 7.13 7.12 7.12 7.11 7.10 7.05 7.03 5.29 5.28 5.28 5.27 5.26 5.25 4.44 4.43 4.42 4.41 4.40 4.39 4.37 3.47 3.46 3.45 3.44 3.43 3.42 2.35 2.24
4.10 1.13 3.99 4.99 0.99 1.00 1.00 1.15 1.71
2k
2k'
f1 (ppm)

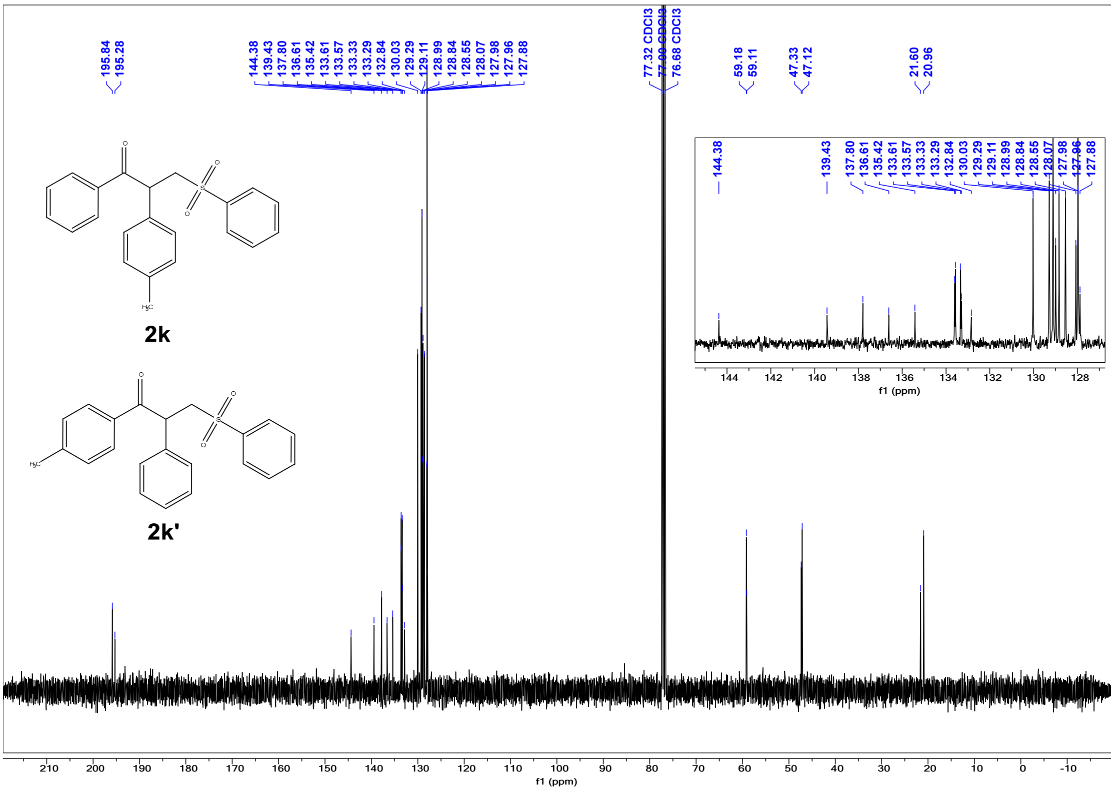

195.84 195.28
144.38 139.43 137.80 136.61 135.42 133.61 133.57 133.33 133.29 132.84 130.03 129.29 129.11 128.99 128.84 128.55 128.07 127.98 127.96 127.88
77.32 CDCl3 77.00 CDCl3 76.68 CDCl3
59.18 59.11
47.33 47.12
21.60 20.96
2k
2k'
f1 (ppm)

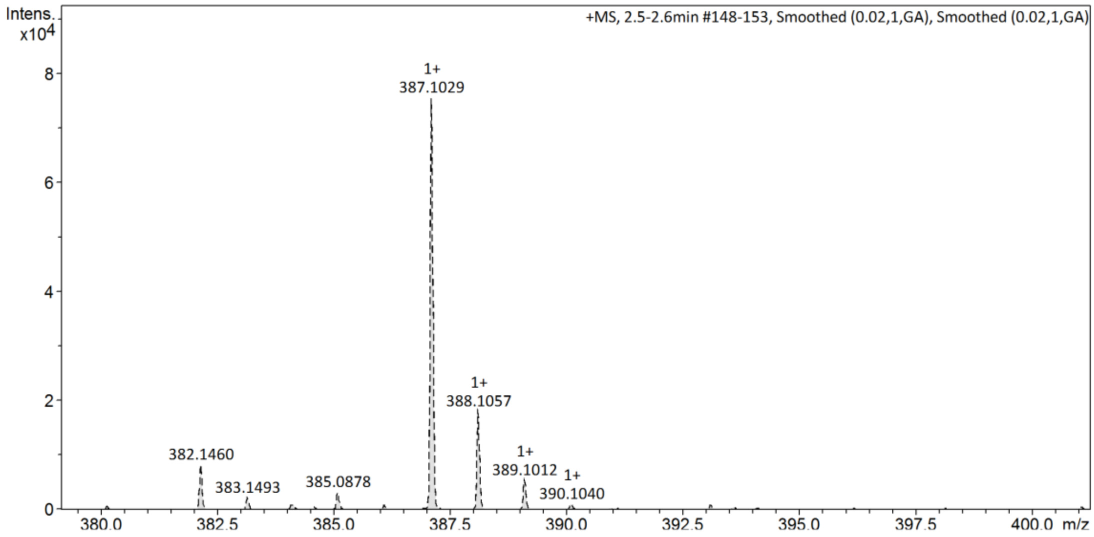
Intens.
x10^4
+MS, 2.5-2.6min #148-153, Smoothed (0.02,1,GA), Smoothed (0.02,1,GA)
8
6
4
2
0
1+
387.1029
1+
388.1057
1+
389.1012
1+
390.1040
382.1460
383.1493
385.0878
380.0
382.5
385.0
387.5
390.0
392.5
395.0
397.5
400.0
m/z

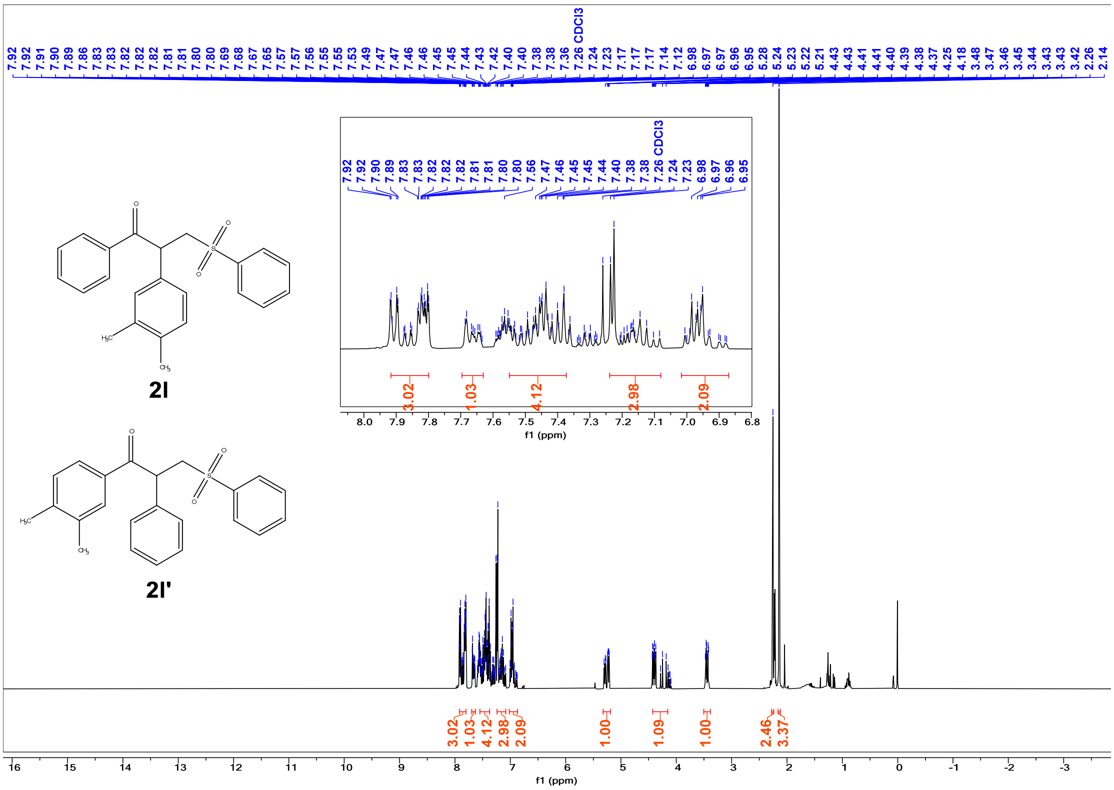

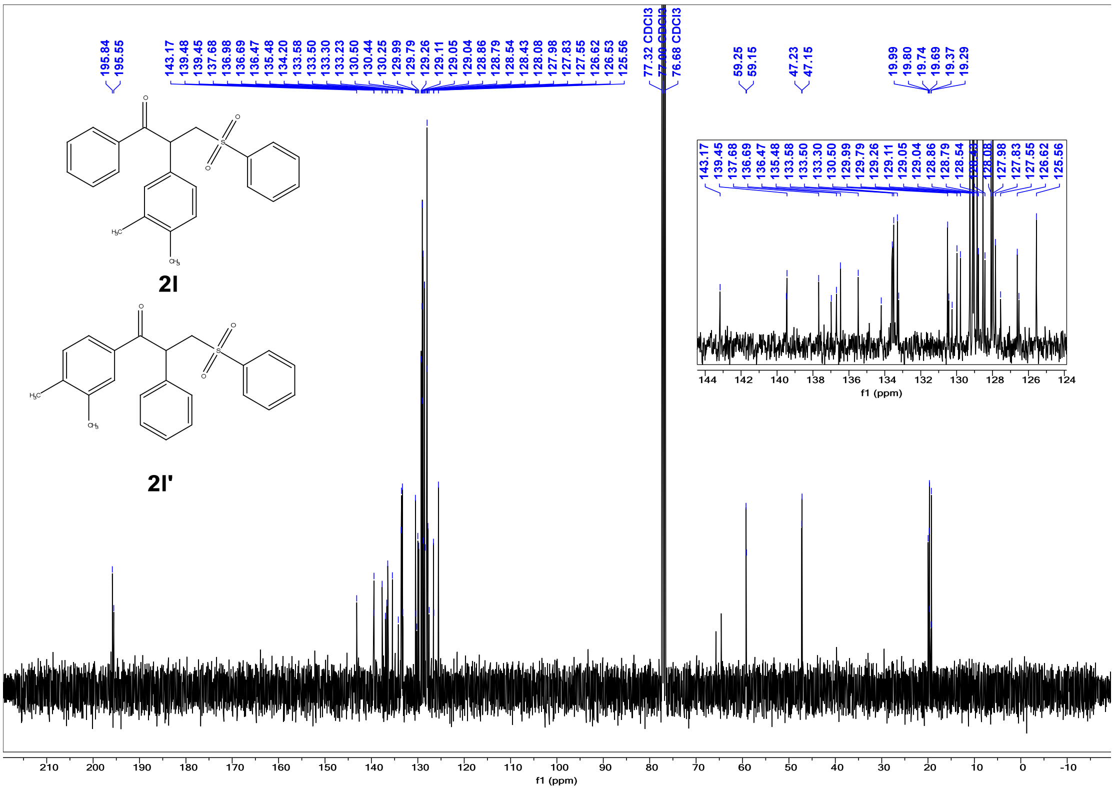

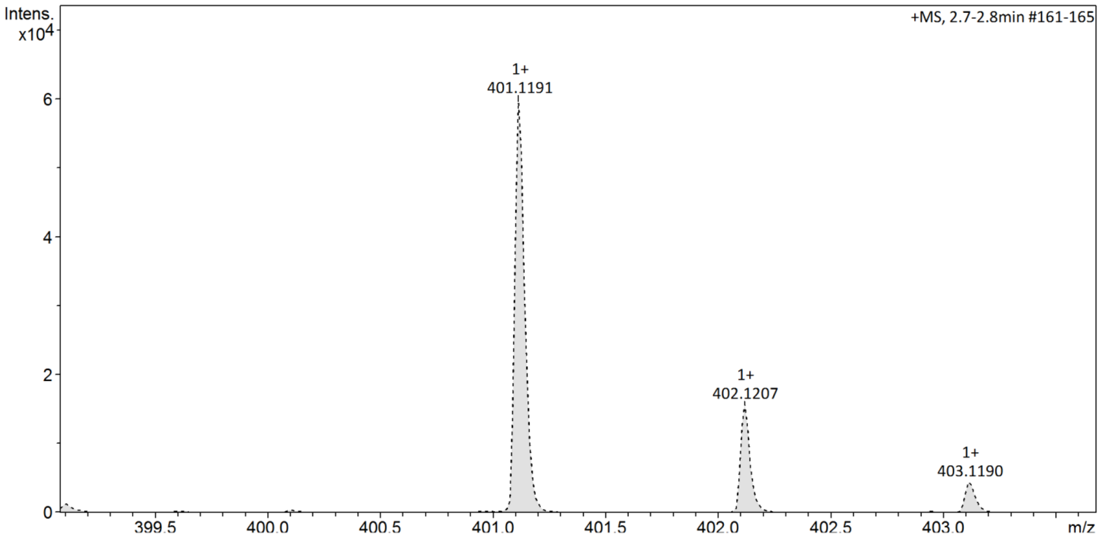
Intens.
x10^4
+MS, 2.7-2.8min #161-165
1+
401.1191
1+
402.1207
1+
403.1190
6
4
2
0
399.5
400.0
400.5
401.0
401.5
402.0
402.5
403.0
m/z

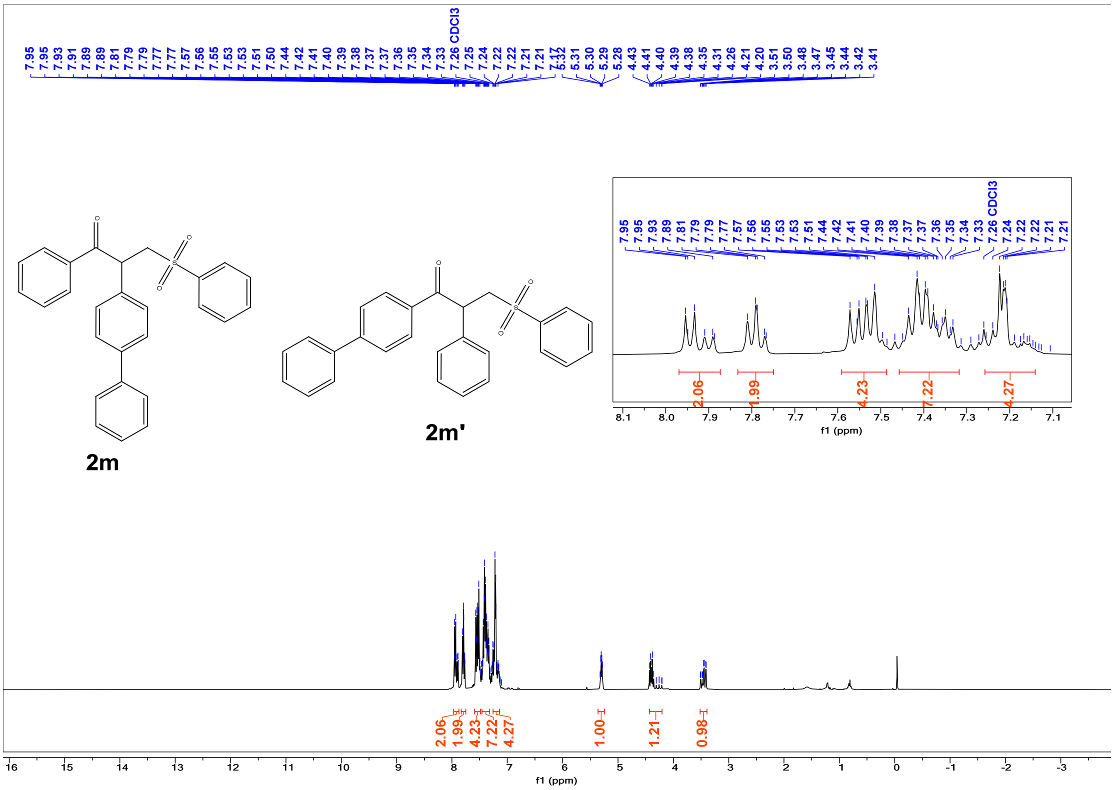
7.95 7.95 7.93 7.91 7.89 7.81 7.79 7.79 7.77 7.77 7.57 7.56 7.55 7.53 7.53 7.51 7.50 7.44 7.42 7.41 7.40 7.39 7.38 7.37 7.37 7.36 7.35 7.34 7.33 7.26 CDCl3 7.25 7.24 7.22 7.22 7.21 7.21 7.17 5.32 5.31 5.30 5.29 5.28 4.43 4.41 4.40 4.39 4.38 4.35 4.31 4.26 4.21 4.20 3.51 3.50 3.48 3.47 3.45 3.44 3.42 3.41
2m
2m'
2.06 1.99 4.23 7.22 4.27 1.00 1.21 0.98
f1 (ppm)
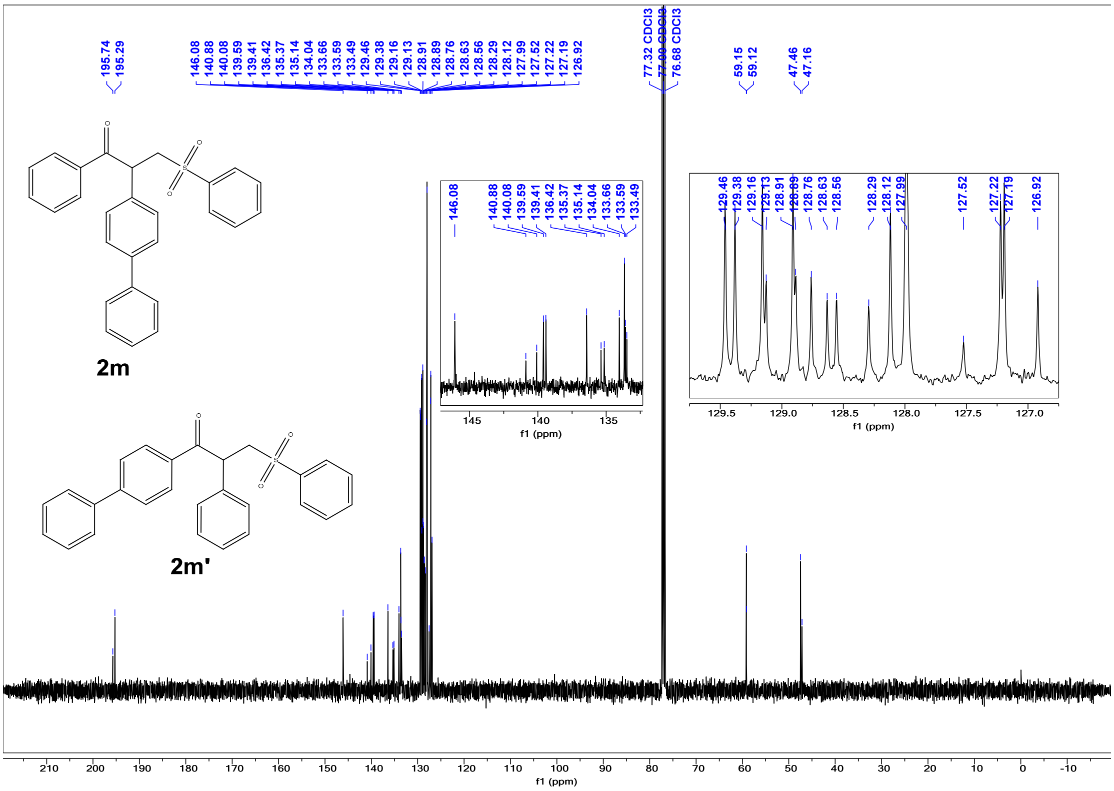
195.74 195.29
146.08 140.88 140.08 139.59 139.41 136.42 135.37 135.14 134.04 133.66 133.59 133.49 129.46 129.38 129.16 129.13 128.91 128.89 128.76 128.63 128.56 128.29 128.12 127.99 127.52 127.22 127.19 126.92
77.32 CDCl3 77.00 CDCl3 76.68 CDCl3
59.15 59.12
47.46 47.16
2m
2m'
f1 (ppm)

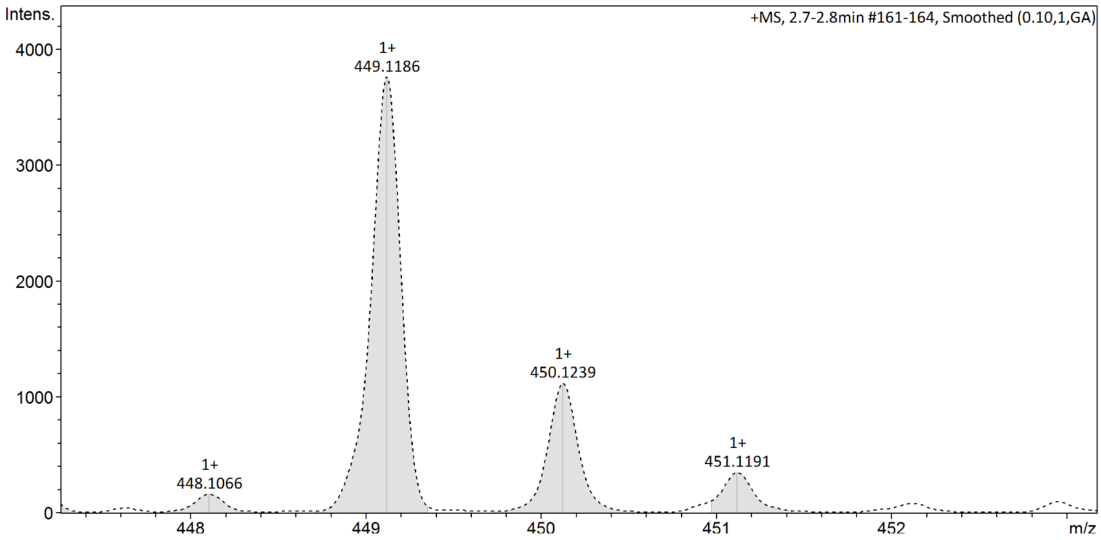

Intens.
+MS, 2.7-2.8min #161-164, Smoothed (0.10,1,GA)
4000
3000
2000
1000
0
1+
449.1186
1+
450.1239
1+
448.1066
1+
451.1191
448
449
450
451
452
m/z

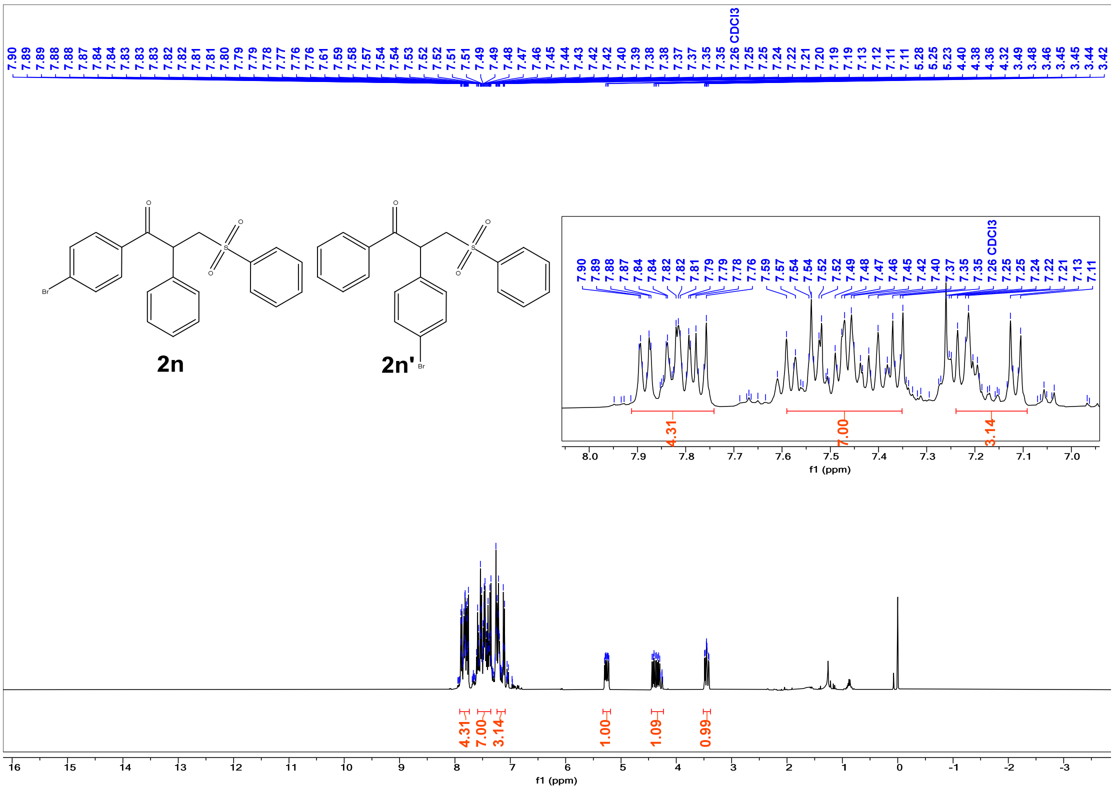
2n
2n'
Br
7.26 CDCl3
4.31
7.00
3.14
1.00
1.09
0.99
f1 (ppm)

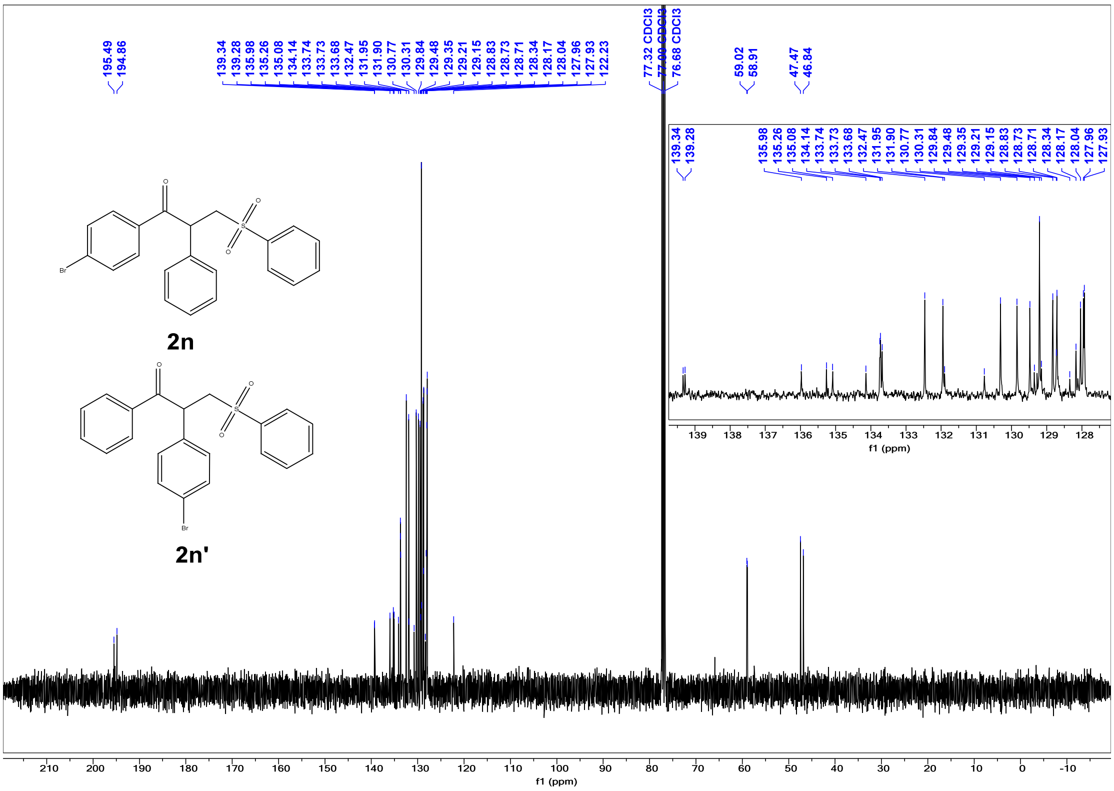
2n
2n'
Br
195.49
194.86
77.32 CDCl3
76.68 CDCl3
59.02
58.91
47.47
46.84
f1 (ppm)

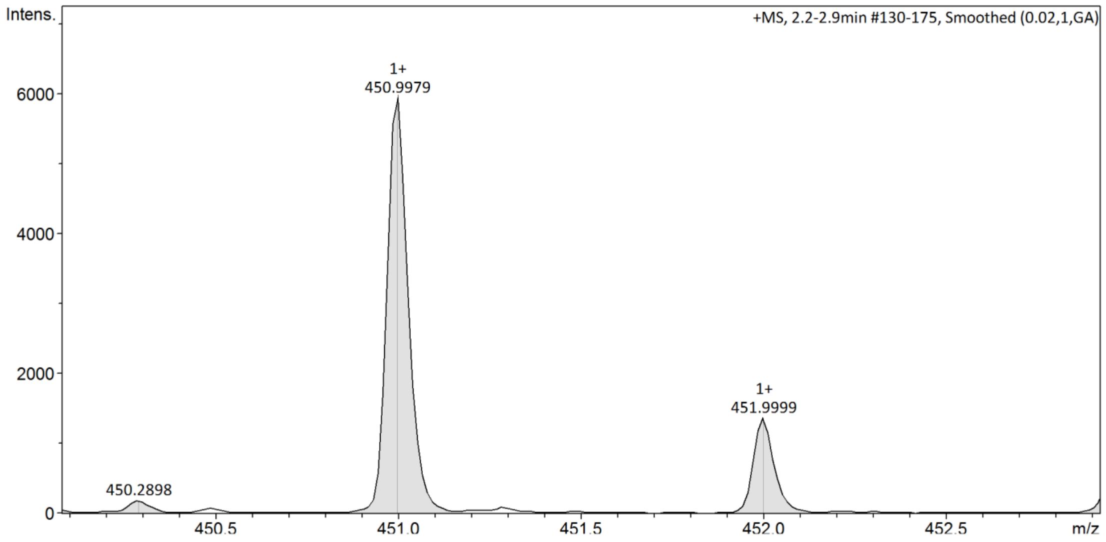
Intens.
+MS, 2.2-2.9min #130-175, Smoothed (0.02,1,GA)
6000
4000
2000
0
1+
450.9979
1+
451.9999
450.2898
450.5
451.0
451.5
452.0
452.5
m/z

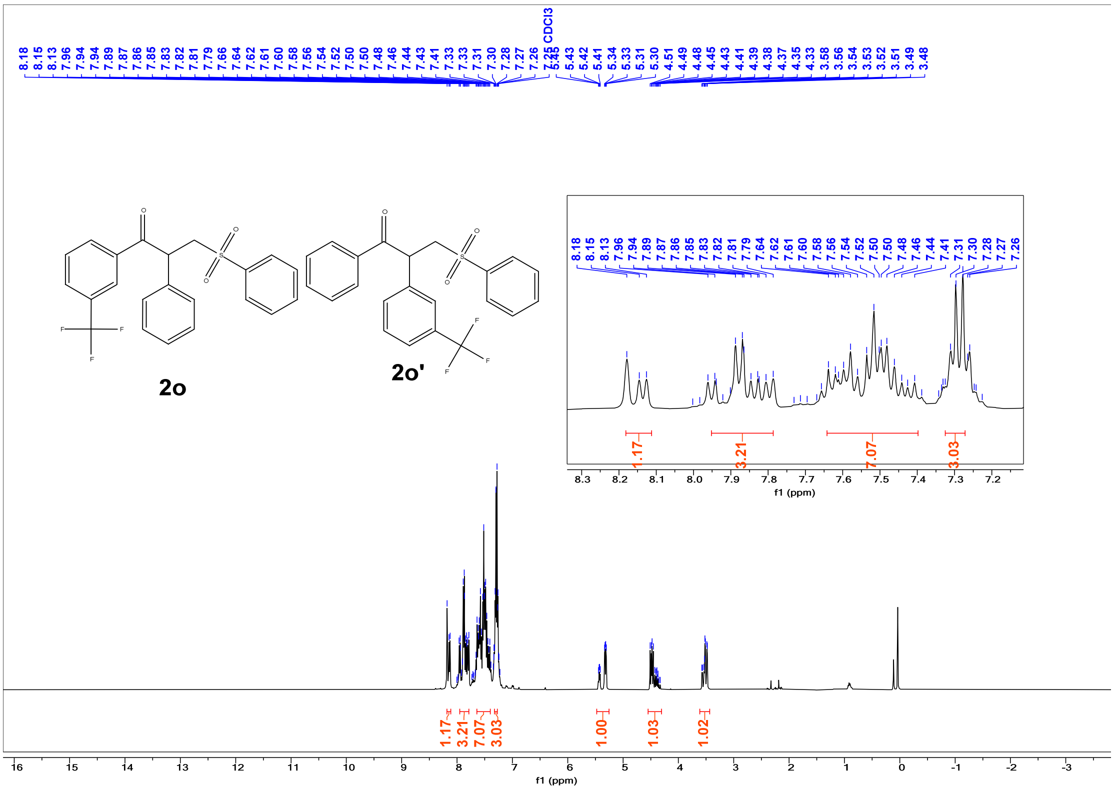

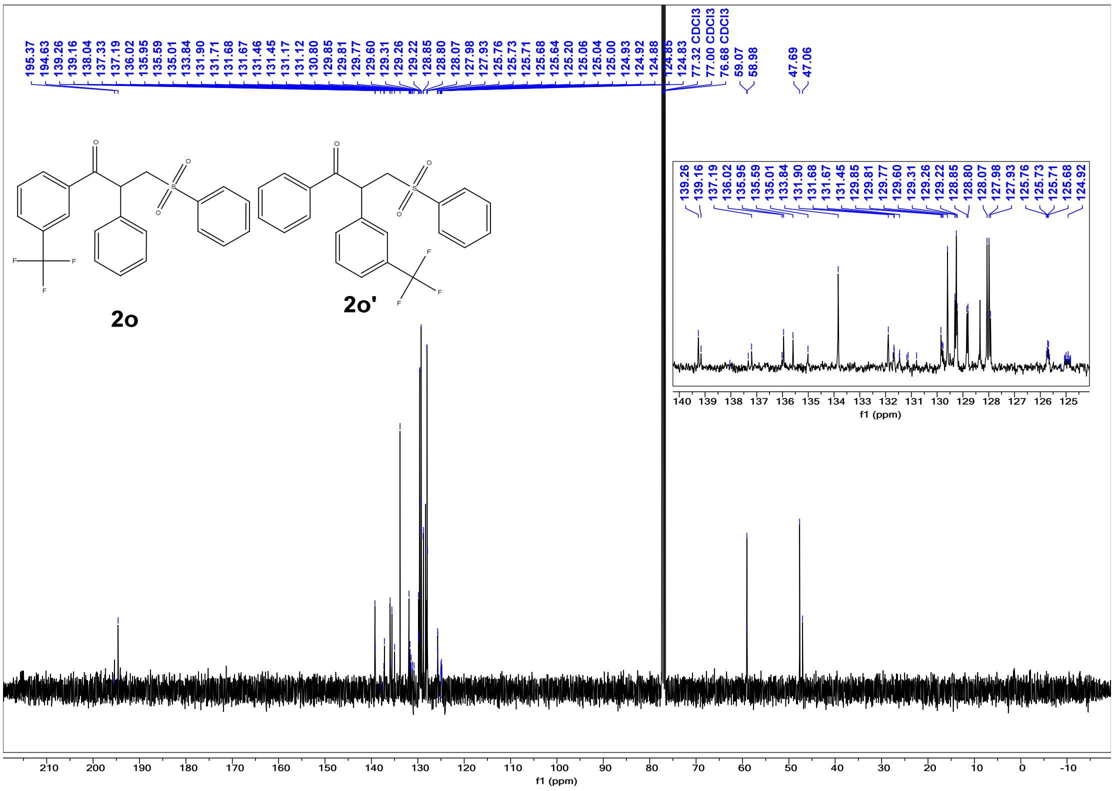

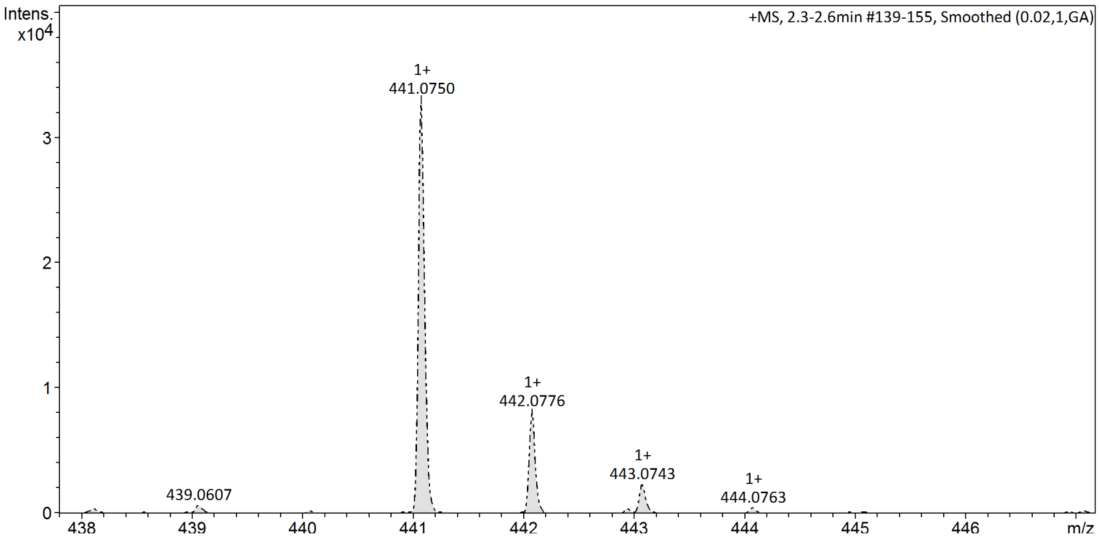

Intens.
x10^4
+MS, 2.3-2.6min #139-155, Smoothed (0.02,1,GA)
1+
441.0750
1+
442.0776
1+
443.0743
1+
444.0763
439.0607
3
2
1
0
438
439
440
441
442
443
444
445
446
m/z

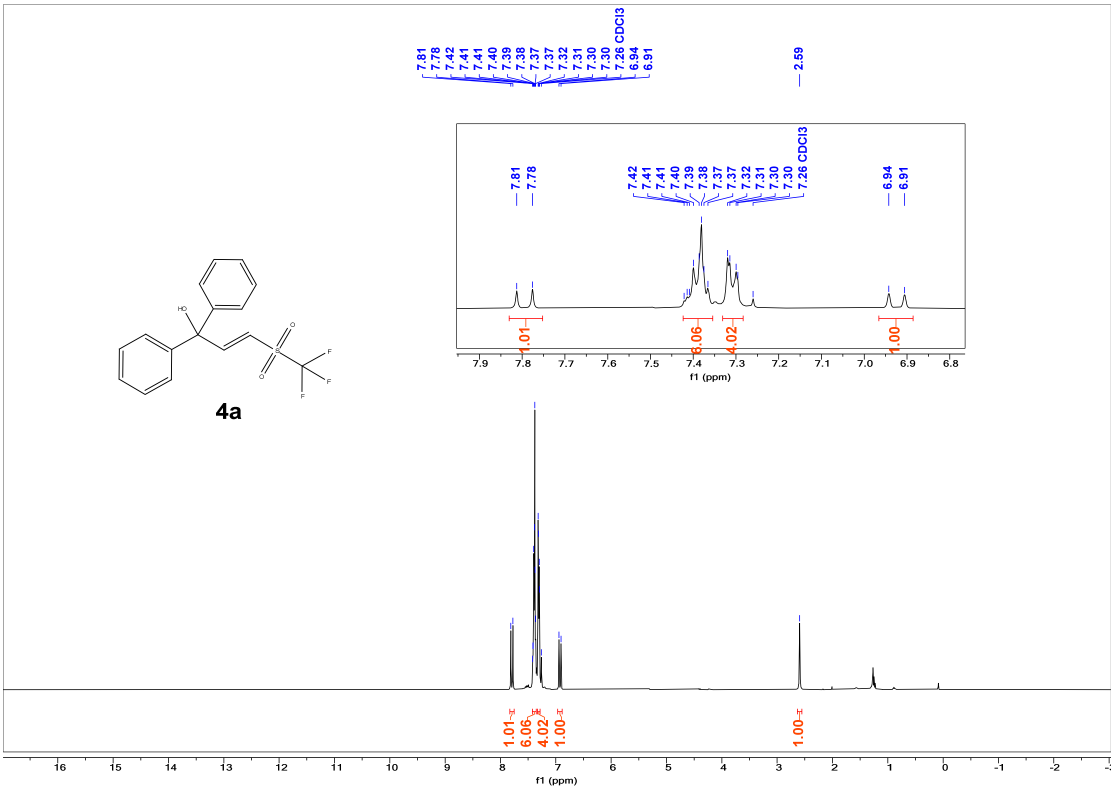
7.81
7.78
7.42
7.41
7.41
7.40
7.39
7.38
7.37
7.37
7.32
7.31
7.30
7.30
7.26 CDCl3
6.94
6.91
2.59
4a
1.01
6.06
4.02
1.00
1.00
f1 (ppm)

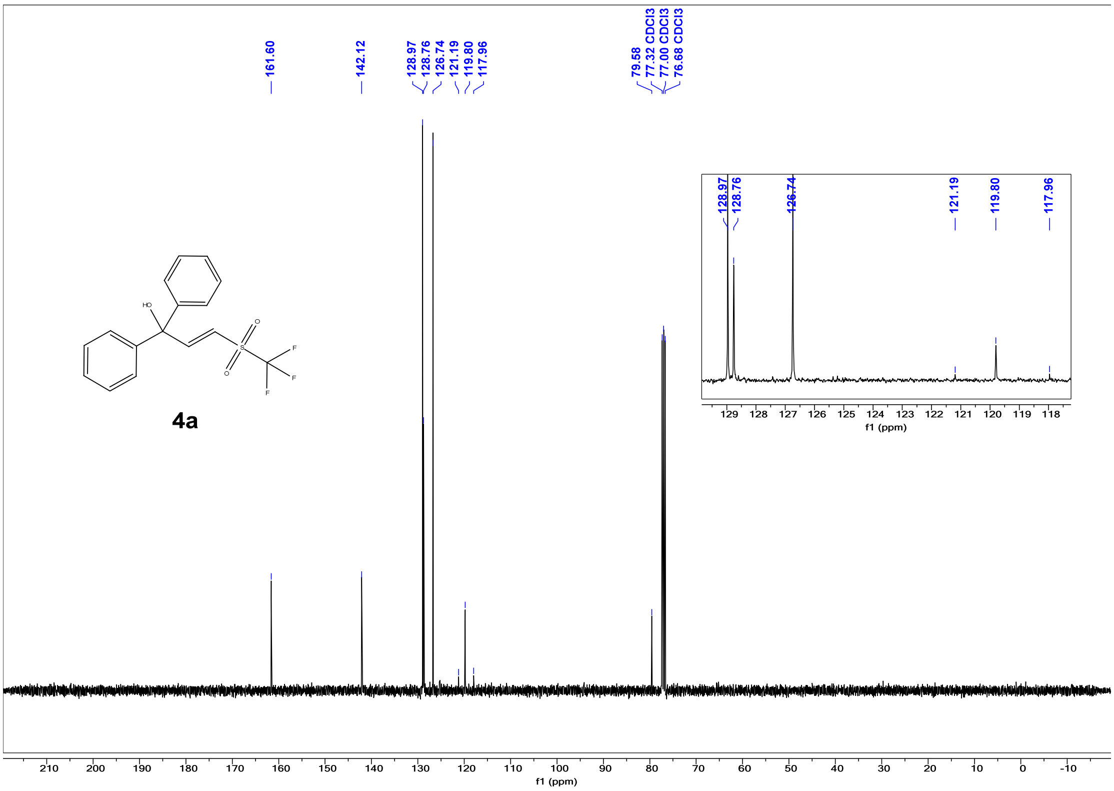
161.60
142.12
128.97
128.76
126.74
121.19
119.80
117.96
79.58
77.32 CDCl3
77.00 CDCl3
76.68 CDCl3
4a
f1 (ppm)

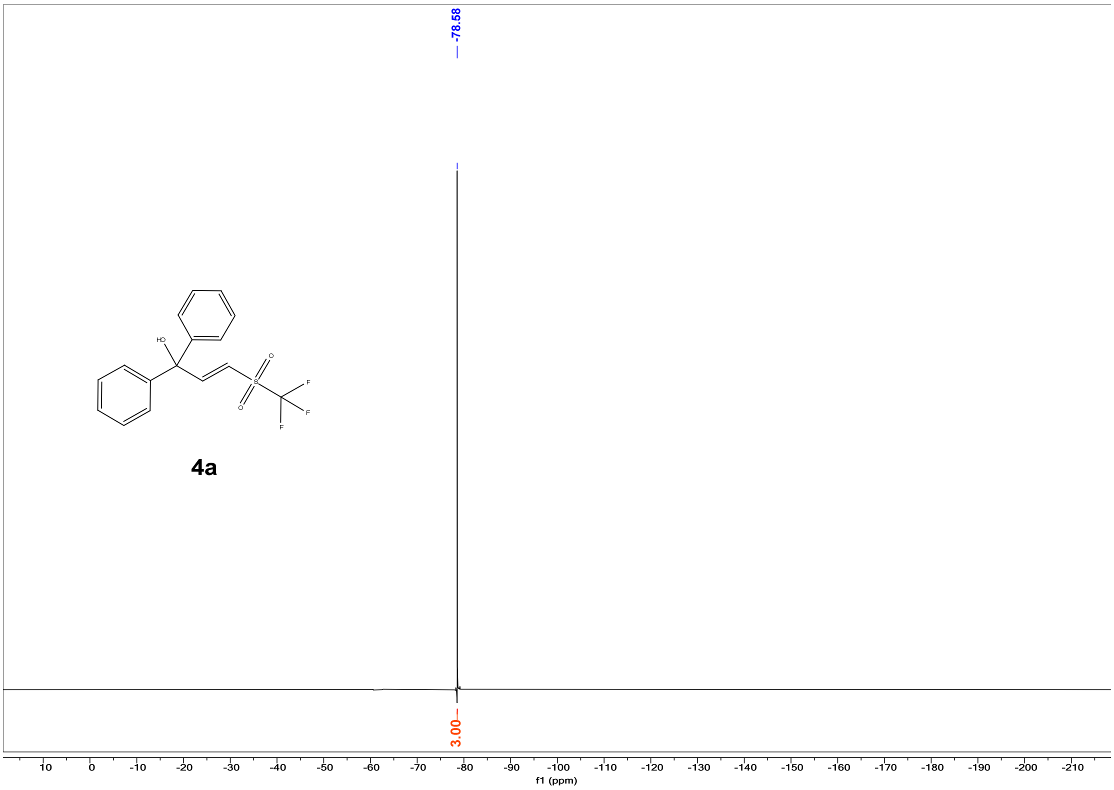

-78.58
HO
O
O
F
F
F
4a
3.00
10 0 -10 -20 -30 -40 -50 -60 -70 -80 -90 -100 -110 -120 -130 -140 -150 -160 -170 -180 -190 -200 -210
f1 (ppm)

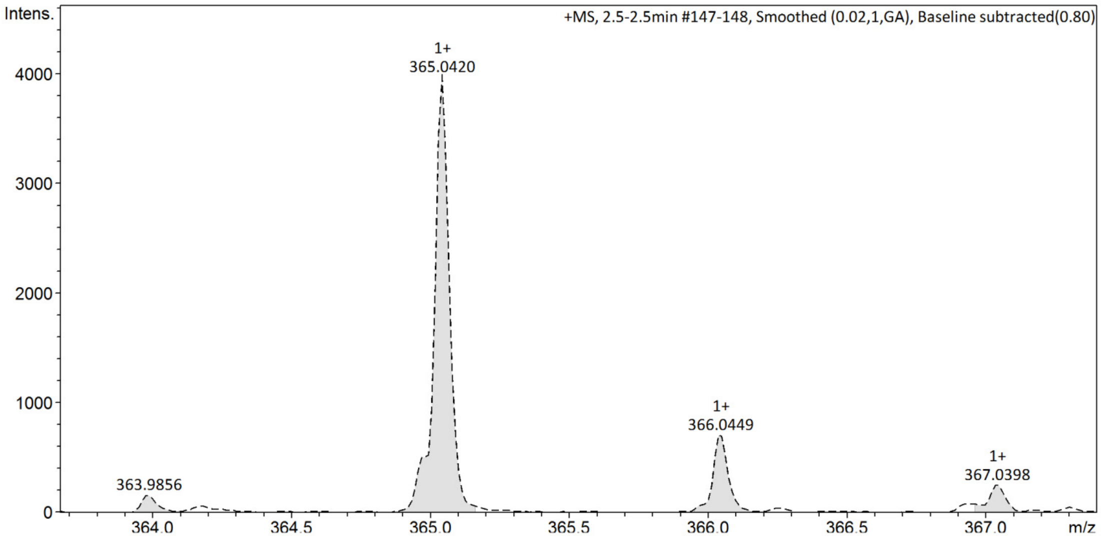

Intens.
+MS, 2.5-2.5min #147-148, Smoothed (0.02,1,GA), Baseline subtracted(0.80)
4000
3000
2000
1000
0
1+
365.0420
1+
366.0449
1+
367.0398
363.9856
364.0 364.5 365.0 365.5 366.0 366.5 367.0
m/z

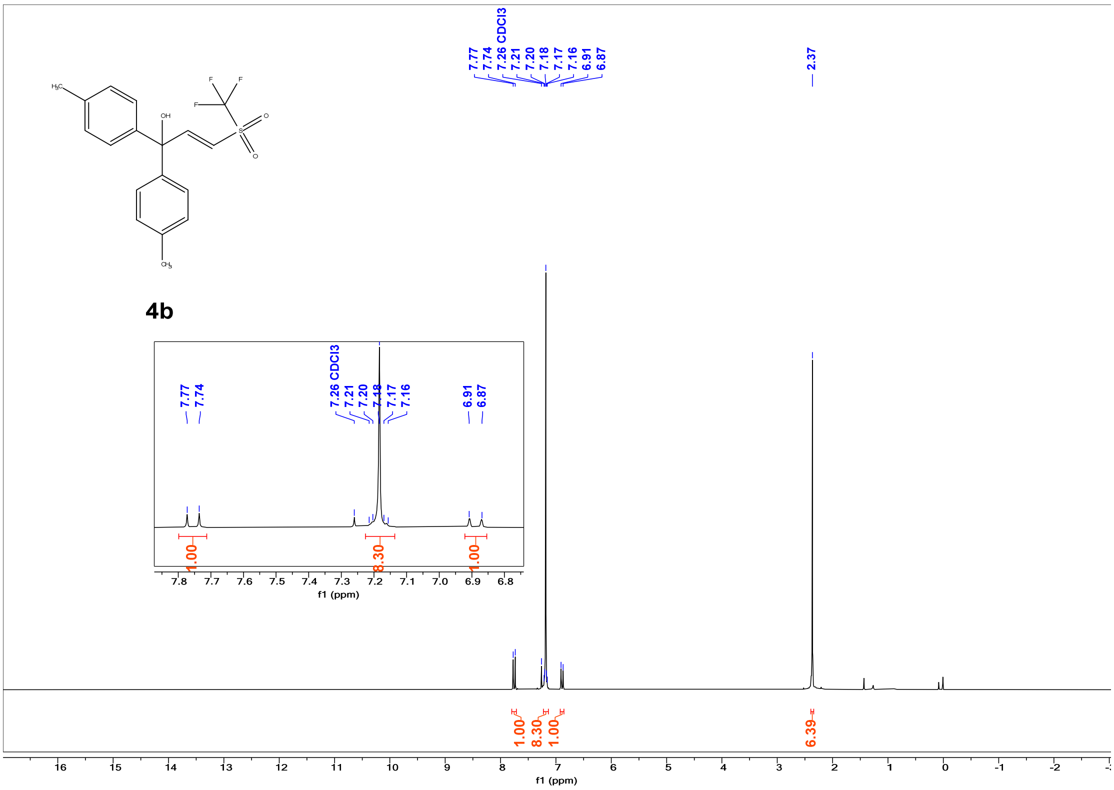
7.77
7.74
7.26 CDCl3
7.21
7.20
7.18
7.17
7.16
6.91
6.87
2.37
4b
1.00
8.30
1.00
6.39
f1 (ppm)

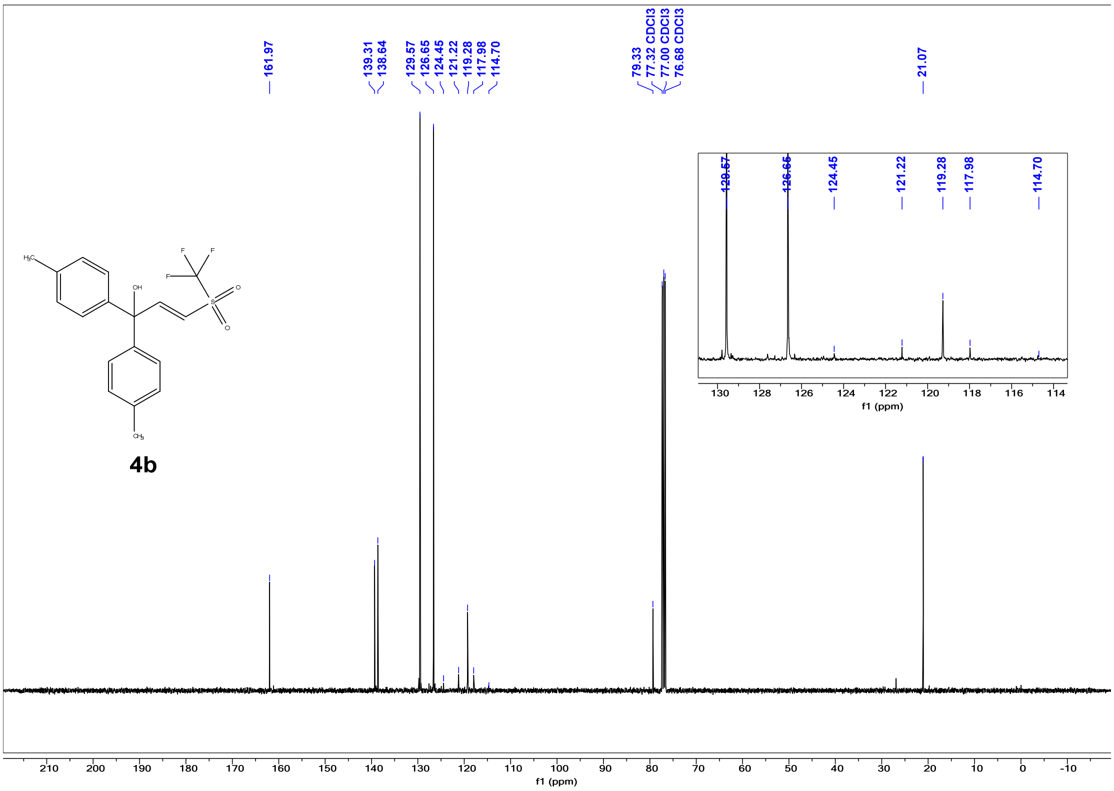
161.97
139.31
138.64
129.57
126.65
124.45
121.22
119.28
117.98
114.70
79.33
77.32 CDCl3
77.00 CDCl3
76.68 CDCl3
21.07
4b
f1 (ppm)

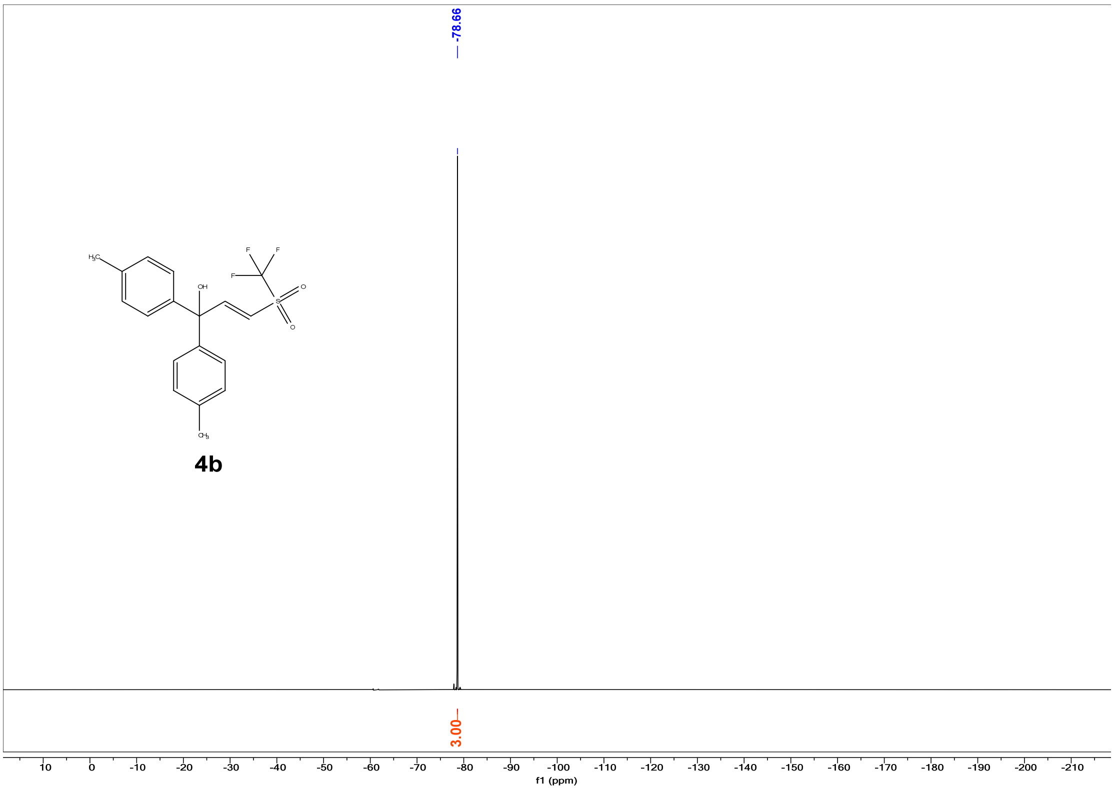

-78.66
4b
3.00
f1 (ppm)

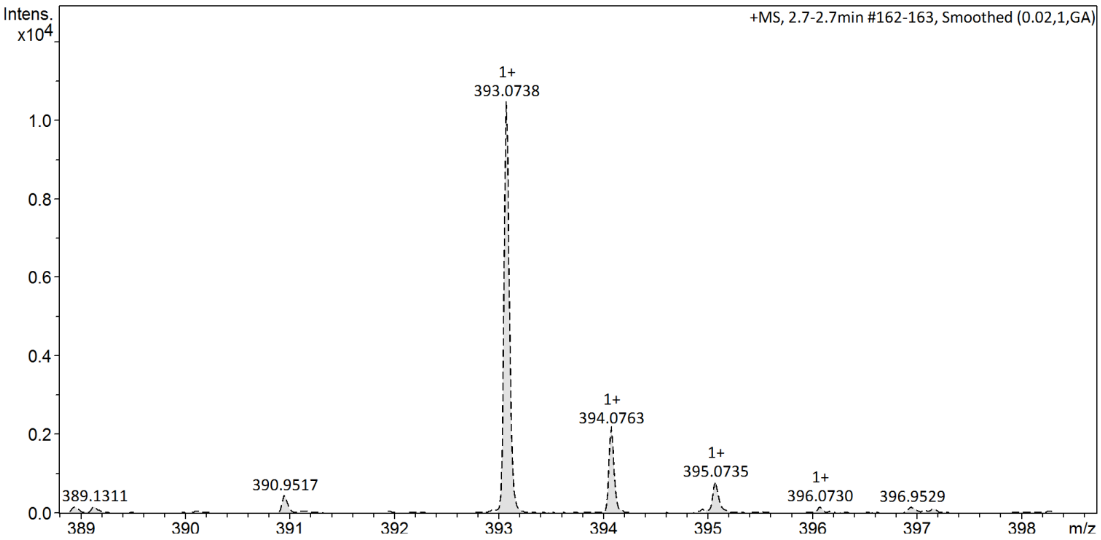

Intens.
x10^4
+MS, 2.7-2.7min #162-163, Smoothed (0.02,1,GA)
1+
393.0738
1+
394.0763
1+
395.0735
1+
396.0730
389.1311
390.9517
396.9529
m/z

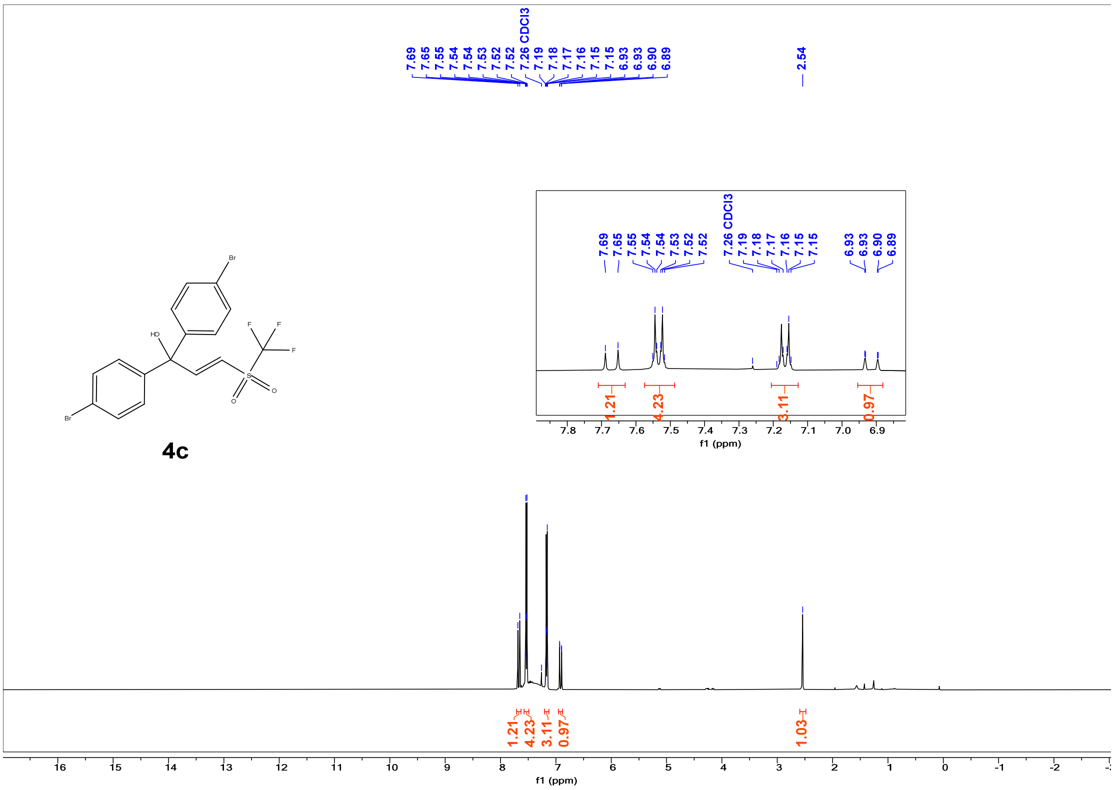
7.69
7.65
7.55
7.54
7.54
7.53
7.52
7.52
7.26 CDCl3
7.19
7.18
7.17
7.16
7.15
7.15
6.93
6.93
6.90
6.89
2.54
4c
1.21
4.23
3.11
0.97
1.03
f1 (ppm)

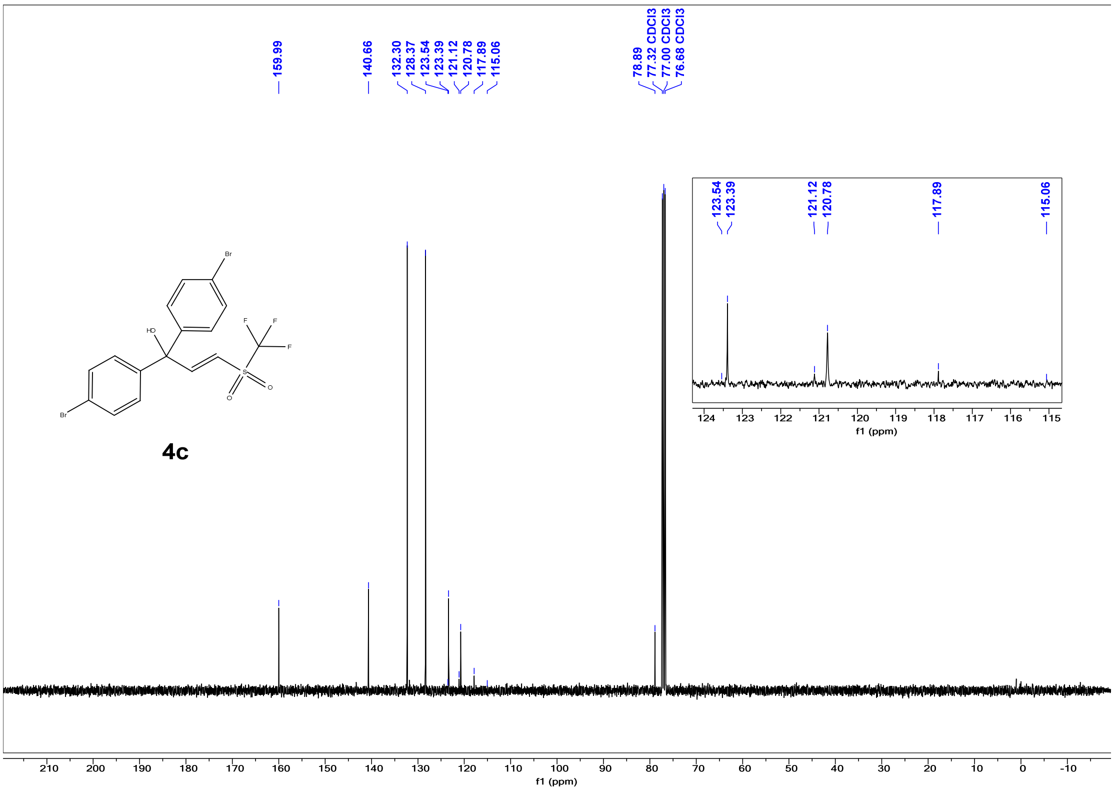
159.99
140.66
132.30
128.37
123.54
123.39
121.12
120.78
117.89
115.06
78.89
77.32 CDCl3
77.00 CDCl3
76.68 CDCl3
4c
f1 (ppm)

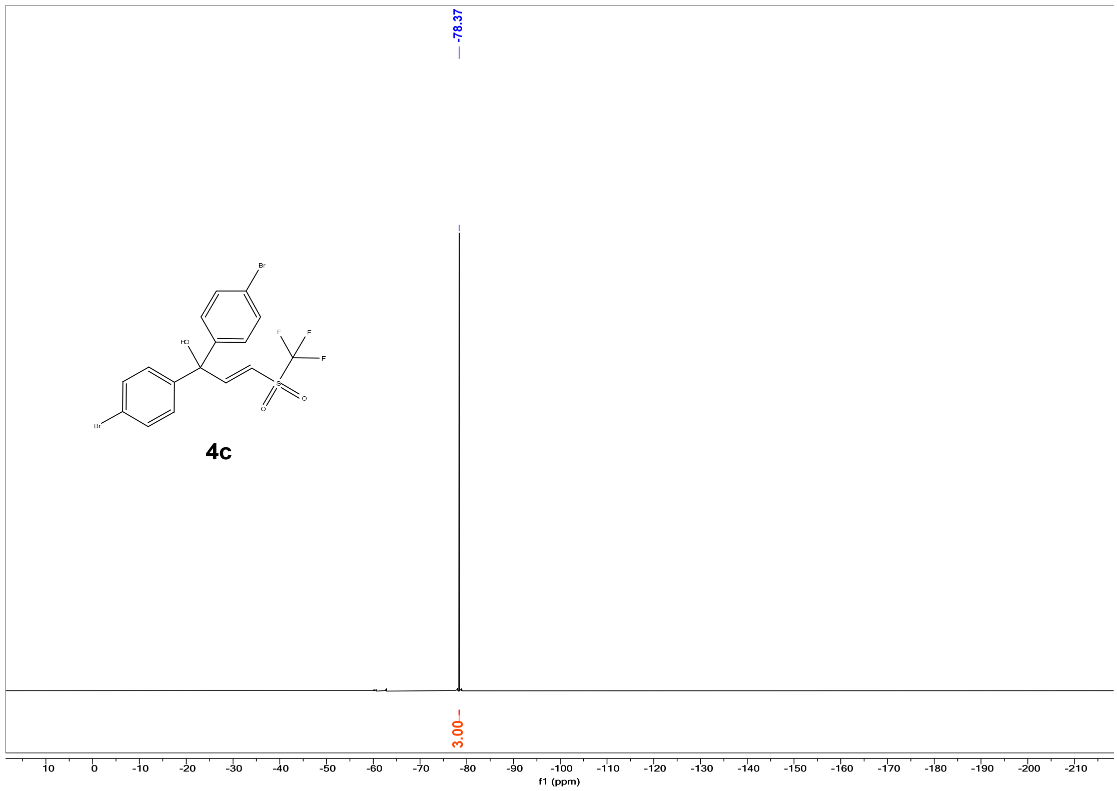
-78.37
Br
HO
F
F
F
S
O
O
Br
4c
3.00
10
0
-10
-20
-30
-40
-50
-60
-70
-80
-90
-100
-110
-120
-130
-140
-150
-160
-170
-180
-190
-200
-210
f1 (ppm)

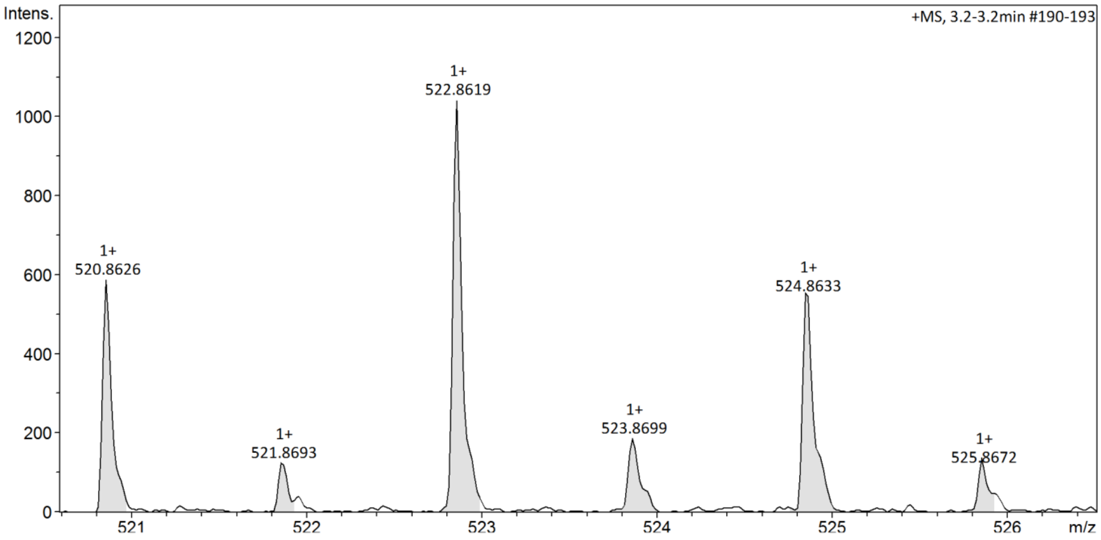
Intens.
+MS, 3.2-3.2min #190-193
1+
520.8626
1+
521.8693
1+
522.8619
1+
523.8699
1+
524.8633
1+
525.8672
0
200
400
600
800
1000
1200
521
522
523
524
525
526
m/z

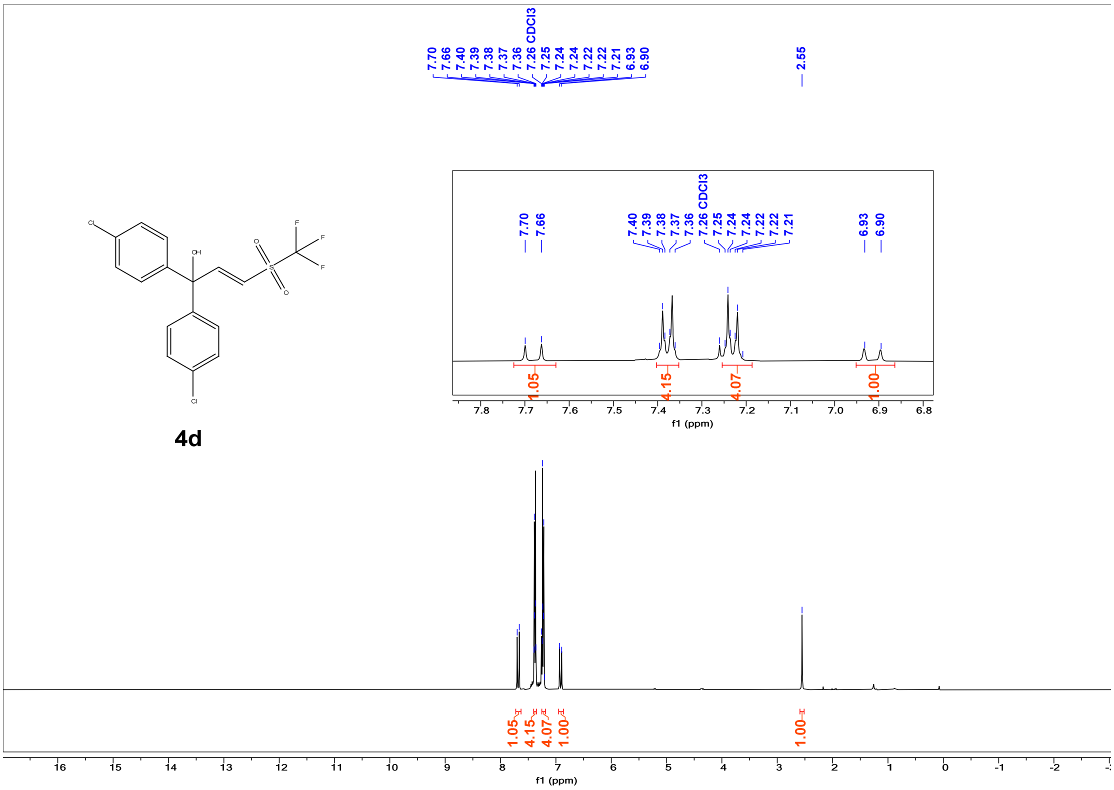
7.70
7.66
7.40
7.39
7.38
7.37
7.36
7.26 CDCl3
7.25
7.24
7.24
7.22
7.22
7.21
6.93
6.90
2.55
OH
Cl
F
O
4d
1.05
4.15
4.07
1.00
f1 (ppm)

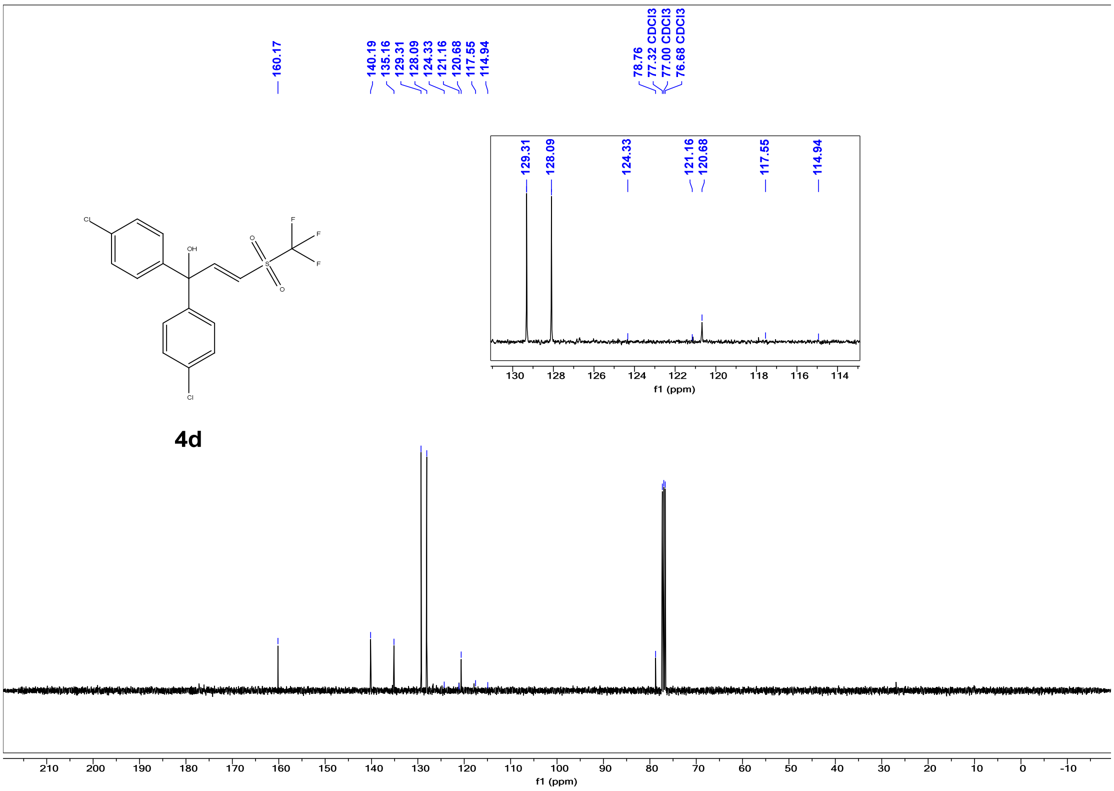
160.17
140.19
135.16
129.31
128.09
124.33
121.16
120.68
117.55
114.94
78.76
77.32 CDCl3
77.00 CDCl3
76.68 CDCl3
OH
Cl
F
O
4d
f1 (ppm)

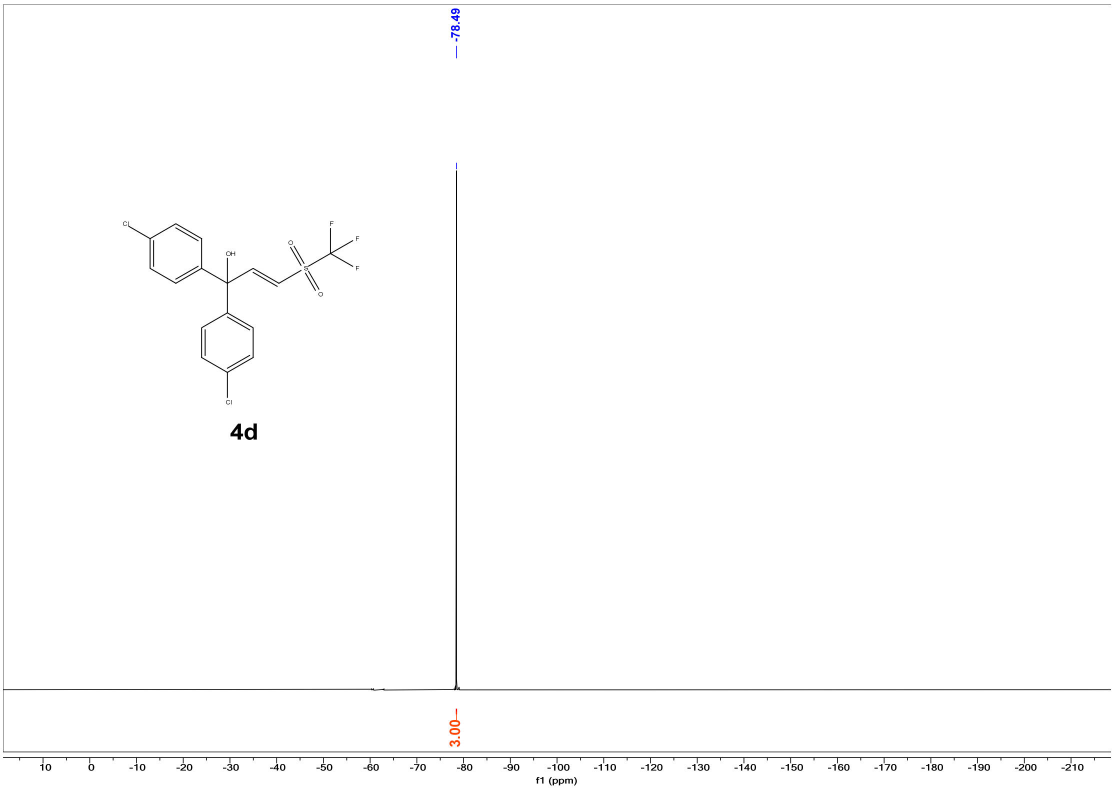

-78.49
4d
3.00
f1 (ppm)

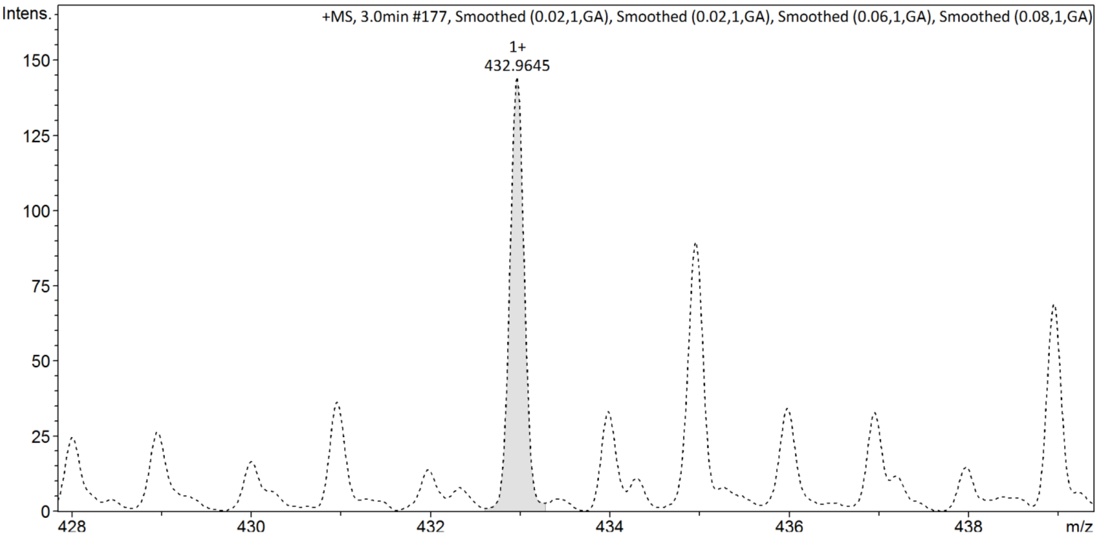

Intens.
+MS, 3.0min #177, Smoothed (0.02,1,GA), Smoothed (0.02,1,GA), Smoothed (0.06,1,GA), Smoothed (0.08,1,GA)
1+
432.9645
m/z

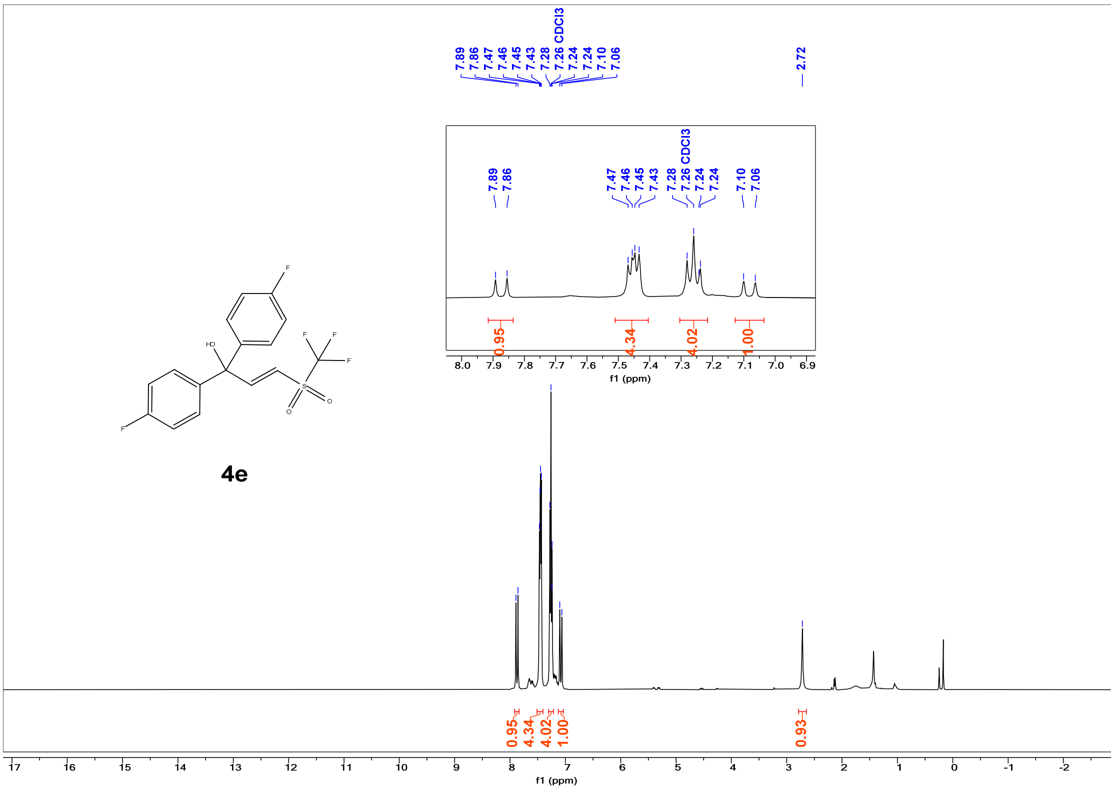
7.89
7.86
7.47
7.46
7.45
7.43
7.28
7.26 CDCl3
7.24
7.24
7.10
7.06
2.72
4e
0.95
4.34
4.02
1.00
0.93
f1 (ppm)

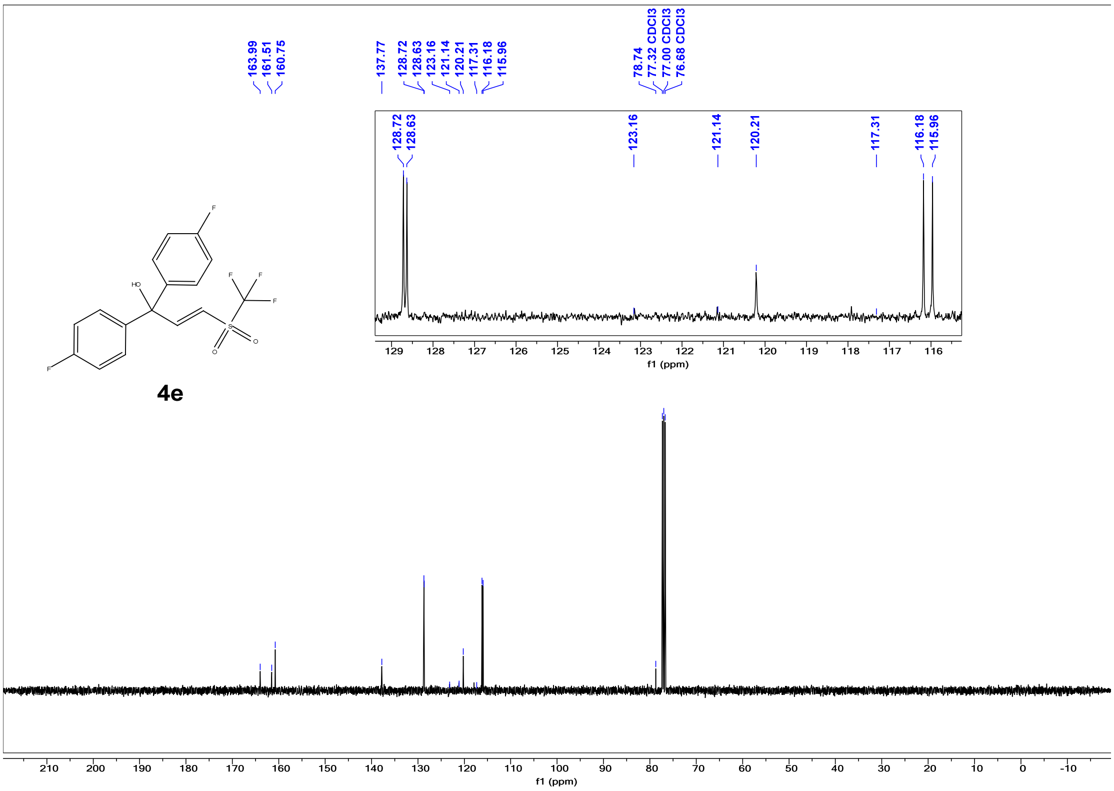
163.99
161.51
160.75
137.77
128.72
128.63
123.16
121.14
120.21
117.31
116.18
115.96
78.74
77.32 CDCl3
77.00 CDCl3
76.68 CDCl3
4e
f1 (ppm)

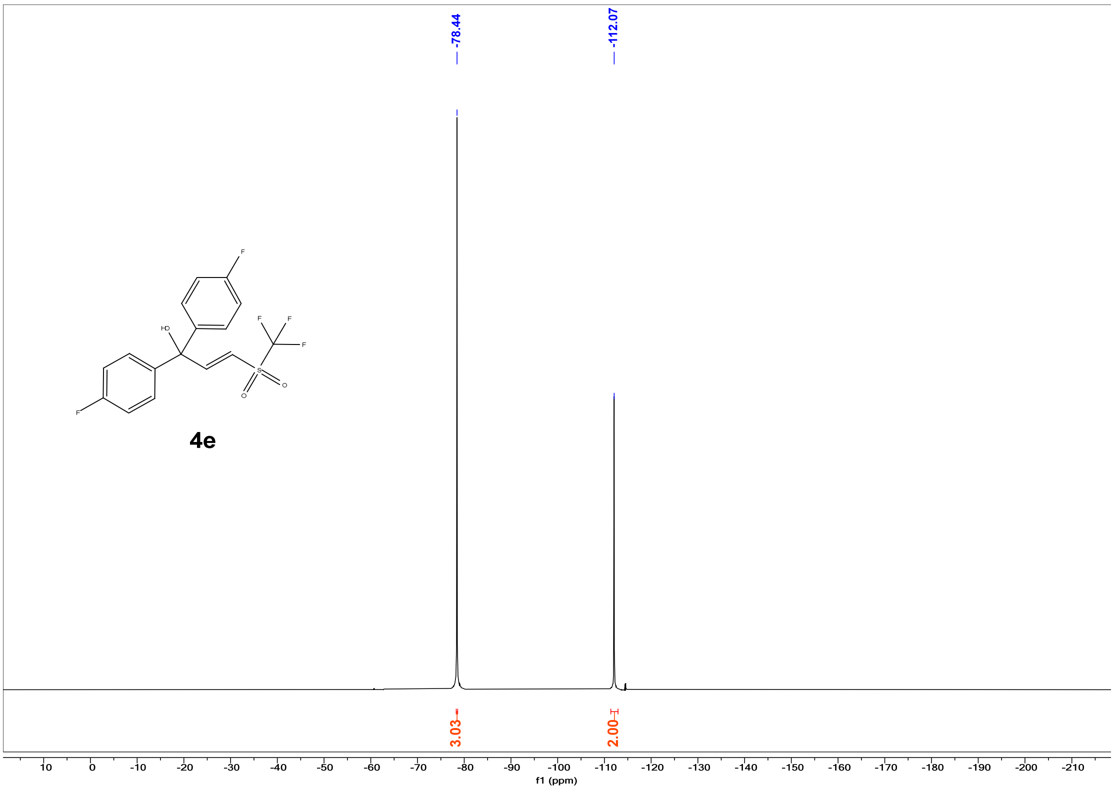

-78.44
-112.07
HO
F
4e
3.03
2.00
f1 (ppm)

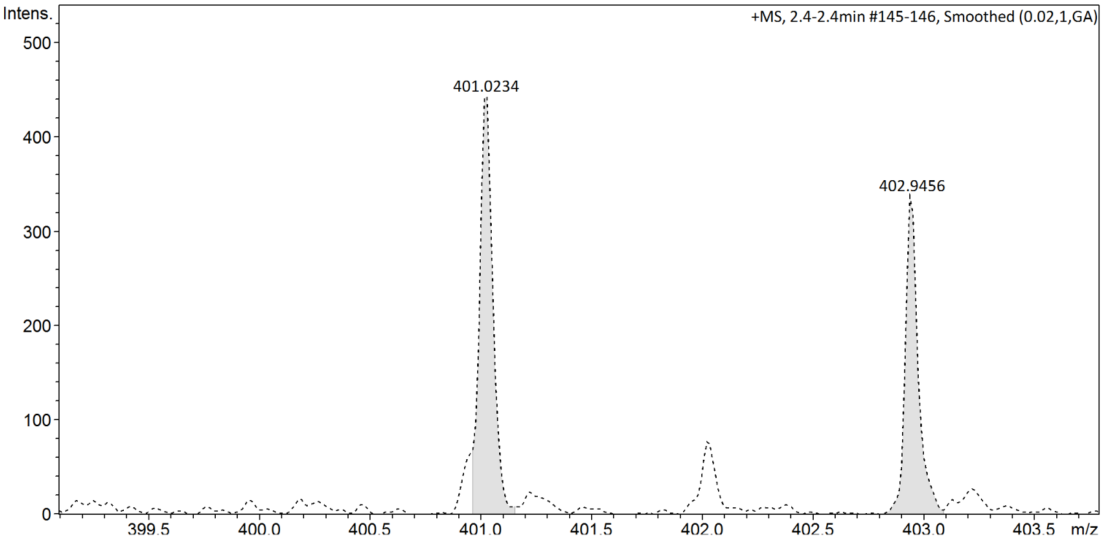

Intens.
+MS, 2.4-2.4min #145-146, Smoothed (0.02,1,GA)
401.0234
402.9456
m/z

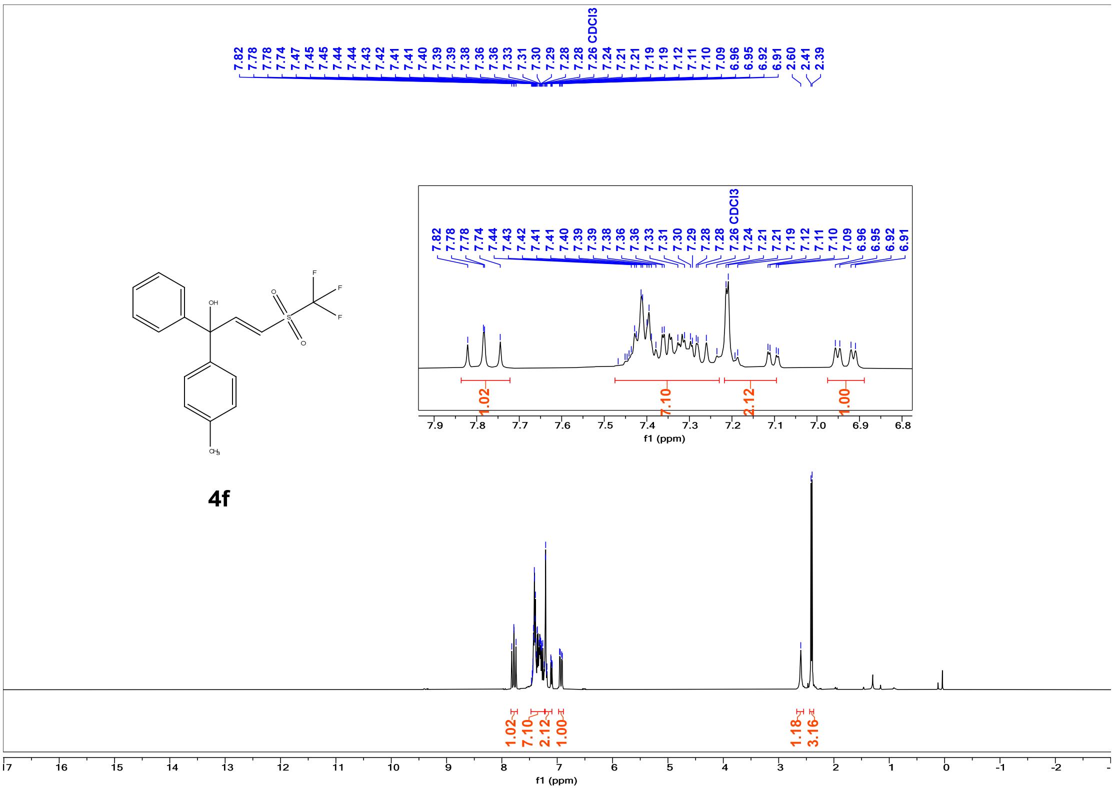
7.82 7.78 7.78 7.74 7.47 7.45 7.45 7.44 7.44 7.43 7.42 7.41 7.41 7.40 7.39 7.39 7.38 7.36 7.36 7.33 7.31 7.30 7.29 7.28 7.26 CDCl3 7.24 7.21 7.21 7.19 7.19 7.12 7.11 7.10 7.09 6.96 6.95 6.92 6.91 2.60 2.41 2.39
1.02 7.10 2.12 1.00 1.18 3.16
f1 (ppm)
4f

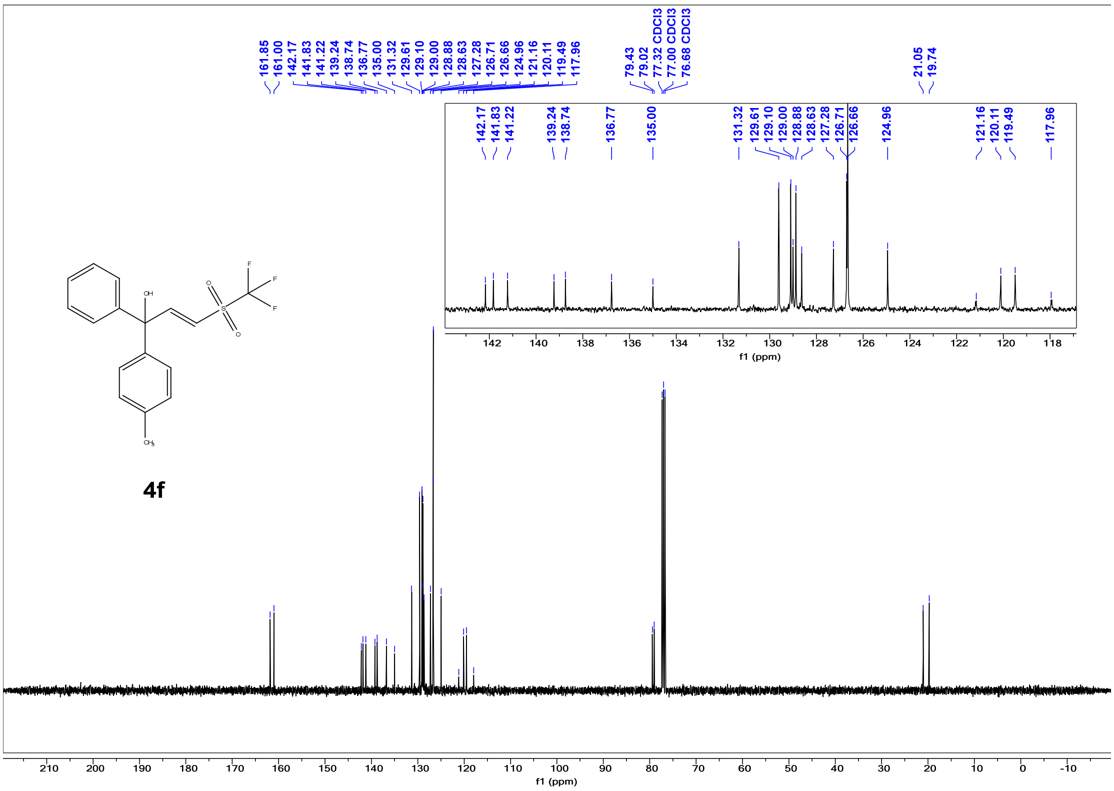
161.85 161.00 142.17 141.83 141.22 139.24 138.74 136.77 135.00 131.32 129.61 129.10 129.00 128.88 128.63 127.28 126.71 126.66 124.96 121.16 120.11 119.49 117.96
79.43 79.02 77.32 CDCl3 77.00 CDCl3 76.68 CDCl3
21.05 19.74
f1 (ppm)
4f

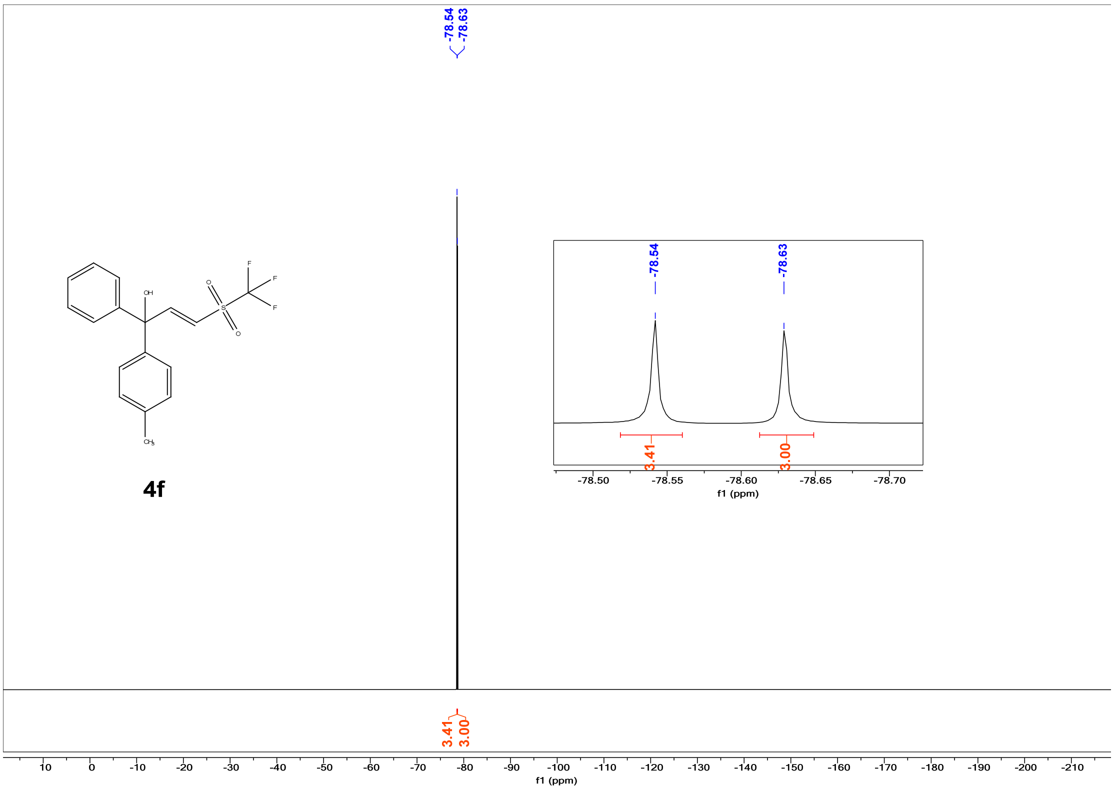
-78.54
-78.63
3.41
3.00
f1 (ppm)

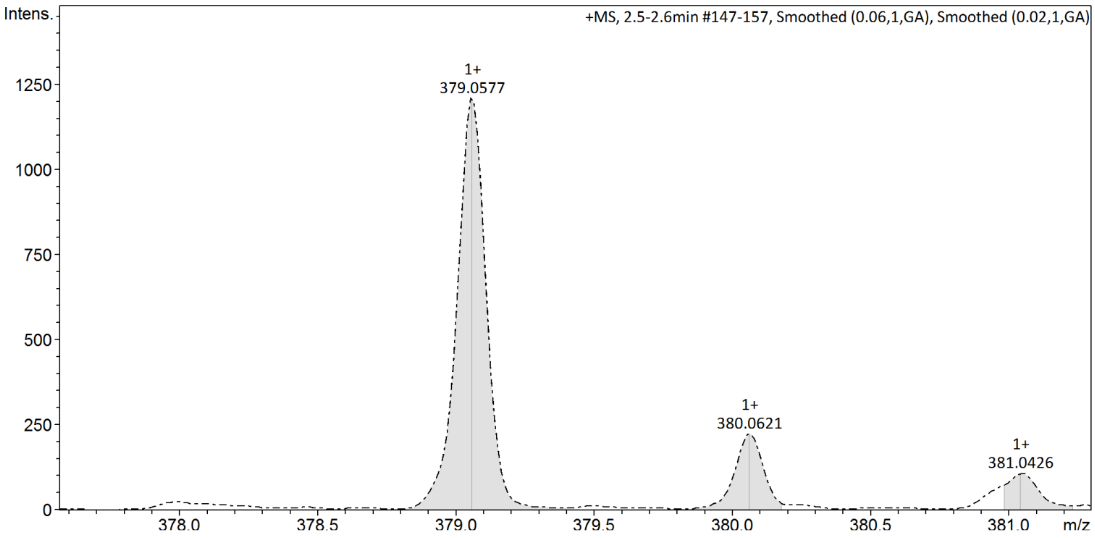
Intens.
+MS, 2.5-2.6min #147-157, Smoothed (0.06,1,GA), Smoothed (0.02,1,GA)
1+
379.0577
1+
380.0621
1+
381.0426
m/z

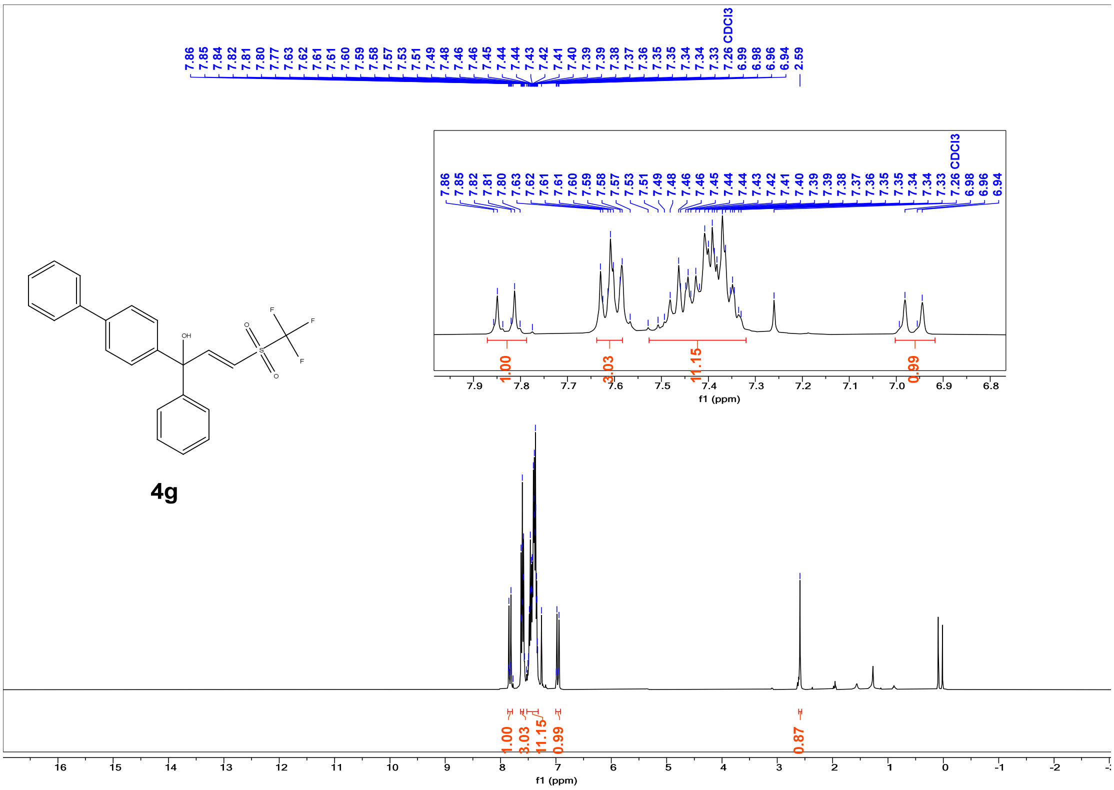
7.86 7.85 7.84 7.82 7.81 7.80 7.77 7.63 7.62 7.61 7.61 7.60 7.59 7.58 7.57 7.53 7.51 7.49 7.48 7.46 7.45 7.44 7.44 7.43 7.42 7.41 7.40 7.39 7.39 7.38 7.37 7.36 7.35 7.35 7.34 7.34 7.33 7.26 CDCl3 6.99 6.98 6.96 6.94 2.59
OH
O
S
O
F
F
F
1.00
3.03
11.15
0.99
0.87
f1 (ppm)

4g

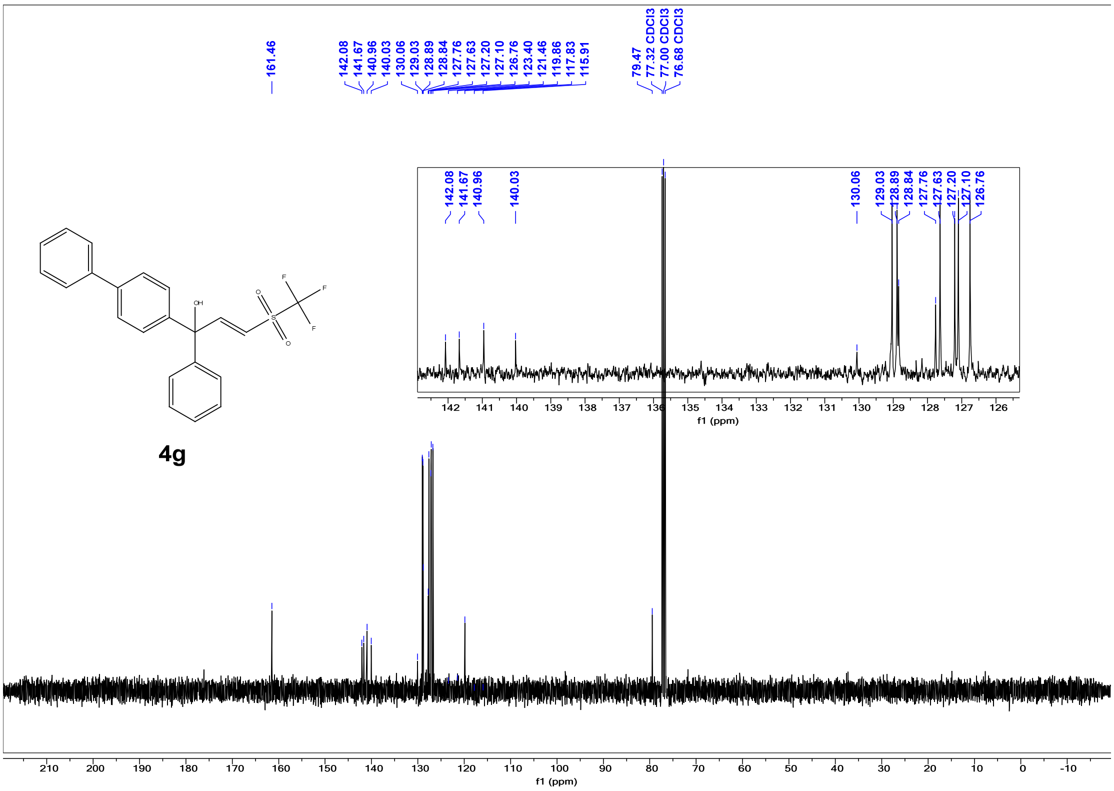
161.46
142.08 141.67 140.96 140.03 130.06 129.03 128.89 128.84 127.76 127.63 127.20 127.10 126.76 123.40 121.46 119.86 117.83 115.91
79.47 77.32 CDCl3 77.00 CDCl3 76.68 CDCl3
OH
O
S
O
F
F
F
f1 (ppm)

4g

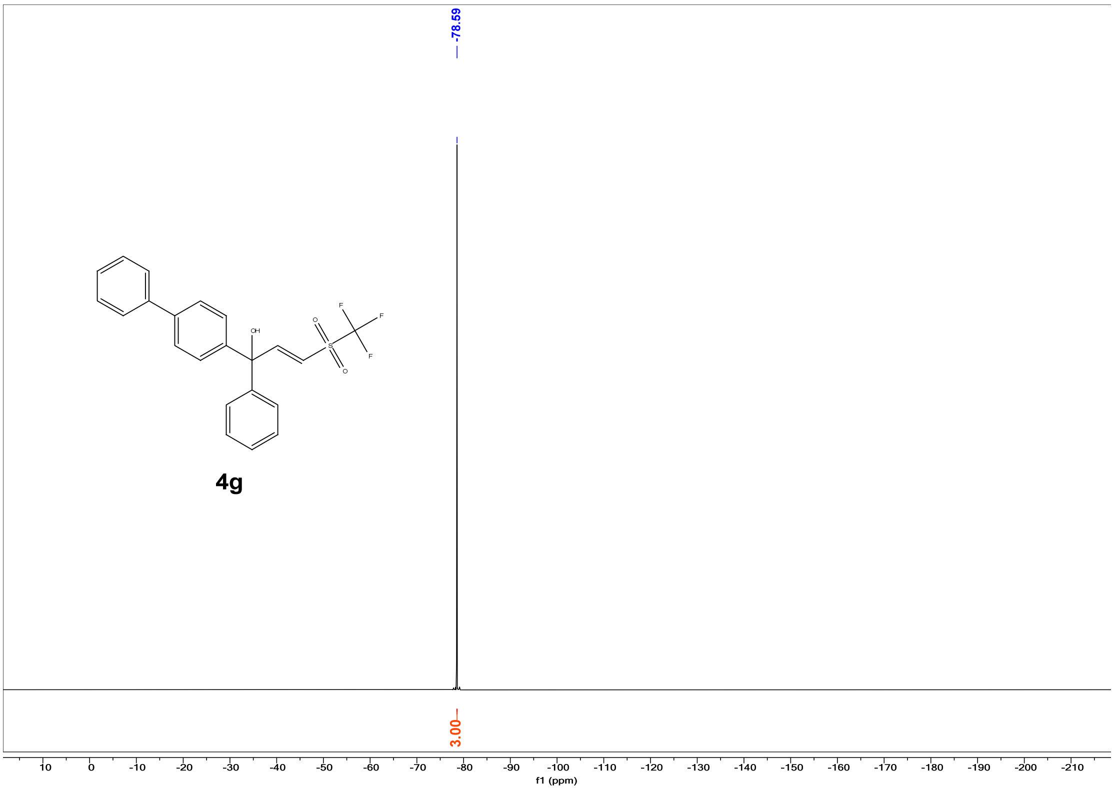
-78.59
4g
3.00
10 0 -10 -20 -30 -40 -50 -60 -70 -80 -90 -100 -110 -120 -130 -140 -150 -160 -170 -180 -190 -200 -210
f1 (ppm)

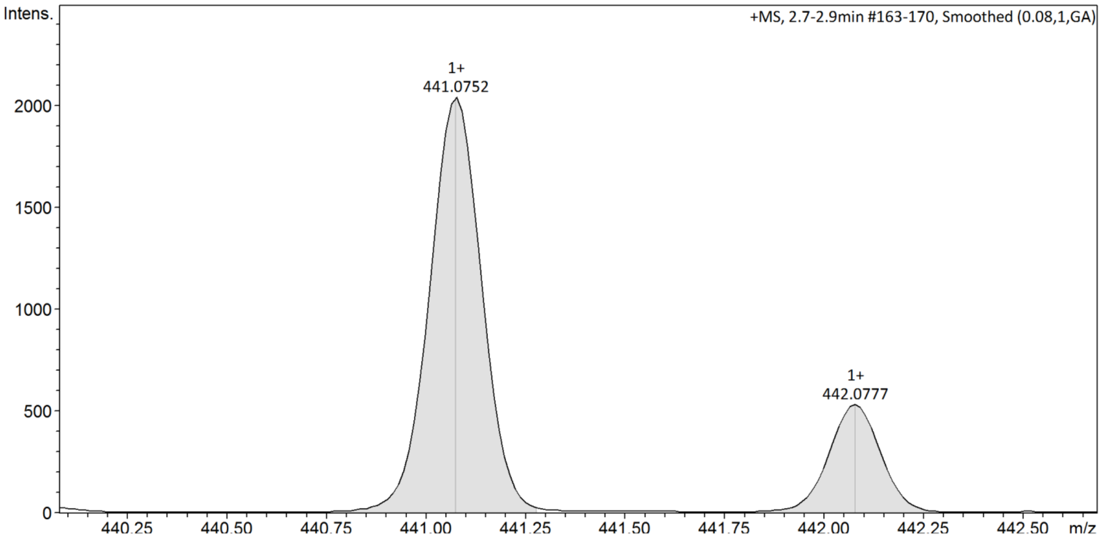
Intens.
+MS, 2.7-2.9min #163-170, Smoothed (0.08,1,GA)
1+
441.0752
1+
442.0777
2000
1500
1000
500
0
440.25 440.50 440.75 441.00 441.25 441.50 441.75 442.00 442.25 442.50 m/z

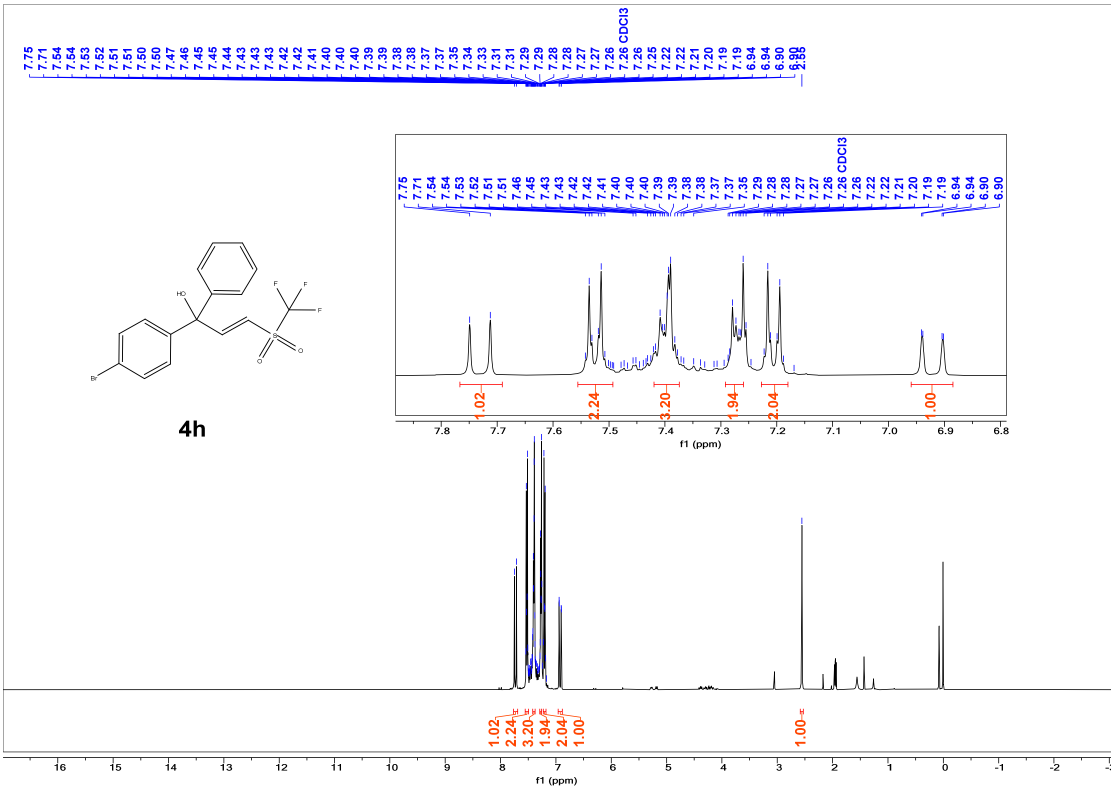

7.75 7.71 7.54 7.54 7.53 7.52 7.51 7.51 7.50 7.47 7.46 7.45 7.45 7.44 7.43 7.43 7.43 7.42 7.42 7.41 7.40 7.40 7.40 7.39 7.39 7.38 7.38 7.37 7.37 7.35 7.34 7.33 7.31 7.29 7.29 7.28 7.28 7.27 7.27 7.26 CDCl3 7.26 7.25 7.22 7.22 7.21 7.20 7.19 7.19 6.94 6.94 6.90 6.90 2.55
4h
1.02 2.24 3.20 1.94 2.04 1.00
1.00
f1 (ppm)

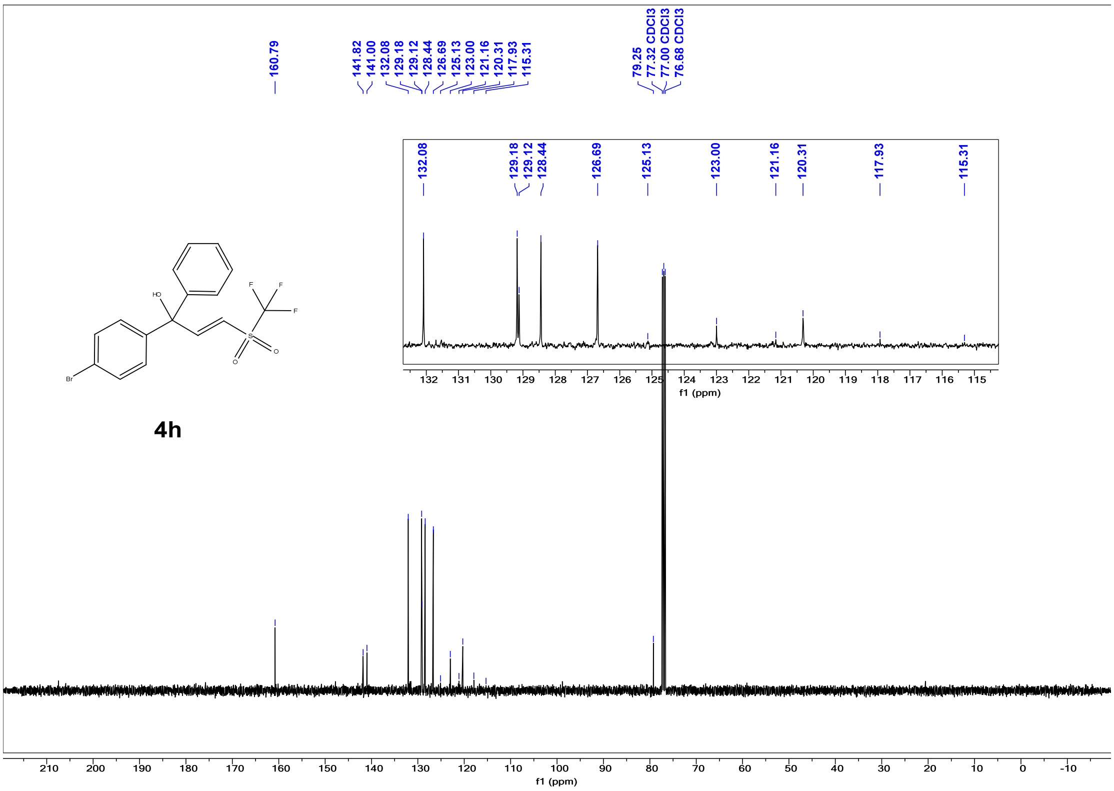

160.79
141.82 141.00 132.08 129.18 129.12 128.44 126.69 125.13 123.00 121.16 120.31 117.93 115.31
79.25 77.32 CDCl3 77.00 CDCl3 76.68 CDCl3
4h
f1 (ppm)

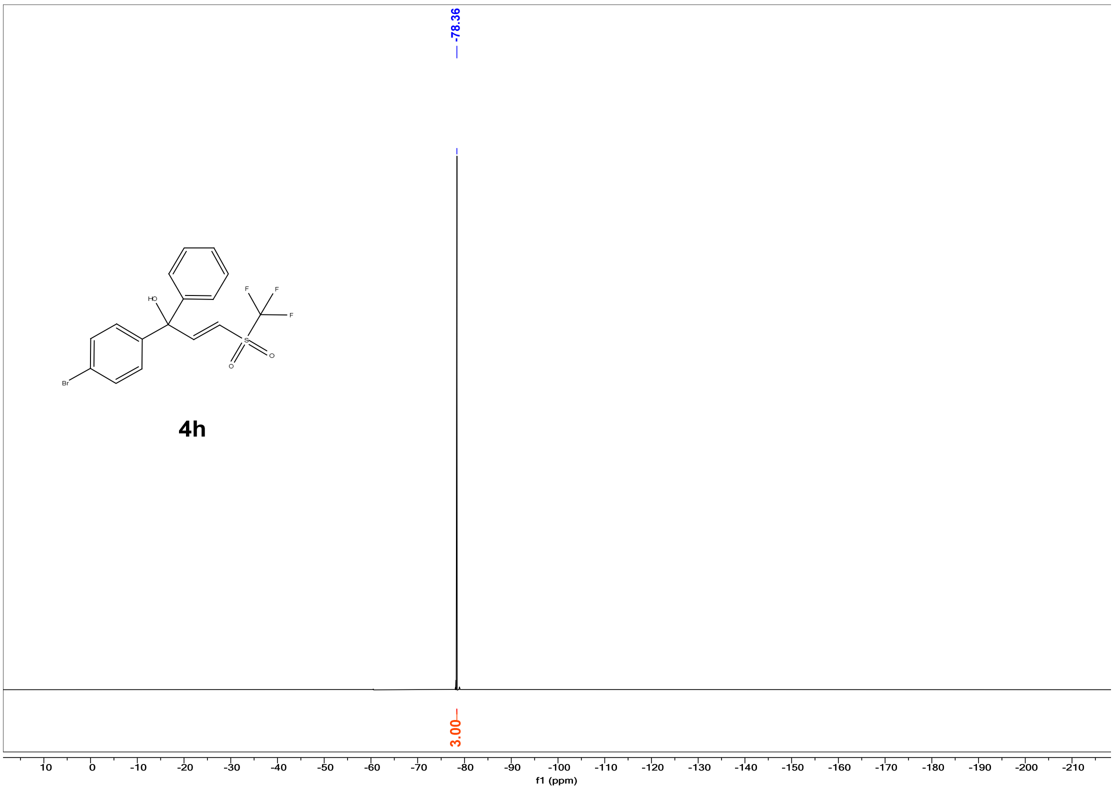

-78.36
4h
3.00
10
0
-10
-20
-30
-40
-50
-60
-70
-80
-90
-100
-110
-120
-130
-140
-150
-160
-170
-180
-190
-200
-210
f1 (ppm)

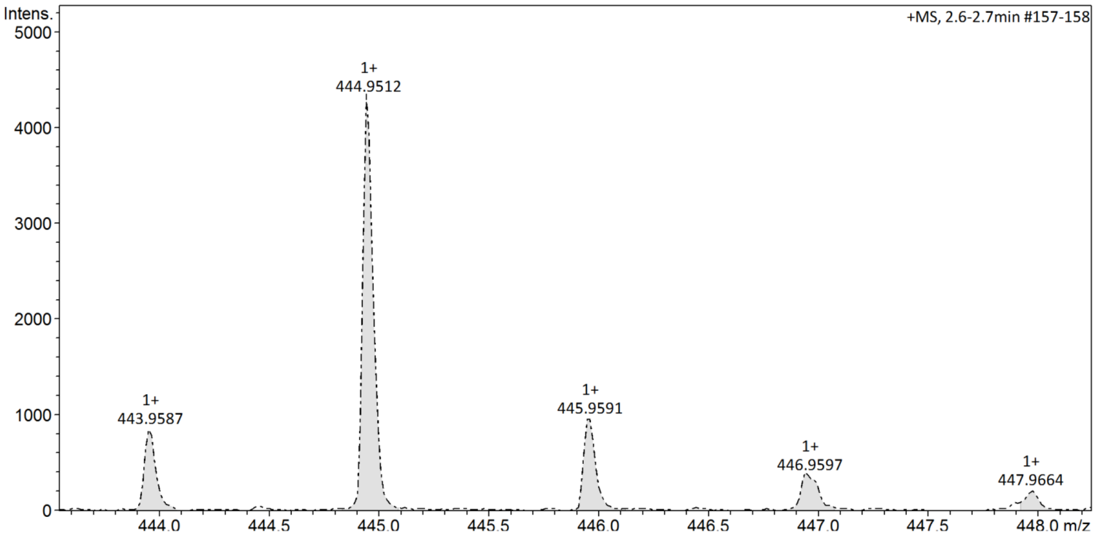

Intens.
+MS, 2.6-2.7min #157-158
1+
444.9512
1+
443.9587
1+
445.9591
1+
446.9597
1+
447.9664
5000
4000
3000
2000
1000
0
444.0
444.5
445.0
445.5
446.0
446.5
447.0
447.5
448.0 m/z

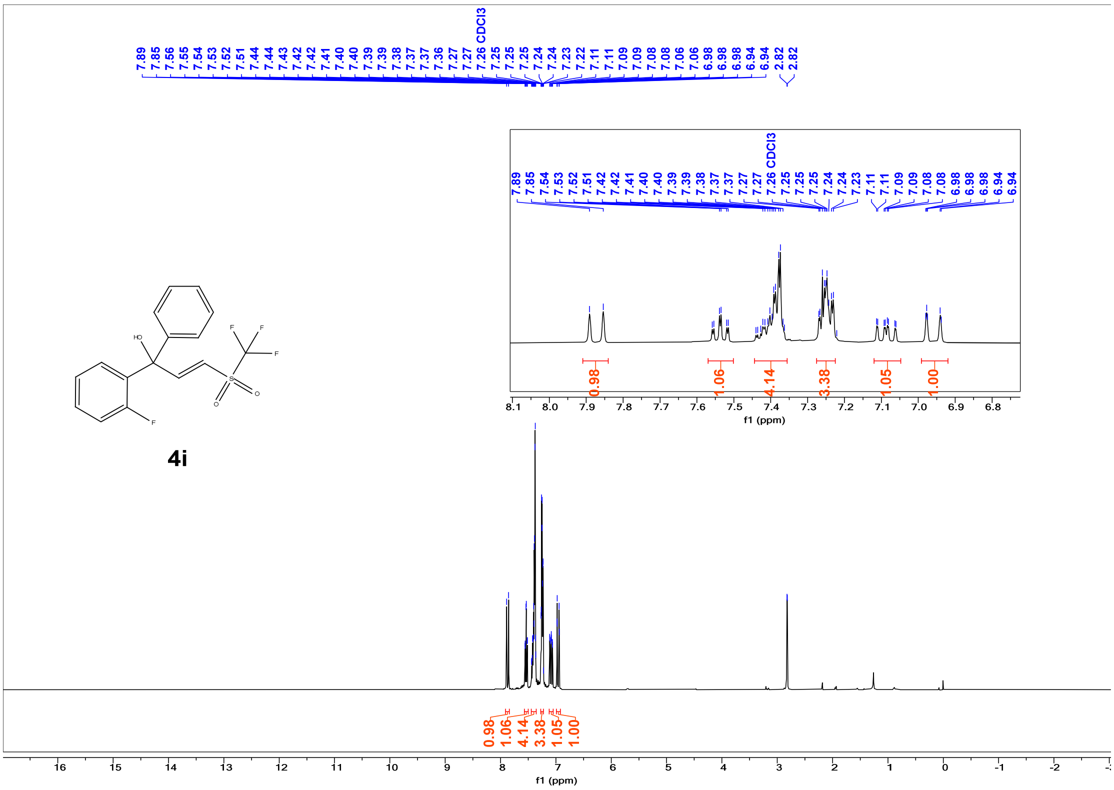
7.89 7.85 7.56 7.55 7.54 7.53 7.52 7.51 7.44 7.43 7.42 7.42 7.41 7.40 7.40 7.39 7.39 7.38 7.37 7.37 7.36 7.27 7.27 7.26 CDCl3 7.25 7.25 7.25 7.24 7.24 7.23 7.22 7.11 7.11 7.09 7.09 7.08 7.08 7.06 7.06 6.98 6.98 6.98 6.98 6.94 6.94 2.82 2.82
0.98 1.06 4.14 3.38 1.05 1.00
f1 (ppm)
HO
F
S
O
4i

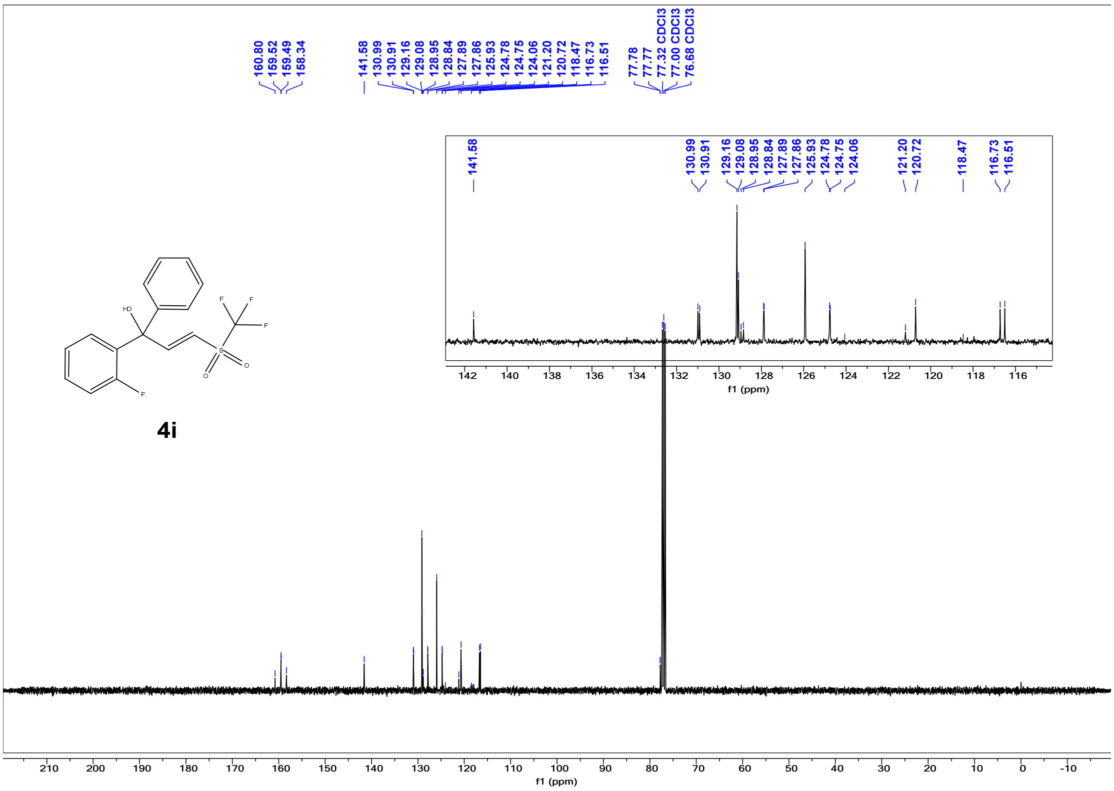
160.80 159.52 159.49 158.34 141.58 130.99 130.91 129.16 129.08 128.95 128.84 127.89 127.86 125.93 124.78 124.75 124.06 121.20 120.72 118.47 116.73 116.51 77.78 77.77 77.32 CDCl3 77.00 CDCl3 76.68 CDCl3
f1 (ppm)
HO
F
S
O
4i

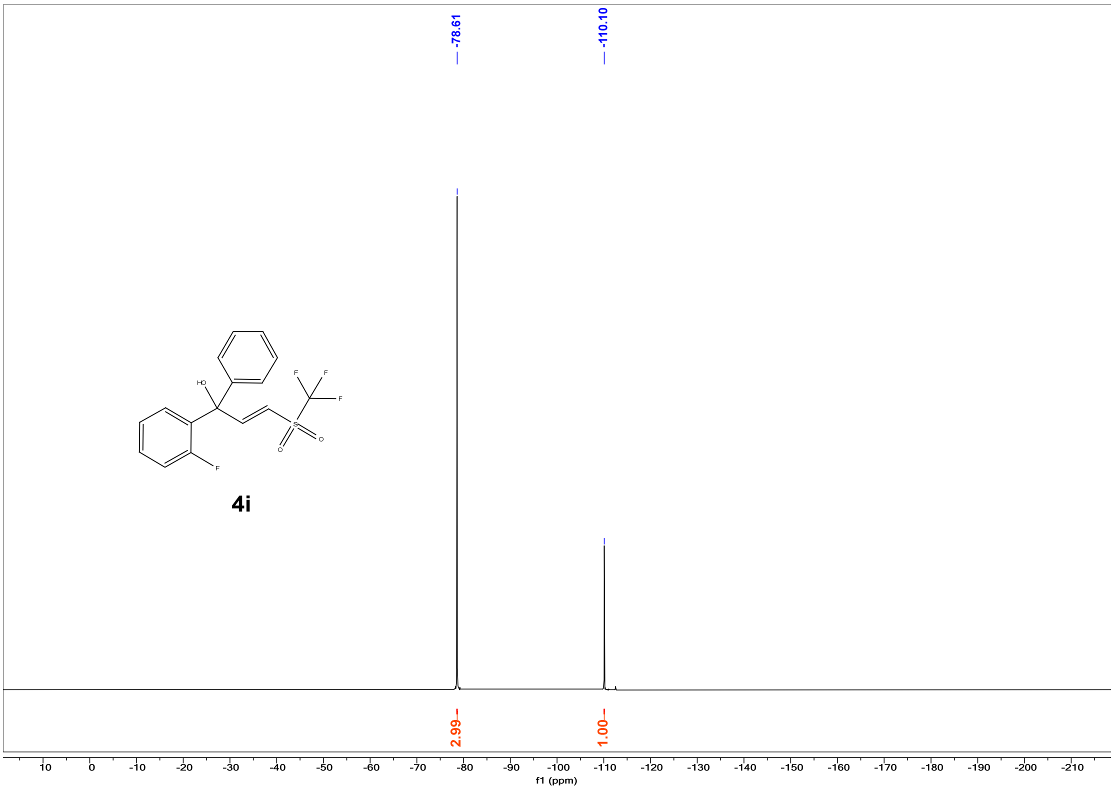
-78.61
-110.10
HO
F
F
F
S
O
O
F
4i
2.99
1.00
10
0
-10
-20
-30
-40
-50
-60
-70
-80
-90
-100
-110
-120
-130
-140
-150
-160
-170
-180
-190
-200
-210
f1 (ppm)

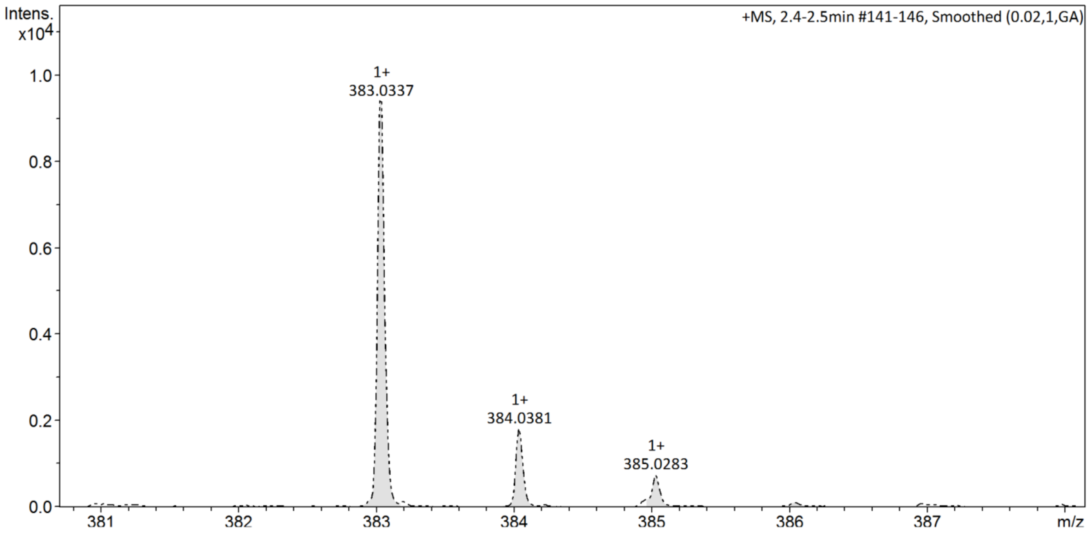
Intens.
x10^4
+MS, 2.4-2.5min #141-146, Smoothed (0.02,1,GA)
1.0
0.8
0.6
0.4
0.2
0.0
1+
383.0337
1+
384.0381
1+
385.0283
381
382
383
384
385
386
387
m/z

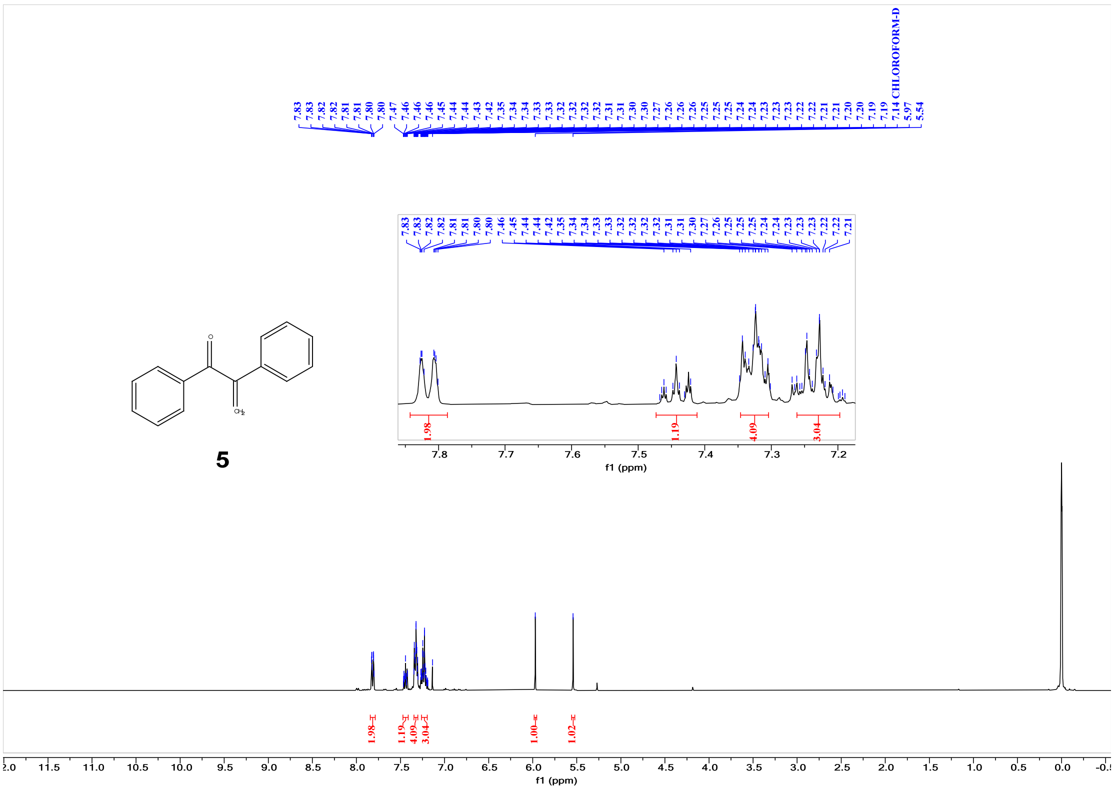
5
f1 (ppm)

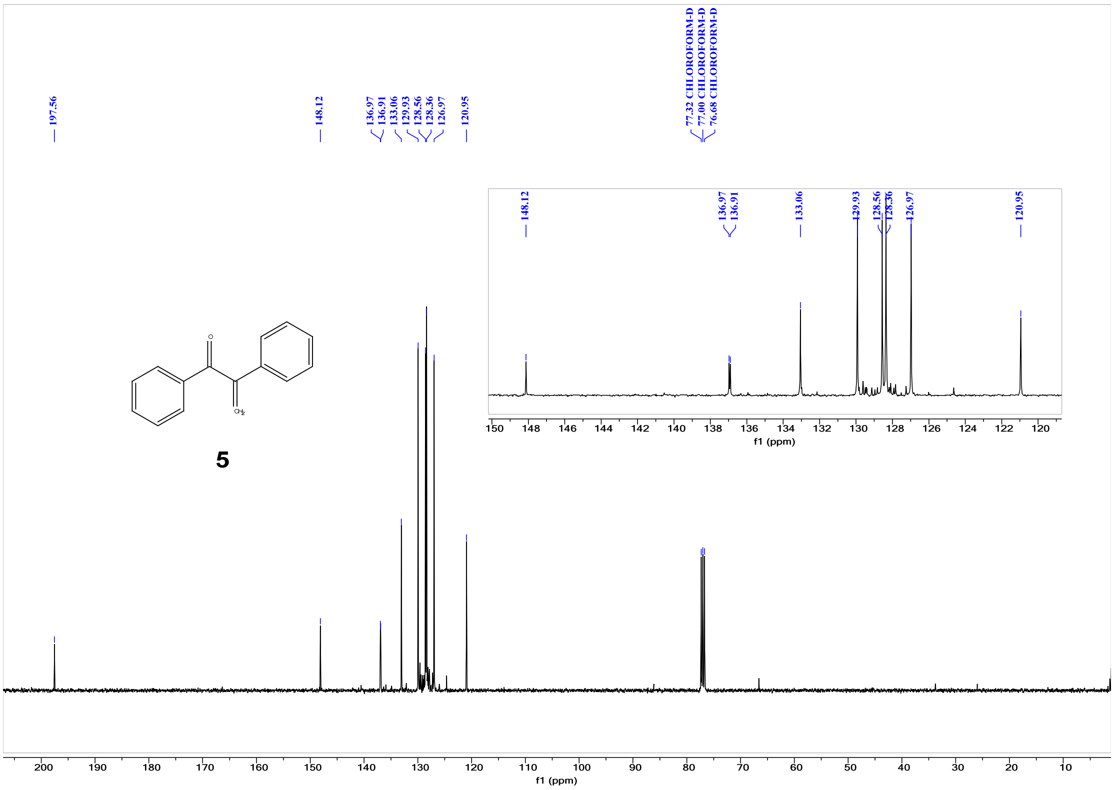
5
f1 (ppm)
